# Effect of air pollution on disease burden, mortality, and life expectancy in North Africa and the Middle East: a systematic analysis for the Global Burden of Disease Study 2019

**DOI:** 10.1016/S2542-5196(23)00053-0

**Published:** 2023-05-08

**Authors:** Mohsen Abbasi-Kangevari, Mohammad-Reza Malekpour, Masoud Masinaei, Sahar Saeedi Moghaddam, Seyyed-Hadi Ghamari, Zeinab Abbasi-Kangevari, Negar Rezaei, Nazila Rezaei, Ali H Mokdad, Mohsen Naghavi, Bagher Larijani, Farshad Farzadfar, Christopher J L Murray, GBD 2019 North Africa and the Middle East Air Pollution Collaborators, Mohsen Abbasi-Kangevari, Mohammad-Reza Malekpour, Masoud Masinaei, Sahar Saeedi Moghaddam, Seyyed-Hadi Ghamari, Zeinab Abbasi-Kangevari, Negar Rezaei, Nazila Rezaei, Amirali Aali, Sina Abdollahzade, Salam Abdulqadir Abdulrahman, Hiwa Abubaker Ali, Aqeel Ahmad, Rizwan Ahmad, Ali Ahmadi, Mohammad Ahmadian, Haroon Ahmed, Tarik Ahmed Rashid, Marjan Ajami, Hanadi Al Hamad, Fadwa Alhalaiqa Naji Alhalaiqa, Vahid Alipour, Sami Almustanyir, Javad Aminian Dehkordi, Sohrab Amiri, Jalal Arabloo, Judie Arulappan, Zahra Aryan, Seyyed Shamsadin Athari, Sina Azadnajafabad, Nayereh Baghcheghi, Farshad Bahrami Asl, Ovidiu Constantin Baltatu, Azadeh Bashiri, Akshaya Srikanth Bhagavathula, Ali Bijani, Saeid Bitaraf, Michael Brauer, Maria Cheraghi, Saad M A Dahlawi, Abdollah Dargahi, Reza Darvishi Cheshmeh Soltani, Mostafa Dianatinasab, Milad Dodangeh, Ebrahim Eini, Maysaa El Sayed Zaki, Hassan El-Abid, Muhammed Elhadi, Sharareh Eskandarieh, Shahab Falahi, Mohammad Fareed, Ali Fatehizadeh, Mehdi Fazlzadeh, Farhad Ghamari, Reza Ghanbari, Ahmad Ghashghaee, Abdolmajid Gholizadeh, Mohamad Golitaleb, Gholamreza Goudarzi, Mostafa Hadei, Randah R Hamadeh, Samer Hamidi, Ahmed I Hasaballah, Hamidreza Hasani, Soheil Hassanipour, Kamal Hezam, Mohammad Hoseini, Mohammad-Salar Hosseini, Mehdi Hosseinzadeh, Soodabeh Hoveidamanesh, Jalil Jaafari, Hosna Janjani, Sathish Kumar Jayapal, Laleh R Kalankesh, Rohollah Kalhor, Samad Karkhah, Neda Kaydi, Yousef Saleh Khader, Morteza Abdullatif Khafaie, Javad Khanali, Moawiah Mohammad Khatatbeh, Ali Koolivand, Mohammed Kuddus, Faris Hasan Lami, Soleiman Mahjoub, Afshin Maleki, Ahmad Azam Malik, Sahar Masoudi, Ritesh G Menezes, Shabir Ahmad Mir, Ashraf Mohamadkhani, Esmaeil Mohammadi, Mohammad Javad Mohammadi, Mokhtar Mohammadi, Fateme Montazeri, Paula Moraga, Negar Morovatdar, Christopher J L Murray, Abbas Norouzian Baghani, Keyvan Pakshir, Hamidreza Pazoki Toroudi, Meghdad Pirsaheb, Ashkan Pourabhari Langroudi, Fakher Rahim, Mehran Rahimi, Shayan Rahmani, Sina Rashedi, Azad Rasul, Elrashdy Moustafa Mohamed Redwan, Mohsen Rezaeian, Saeid Sadeghian, Amirhossein Sahebkar, Mohammad Ali Sahraian, Payman Salamati, Hedayat Salari, Abdallah M Samy, Brijesh Sathian, Kiomars Sharafi, Ali Sheikhy, Parnian Shobeiri, Zahra Shokri Varniab, Seyed Afshin Shorofi, Ensiyeh Taheri, Sahel Valadan Tahbaz, Siavash Vaziri, Mehdi Vosoughi, Kheirollah Yari, Arzu Yigit, Vahit Yigit, Leila Zaki, Iman Zare, Ahmad Zarei, Zahra Zareshahrabadi, Ali H Mokdad, Mohsen Naghavi, Bagher Larijani, Farshad Farzadfar

**Affiliations:** a Non-Communicable Diseases Research Center, Endocrinology and Metabolism Population Sciences Institute, Tehran University of Medical Sciences, Tehran, Iran; b Kiel Institute for the World Economy, Kiel, Germany; c Endocrinology and Metabolism Research Center, Endocrinology and Metabolism Clinical Sciences Institute, Tehran University of Medical Sciences, Tehran, Iran; d Institute for Health Metrics and Evaluation, University of Washington, Seattle, WA, USA; e Department of Health Metrics Sciences, School of Medicine, University of Washington, Seattle, WA, USA

## Abstract

**Background:**

Air pollution is the sixth highest risk factor for attributable disability-adjusted life-years (DALYs) in North Africa and the Middle East, but the relative importance of different subtypes of air pollution and any potential differences in their health effects by population demographics or country-level socioeconomic factors have not been fully explored. The objective of this study was to investigate the effect of high ambient particulate matter less than 2·5 μm in size (PM) and ambient ozone air pollution on disease burden, mortality, and life expectancy in 21 countries in the North Africa and the Middle East super-region from 1990 to 2019 using the Global Burden of Diseases, Injuries, and Risk Factors (GBD) Study estimates.

**Methods:**

The study data were derived from GBD 2019, examining data from 1999 to 2019 in North Africa and the Middle East. In this study, the types of air pollution investigated included PM pollution and ambient ozone pollution. PM pollution itself was categorised as household air pollution from solid fuels and ambient PM pollution. The burden attributable to each risk factor, directly or indirectly, was incorporated in the population attributable fraction to estimate the total attributable deaths and DALYs. The summary exposure value (SEV) as the relative risk-weighted prevalence of exposure was extracted to compare the distribution of excess risk times the exposure level in a population where everyone is at maximum risk and ranges from zero (no excess risk exists in a population) to 100 (highest risk). The effect of air pollution on life expectancy was estimated via a cause-deleted life table analysis.

**Findings:**

The age-standardised DALYs rate attributable to air pollution declined by 44·5%, from 4884·2 (95% uncertainty interval 4381·5–5555·4) to 2710·4 (2317·3–3125·6) per 100 000 from 1990 to 2019. Afghanistan (6992·3, 5627·7–8482·7), Yemen (4212·4, 3241·3–5418·1), and Egypt (4034·8, 3027·7–5138·6) had the highest age-standardised DALYs rates attributable to air pollution in 2019 per 100 000, whereas Türkiye (1329·2, 1033·7–1654·7), Jordan (1447·3, 1154·2–1758·5), and Iran (1603·0, 1404·7–1813·8) had the lowest rates. During the study period, the age-standardised SEV of air pollution (PM and ambient ozone in total) decreased by 10·9% (5·8–17·7%) in the super-region, whereas the SEV of ambient ozone pollution alone increased by 7·7% (0·7–14·3%). Among the components of PM pollution, the SEV of ambient PM pollution increased by 40·1% (25·2–63·7%); however, the SEV of household air pollution from solid fuels decreased by 70·6% (64·1–77·0%). Among the investigated types of air pollution, 98·9% of the DALYs from air pollution in the super-region were attributable to PM pollution. If air pollution had been lowered to the theoretical minimum risk exposure levels for 2019, then the average life expectancy would have been 1·6 years higher.

**Interpretation:**

The burden attributable to air pollution substantially decreased in the study period across the super-region as a whole. Most of the burden from air pollution is attributed to PM pollution, the exposure to which has substantially increased in the past three decades. Interventions and policies that reduce population exposure to PM pollution could potentially increase the average life expectancy in the super-region. This finding calls for concerted efforts from governments and public health authorities in the super-region to tackle air pollution as an important threat to population health.

**Funding:**

Bill & Melinda Gates Foundation.

## Introduction

Long-term air pollution exposure has deleterious effects on public health. Air pollution gives rise to local or systemic diseases with varying severity and consequently results in reduced life expectancy.^1^ It is estimated that air pollution has caused an average 1·8 years reduction in life expectancy worldwide.^2^ The types of air pollution investigated in this study are particulate matter (PM), including household air pollution from solid fuels, ambient PM pollution, and ambient ozone pollution.^3^ The burden of air pollution is strongly linked to countries’ social and economic development, and is greatest in less-developed areas, where people have double the burden of high ambient PM less than 2·5 μm in size (PM_2·5_; hereafter referred to as PM) and exposure to specific types of household air pollution.^4^

**Research in context**

**Evidence before this study**

We searched PubMed for articles published up to August, 2021, using the search terms: “air pollutants”, “air pollution”, “ambient particulate matter pollution”, “ozone concentration”, “PM_2.5_ exposure”, “household air pollution”, “indoor pollution”, “death”, “mortality”, “morbidity”, “DALY”, “life expectancy”, “burden”, “North Africa”, “Middle East”, “Afghanistan”, “Algeria”, “Bahrain”, “Egypt”, “Iran”, “Iraq”, “Jordan”, “Kuwait”, “Lebanon”, “Libya”, “Morocco”, “Oman”, “Palestine”, “Qatar”, “Saudi Arabia”, “Sudan”, “Syria”, “United Arab Emirates”, “Tunisia”, “Türkiye”, and “Yemen”, with the language restricted to English. Previous evidence suggests that long-term exposure to air pollution has deleterious effects on public health and is a notable risk factor for the disease burden in the North Africa and Middle East super-region. Although countries in this super-region have varying exposure to air pollution, no studies have systematically measured the variations among countries in deaths and disability-adjusted life-years (DALYs) attributable to air pollution and its subtypes, and the effect of air pollution on life expectancy.

**Added value of this study**

This study measures the exposure to air pollution and its effect on deaths, DALYs, and life expectancy on a national level in the North Africa and the Middle East super-region using data from the Global Burden of Diseases, Injuries, and Risk Factors Study 2019. We report the effect of particulate matter (PM) pollution, its subtypes, and ambient ozone pollution on a national level in the super-region by sex, age groups, and Socio-demographic Index levels. The findings of this study show that, although the burden attributable to air pollution decreased substantially in the super-region from 1999 to 2019, countries still have varying adverse effects from air pollution. Moreover, household air pollution from solid fuels is a substantial problem in some countries in the super-region. We estimated that the average life expectancy in North Africa and the Middle East would have been higher by 1·6 years if pollution had been lowered to the theoretical minimum risk exposure levels.

**Implications of all the available evidence**

The mortality burden attributable to air pollution has substantially lessened in the super-region overall between 1990 and 2019. The emerging challenge in air pollution is exposure to ambient PM pollution, which needs immediate attention. Our study identifies countries where notable gains in life expectancy could be achieved if exposure to PM pollution could be further reduced; our findings should help to guide the most important pollutants and environments to target in country-level interventions**.** Alongside the economic progress in the super-region, sustainable development policies and investment in air pollution control strategies need to be enforced to reduce the long-term adverse effects of air pollution on population health.

Air pollution ranks sixth among the top risk factors for attributable disability-adjusted life-years (DALYs) in North Africa and the Middle East.^3^ As a heterogeneous super-region in development, North African and Middle Eastern countries have varying exposure to PM and household air pollution from solid fuels. Although household air pollution tends to decrease steadily with increasing socio-demographic development, outdoor PM pollution increases with industrialisation. Nevertheless, such pollution declines with proper air quality management among countries with a higher Socio-demographic Index (SDI).^5^, ^6^ PM could have a wide range of sources, including energy production, households, industry, transport, waste, agriculture, desert dust, and forest fires.^7^ A substantial proportion of the PM in the North Africa and the Middle East super-region has been associated with desert dust,^8^ which is expected to increase because of climate change.^9^ Thus, multidisciplinary approaches by scientific experts on national and international levels are required to respond to the emerging crisis via sustainable solutions.

Lessons learned from air pollution reduction in countries with a higher SDI underscore the role of proper national policy actions and regulations in reducing exposure to air pollution as a risk factor.^10^, ^11^ Nevertheless, policy makers and public health authorities among countries in North Africa and the Middle East need scientific evidence about the current situation regarding the burden of air pollution for a prompt response to inform the public health response in each country. The burden imposed by air pollution subtypes, which age groups have the highest burden, and any potential differences by sex need to be examined. Moreover, the effect of air pollution in reducing life expectancy needs to be quantified.

Thus, the objective of this study was to investigate the effect of PM and ambient ozone air pollution on disease burden, mortality, and life expectancy in the North Africa and the Middle East super-region from 1990 to 2019, using the Global Burden of Diseases, Injuries, and Risk Factors (GBD) Study estimates.

## Methods

### Data source

The study data were derived from GBD 2019, which encompasses high-quality estimations on crucial epidemiological measures for 286 causes of death, 369 diseases and injuries, and 87 risk factors in 204 countries and territories. North Africa and the Middle East, one of the seven GBD super-regions and 21 GBD regions, were investigated in this study, and included Afghanistan, Algeria, Bahrain, Egypt, Iran, Iraq, Jordan, Kuwait, Lebanon, Libya, Morocco, Oman, Palestine, Qatar, Saudi Arabia, Sudan, Syria, Tunisia, Türkiye, the United Arab Emirates (UAE), and Yemen.

In this study, the types of air pollution investigated included PM pollution and ambient ozone pollution. PM pollution itself was categorised as household air pollution from solid fuels and ambient PM pollution. In GBD 2019, exposure to ambient PM pollution, reported in μg/m^3^, was defined as the population-weighted annual average mass concentration of PM in a cubic metre of air. The Data Integration Model for Air Quality was used to estimate ambient air pollution exposure. The Data Integration Model for Air Quality integrates data from satellite-based measurements of aerosol optical depth, ground measurements from 9960 PM monitoring stations across 108 countries, and chemical transport model simulations.^12^, ^13^ Exposure to household air pollution from solid fuels was estimated from both the proportion of individuals using solid cooking fuels and the amount of PM air pollution these individuals were exposed to. Solid fuels included coal, wood, charcoal, dung, and agricultural residues. The information was extracted on the use of solid fuels from standard multi-country survey series including the WHO Energy Database, Demographic, and Health Surveys; Living Standards Measurement Surveys; Multiple Indicator Cluster Surveys; and World Health Surveys, as well as censuses and country-specific survey series. The sources that did not distinguish specific primary fuel types, estimated fuel used for purposes other than cooking (eg, lighting or heating), did not report standard error or sample size, had more than 15% of households with missing responses, reported fuel use in physical units, or were secondary sources referencing primary analyses were excluded from the analysis.^3^

The data on the relative risks estimates of specific health outcomes associated with household PM and ambient PM and ozone were extracted from GBD 2019 via the Global Health Data Exchange, a website developed by the Institute for Health Metrics and Evaluation (appendix p 3). The level 3 causes for which air pollution was a known risk factor by GBD included blindness and vision loss; chronic obstructive pulmonary disease (COPD); diabetes; diarrhoeal diseases; encephalitis; ischaemic heart disease; lower respiratory infections; meningitis; neonatal disorders; otitis media; stroke; sudden infant death syndrome; tracheal, bronchus, and lung cancer; and upper respiratory infections. Overall, there were 108 sources for analysing the air pollution burden in North Africa and the Middle East, as presented in the appendix (p 3).

### Burden attributable to air pollution

The reduction in the current disease burden that would have been feasible if population exposure to air pollution over the study period had been at a counterfactual lower distribution of air pollution was defined as the attributable burden. The six-step framework of the comparative risk assessment was used to estimate the burden attributable to air pollution,^3^, ^14^ which included: (1) the incorporation of the risk–outcome pairs into the analysis, (2) relative risk estimation as a function of exposure, (3) exposure levels and distributions estimation, (4) computation of the theoretical minimum risk exposure level (TMREL)^15^ as the counterfactual level of exposure, (5) calculation of population attributable fractions and attributable burden, and (6) and estimation of the mediating effects of various risk factors on each other to establish the burden of a combination of risk factors.

#### Ambient PM pollution

Ambient PM pollution exposure was estimated by looking at the annual average mass concentration of particles with an aerodynamic diameter of less than 2·5 μm/m^3^ of air, weighted by the population. Data used to estimate exposure to ambient PM pollution were derived from various sources, including satellite observations of atmospheric aerosols, ground measurements, chemical transport model simulations, population estimates, and land use data.

TMREL was established using a uniform distribution with lower and upper bounds established by the average of the minimum and fifth percentiles of the exposure distributions from outdoor air pollution cohort studies conducted in North America. It was assumed that the current data were insufficient to accurately characterise the concentration*–*response function at less than the fifth percentile of the exposure distributions, which is why a uniform distribution was used rather than a fixed value. This approach aimed to represent the uncertainty regarding how much scientific evidence is consistent with the adverse effects of exposure.

Outdoor air pollution cohort studies were selected on the basis of the criteria that the fifth percentile of their exposure distribution was lower than the fifth percentile of the American Cancer Society Cancer Prevention II cohort, which was 8·2 μg/m^3^.^16^ This criteria were selected since the minimum (5·8 μg/m^3^) and fifth percentiles used by GBD 2010 were solely from the American Cancer Society Cancer Prevention II cohort. The resulting lower bound of the distribution for GBD 2019 was 2·4 and the upper bound was 5·9, and this has been unchanged since GBD 2015.

#### Household air pollution

Household air pollution exposure was estimated on the basis of both the proportion of individuals who use solid cooking fuels and the amount of PM air pollution exposure for these individuals. In our analysis, solid fuels included coal, wood, charcoal, dung, and agricultural residues. We obtained information on the use of solid fuels from standard multi-country survey series. To fill in the gaps in data from surveys and censuses, we also retrieved and updated estimates from the WHO Energy Database and conducted a systematic review of the literature. Each nationally or subnationally representative data point provided an estimate for the percentage of households using solid cooking fuels. The study used a three-step modelling approach to estimate household air pollution at the individual level. The first step involved a mixed-effect linear regression model that incorporated maternal education, urban population proportion, and GBD region and super-region as random effects. The second step involved spatiotemporal regression, and the third step involved Gaussian process regression. No significant changes were made to the model compared with the previous GBD round in 2017. For cataracts, the TMREL was defined as no households using solid cooking fuel (because any level of continuous exposure to PM from solid fuels is a risk for cataracts). For outcomes related to both ambient and household air pollution, the population attributable fractions were estimated jointly, and the TMREL was defined as a uniform distribution between 2·4 and 5·9 ug/m^3^ PM.

#### Ambient ozone pollution

To estimate the global distribution of exposure to ozone in ambient air in our study super-region from 1990 to 2019, we combined ozone ground measurement data with chemical transport model estimates using Bayesian maximum entropy. We used the highest seasonal average of 8-h daily maximum ozone concentrations, measured in parts per billion (ppb). The TMREL for ozone exposure was based on the exposure distribution observed in the American Cancer Society Cancer Prevention II study,^16^ as was the case in GBD 2017. The TMREL was a uniform distribution centred around the minimum and fifth percentile values observed in the study period, with a range of approximately 29·1–35·7 ppb.

### SDI

The SDI, a composite indicator of overall socioeconomic development status, was used in the GBD 2019. SDI was calculated on the basis of the educational attainment of individuals aged 15 years or older, lag-distributed income per person, and the total fertility rate among females younger than 25 years. The SDI ranged from 0 to 1, where higher values indicated higher development. According to SDI quintiles, countries were divided into five groups: low SDI, low-middle SDI, middle SDI, high-middle SDI, and high SDI.

### Summary exposure value (SEV)

The SEV, which is the risk-weighted prevalence of exposure, was defined as the following:

$$SEV=\frac{\int_{x=1}^{u}RR\left(x\right)P\left(x\right)dx-1}{{\text{RR}}_{\text{max}}-1}$$

Where (*x*) is the level *x* of exposure relative risk, RR_max_ is the relative risk at the 99th percentile of the global exposure distribution, and *P*(*x*) is the density of exposure at level *x*. The SEV ranged from 0 to 100, where the value 0 means there was no excess risk for the entire population, and the value 100 suggested that everyone in the population was exposed to the maximum risk.^17^

### Air pollution effect on life expectancy

The effect of air pollution on life expectancy was estimated via cause-deleted life tables,^18^, ^19^ which were computed via hypothetically considering the health years lost associated with concentrations of ambient and household PM air pollution lower than the TMREL. For each air pollution-related risk, a hypothetical life table was computed in which that particular factor was eliminated as a risk for premature death. The probability of surviving from each year of age to the next slightly increased by deleting specific risk factors. The difference between the current life expectancy and the calculated life expectancy represented the estimated gain in life expectancy from reducing exposure to air pollution.^20^, ^21^

### Statistical analysis

To investigate the effect of change in the population structure, age-standardised death and DALYs rates were computed by direct standardisation of the global age structure and presented as the number per 100 000. The 95% uncertainty interval (UI) was calculated for each metric using the 25th and 95th ranked draws of the uncertainty distribution by taking 1000 samples from the posterior distribution. Statistical analysis and data visualisations were performed using Python programming language version 3.6 and Tableau Desktop version 2020.1, an interactive data visualisation software.

### Role of the funding source

The funder of the study had no role in study design, data collection, data analysis, data interpretation, or writing of the report.

## Results

Overall, a total of 398 559 (95% UI 339 434–463 723) deaths were attributed to air pollution (appendix p 5), accounting for 12·8% (11·6–14·2%) of all deaths in the North Africa and the Middle East super-region in 2019 (appendix p 7). 391 042 (98·1%; 332 909–454 990) of the total deaths from air pollution in the super-region were attributable to PM pollution (appendix p 5). The age-standardised death rate attributable to air pollution decreased by 37·6%, from 158·3 (95% UI 139·1–177·8) to 98·8 (84·7–114·7) per 100 000 from 1990 to 2019 (appendix p 4). A total of 13 056 864 (11 145 813–15 128 048; appendix p 8) DALYs were attributed to air pollution in 2019, accounting for 8·0% (95% UI 7·0–9·1%) of all DALYs (appendix p 9). Age-standardised DALYs rate attributable to air pollution declined by 44·5%, from 4884·2 (4381·5–5555·4) to 2710·4 (2317·3–3125·6) per 100 000 in the super-region from 1990 to 2019 (appendix p 4). 98·9% of the DALYs from air pollution in the super-region were attributable to PM pollution (12 913 831; 11 041 330–14 981 099; appendix p 8). The all-ages death number and DALYs attributed to subtypes of air pollution are presented in the appendix (pp 5, 8).

Afghanistan (238·3, 95% UI 189·8–290·1 per 100 000), Egypt (159·7, 119·2–203·0), and Yemen (154·0, 116·3–202·0) had the highest age-standardised death rates attributable to air pollution in 2019, whereas Türkiye (53·3, 40·8–68·5), Jordan (56·3, 45·0–69·7), and Kuwait (65·0, 53·0–79·4) had the lowest rates (appendix pp 4, 6, 16–18). The pattern of age-standardised death rates attributed to PM pollution was similar to that of air pollution. Afghanistan (179·5, 123·3–242·1), Yemen (69·6, 39·4–106·3), and Sudan (59·1, 34·8–90·7) were the three countries in the super-region with the highest age-standardised death rates attributable to household air pollution from solid fuels in 2019 (appendix p 6). Afghanistan (6992·3, 95% UI 5627·7–8482·7), Yemen (4212·4, 3241·3–5418·1), and Egypt (4034·8, 3027·7–5138·6) had the highest age-standardised DALYs rates attributable to air pollution in 2019, whereas Türkiye (1329·2, 1033·7–1654·7), Jordan (1447·3, 1154·2–1758·5), and Iran (1603·0, 1404·7–1813·8) had the lowest rates (appendix pp 4, 10, 16–18). The pattern of age-standardised DALYs rates attributed to PM pollution was similar to that of air pollution. Afghanistan (6955·5, 5597·2–8458·2), Yemen (4178·9, 3212·2–5367·7), and Sudan (3997·6, 3198·1–4995·5) were the three countries in the super-region with the highest age-standardised DALYs rates attributable to household air pollution from solid fuels in 2019 (appendix p 10).

**Figure 1:**
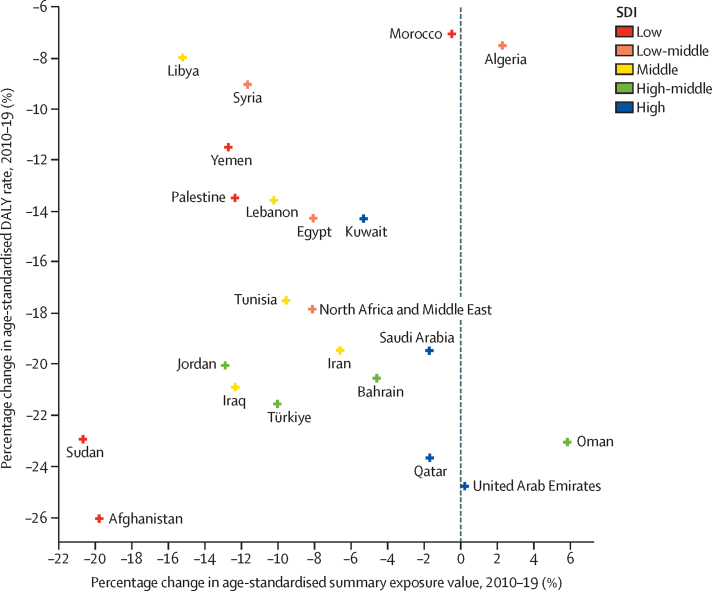
Changes in exposure to air pollution by changes in age-standardised DALYs rate based on the SDI of countries in North Africa and the Middle East, 2010–19 DALY=disability-adjusted life-year. SDI=Socio-demographic Index.

Over the 1990–2019 timeframe, the age-standardised SEV of air pollution decreased by 10·9% (95% UI 5·8–17·7%) in North Africa and the Middle East. Most of the decrease in SEV of air pollution (8·1% [5·6–11·0%]) was witnessed in the 2010–19 period. From 2010 to 2019, the age-standardised SEV for air pollution had a decreasing trend in all countries except for Algeria, Oman, and the UAE. Exposure to PM pollution decreased in all countries except for Algeria, Oman, and the UAE (figure 1). Among the subtypes of PM pollution, exposure to household air pollution from solid fuels decreased by 70·6% (64·1–77·0% (appendix p 11) in the super-region in the past three decades, whereas exposure to ambient PM pollution increased by 40·1% (25·2–63·7%) and exposure to ambient ozone pollution increased by 7·7% (0·7–14·3%). Although the exposure to ambient PM pollution had increased by 40·1% in the 1990–2010 period, it then decreased by 1·4% from 2010 to 2019. Afghanistan (76·7%), Yemen (28·5%), Morocco (19·0%), Oman (7·5%), and Algeria (3·4%) had increased exposure to ambient PM pollution from 2010 to 2019. Exposure to household air pollution from solid fuels decreased in all countries (appendix p 11). There was substantial heterogeneity in the changes in age-standardised DALYs rate and SEVs based on the SDIs of the countries in the super-region. Qatar, the UAE, Saudi Arabia, and Oman were the countries with significant decreases in their age-standardised DALYs rate, despite a less than 5% decrease, constant exposure, or an increase in air pollution since 2010 (figure 1).

**Figure 2:**
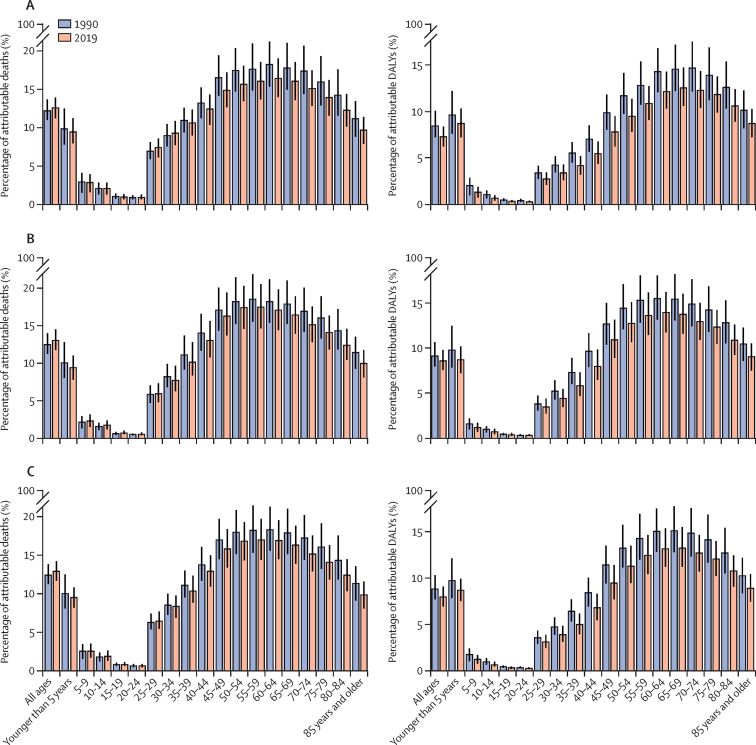
Percentage of deaths and DALYs attributable to air pollution by age groups among women (A), men (B), and both sexes (C) in North Africa and the Middle East, 1990 and 2019 DALYs=disability-adjusted life-years.

When looking at outcomes by age groups, the death rates attributable to air pollution in both 1990 and 2019 and in both males and females were lowest among those aged 5–24 years and increase with age, peaking in those aged 95 years and older (appendix pp 12–13). The lowest proportion of deaths and DALYs were in those aged 5–24 years and the highest in those aged 50–74 years (figure 2). Among the age groups younger than 20 years, the DALYs rates attributable to air pollution decreased with increasing age. From the age group 20–24 years, the DALYs rates attributable to air pollution increased until the 85–89 year age group, after which the rates then decreased. The pattern of DALYs rates attributed to PM pollution was similar to that of air pollution overall (appendix pp 14–15). The percentage of DALYs attributable to air pollution decreased from the younger than 5 years age group to the 20–24 year age group. From the 25–29 year age group, the percentage of DALYs attributable to air pollution increased up to the 65–69 year age group, and then presented a decrease in both 1990 and 2019 (figure 2).

**Figure 3:**
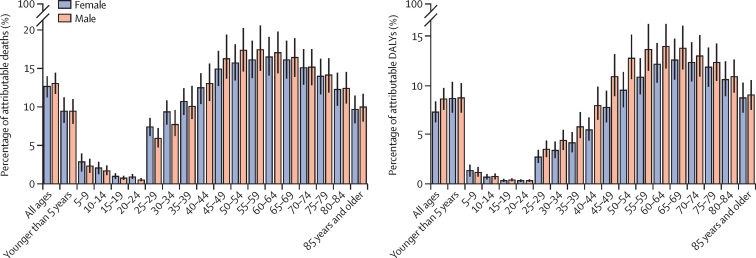
Percentage of deaths and DALYs attributable to air pollution by age groups and by sex in North Africa and the Middle East, 2019 DALYs=disability-adjusted life-years.

The percentage of deaths attributable to air pollution was not substantially different between women and men (figure 3; appendix p 7). Similarly, the percentage of DALYs attributable to air pollution was not substantially different between women and men (figure 3; appendix p 9).

**Figure 4:**
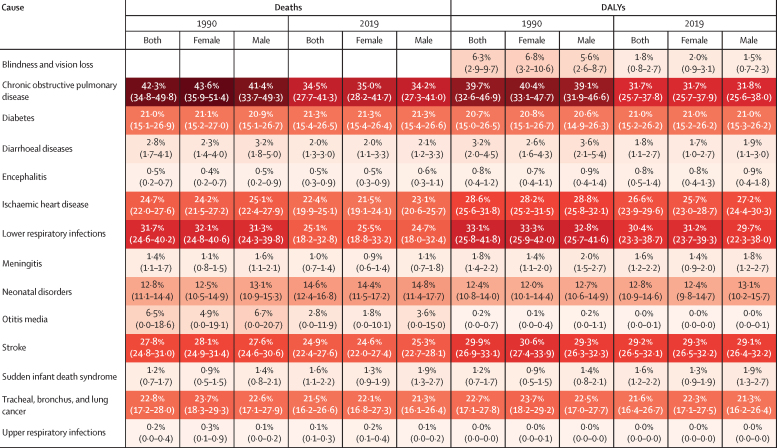
Age-standardised cause-specific deaths and DALYs attributable to air pollution by sex, 1990 and 2019 Data shown as % (95% uncertainty interval). DALYs=disability-adjusted life-years.

For specific causes, 34·5% (95% UI 27·7–41·3%) of deaths for COPD were attributable to air pollution, as were 25·1% (18·2–32·8%) of deaths for lower respiratory infections, 24·9% (22·4–27·6%) for stroke, 22·4% (19·9–25·1%) for ischaemic heart disease, 21·5% (16·2–26·6%) for tracheal, bronchus, and lung cancer, and 21·3% (15·4–26·5%) for diabetes in 2019 (figure 4). 31·7% (95% UI 25·7–37·8%) of DALYs for COPD were attributable to air pollution in 2019, as were 30·4% (23·3–38·7) of DALYs for lower respiratory infections, 29·2% (26·5–32·1%) for stroke, 26·6% (23·9–29·6%) for ischaemic heart disease, 21·6% (16·4–26·7%) for tracheal, bronchus, and lung cancer, and 21·0% (15·2–26·2%) for diabetes (figure 4).

If air pollution had been lowered to the TMRELs for the year 2019, then we estimate that the average life expectancy change in North Africa and the Middle East in 2019 would have been 1·6 years, with the most substantial effect in Afghanistan (2·3 years), Sudan (2·2 years), and Egypt (2·0 years; appendix pp 19–21). If the exposure to PM pollution had been lower than the TMRELs, life expectancy would have increased in the super-region by 1·6 years (appendix p 22), with the most substantial change in Afghanistan (2·3 years), Sudan (2·2 years), and Egypt (2·0 years; appendix p 22). For household air pollution from solid fuels, the life expectancy change in the super-region was 0·2 years, with the most substantial gain in Afghanistan (1·7 years), Sudan (0·8 years), and Yemen (0·8 years; appendix p 23). Changes in life expectancy because of the risk deletion of ambient PM pollution are presented in the appendix (p 24).

## Discussion

In 2019, air pollution accounted for 12·8% of all deaths and 8·0% of all DALYs in North Africa and the Middle East. Nevertheless, the age-standardised death rates attributable to air pollution decreased by 37·6%, and DALYs attributable to air pollution decreased by 44·5%, in the 1990–2019 period.

Countries in the North Africa and the Middle East super-region were among the countries worldwide with the highest death rates attributable to long-term exposure to air pollution in 2015.^22^ Air pollution continues to take a heavy toll in the super-region, both on women and men. Propitiously, exposure to air pollution decreased by 10·9% in the super-region in the past three decades, most of which was witnessed in the 2010–19 period. In 2014, the UN Environment Assembly adopted a resolution to promote good air quality, including ten actions aimed at industrial activities, road transport, open waste burning, indoor air pollution, and general legislative efforts. However, approaches towards air pollution have varied among countries in the super-region. Although there are countries with specific legislation on air quality, some countries in the super-region have not established any air pollution standards.^23^ Understandably, there was substantial heterogeneity among countries in the super-region regarding the burden imposed by air pollution. Afghanistan, Egypt, and Yemen had the highest age-standardised death and DALYs rates attributable to air pollution overall and PM pollution specifically in 2019, while Türkiye, Jordan, and Kuwait had the lowest age-standardised DALYs and death rates.

The North Africa and the Middle East super-region is part of the so-called dust belt, where most dust storms originate.^24^ The frequency and intensity of dust storms have substantially escalated during the past three decades because of climate change, desertification, land degradation, and drought.^25^ This change calls for concerted action plans with the prompt cooperation of all countries in the super-region. Moreover, the effect of more frequent and potent dust storms on the progress made in lessening the air pollution burden needs to be investigated in future studies.

Although natural sources such as windblown dust play a notable role in air pollution in the super-region, the effect of anthropogenic emission sources cannot be ignored. Given the many petroleum refineries and power plants powered by fossil fuels, industrial emissions are considered the super-region's most notable anthropogenic source of air pollution.^23^ In addition, the non-sustainable development and urbanisation trends also fuel air pollution via low-standard construction activities, household power generation, and transportation.^26^ Unlike most countries with a high SDI outside of the North Africa and the Middle East, there has been low investment in public transport among high SDI countries in the super-region.^27^ Nevertheless, there is a growing interest in expanding public transport^28^ and vehicle electrification^29^ in the super-region, especially among countries with a higher SDI.

Exposure to ambient PM pollution has increased in the past three decades, most notably in the 1990–2010 period, and then plateaued until 2019. We estimated that the average life expectancy in North Africa and the Middle East would have been higher by 1·6 years if air pollution had been lowered to the TMRELs for 2019. In this sense, Afghanistan, Sudan, and Egypt would all benefit the most, underscoring the need for prompt interventions.

Exposure to household air pollution from solid fuels substantially decreased in all countries of the super-region in the past three decades. Nevertheless, Afghanistan, Yemen, and Sudan were the countries with the most age-standardised DALYs and death rates attributable to household air pollution from solid fuels in 2019. In other countries, 97% of the population have access to clean non-solid fuels for their domestic energy needs.^23^ Although the proportion of households in the super-region that mainly relied on solid fuels for cooking decreased from 1980 to 2010, the actual number of people exposed slightly increased because of population growth.^30^ This finding calls for governmental action plans to promote sustainable access to non-solid fuels, efficient stoves, and improved household ventilation.^31^ Moreover, the availability of electricity, high education, and high household income have been associated with avoiding solid fuel use.^32^ Prompt mitigation strategies could result in substantial public health benefits, considering that the life expectancy of Sudan, Yemen, and Afghanistan could be 0·8–1·7 years higher should household air pollution be eliminated.

The cause-specific burden attributable to air pollution was substantial. The interaction between air pollution and non-communicable diseases (NCDs) could involve a vicious cycle. On one hand, robust evidence suggests that long-term exposure to air pollution is associated with an increased incidence of COPD,^33^ diabetes,^34^ and ischaemic heart disease.^35^ On the other hand, patients with COPD,^36^ diabetes,^34^ and ischaemic heart disease^37^ could be more susceptible to the adverse effects of air pollution.

Addressing the conspicuous burden of air pollution requires holistic views and a deep understanding of its underlying sources. A range of strategies for sustainable transportation, clean sources of energy for industries and households, and concerted efforts against climate change are needed, emphasising the lessons learned from previous interventions towards eliminating the burden of air pollution among all countries of the super-region. In this sense, there is a clear role for the joint actions of global organisations to encourage regulatory improvements in these countries to address exposure to all types of air pollution.^31^ It is also noteworthy that, despite the favourable trends of some countries in reducing the attributable burden of air pollution in the super-region, there was substantial heterogeneity between these countries. The most notable of these differences were three countries involved in war, political instability, and poverty: Afghanistan, Yemen, and Sudan, where household air pollution still takes a heavy toll. In contrast, the wealthy Arab states of the Persian Gulf, including Qatar, the UAE, and Saudi Arabia, were the countries with substantial decreases in their age-standardised DALYs rate despite a less than 5% decrease or increased exposure to air pollution since 2010. Such observations underscore the devastating effects of political and economic instabilities on the planetary health of societies. Notably, air pollution, climate change, and NCDs are linked to threats to planetary health. Despite their complex interactions, these issues share common origins and joint solutions; nevertheless, they are often dealt with in isolation, and efforts to lessen their burden often opt for different solutions.^38^

This is the first study to present estimates of the effect of air pollution on disease burden, mortality, and life expectancy in the North Africa and the Middle East super-region on the basis of data from GBD 2019. The study allows the investigation of the situation and trends of health metrics and measures in the super-region during the past three decades. TMREL was used in this study to estimate the health risks associated with exposure to environmental pollutants.^14^ This approach is used to represent the uncertainty regarding whether the scientific evidence is consistent with adverse effects of exposure. Nevertheless, the overall quality of the GBD estimates fundamentally relies on the accuracy of the data sources used in the modelling. There are not many studies on risk exposure or outcomes in the super-region, especially in countries undergoing war or unrest. Moreover, the reliability of registry data among countries with few resources in the super-region could be questionable. Thus, the GBD includes various modelling processes to overcome this limitation and presents metrics with 95% UIs. Moreover, the GBD does not provide subnational data estimates for all countries in the super-region and thus makes it unfeasible to investigate within‐country inequities by location and subpopulations. Another limitation was that providing estimates on the effect of ambient ozone pollution on life expectancy was not possible because of insufficient data, which warrants further investigation. The burden attributable to air pollution substantially decreased in the study period. Although exposure to air pollution overall decreased, exposure to ambient PM pollution increased in the past three decades. Most of the decrease in exposure to air pollution was witnessed in the past decade. Exposure to air pollution reduces life expectancy, and we estimated that the average human life in North Africa and the Middle East was shortened by approximately 1·6 years from the combined effect of ambient and household PM air pollution. Despite the favourable trends of countries in reducing the attributable burden of air pollution in the super-region, there was substantial heterogeneity, especially among those involved in internal conflicts and crises. The cause-specific burden attributable to air pollution was notable, especially for NCDs. Dealing with the complex interactions between air pollution, climate change, and NCDs requires joint efforts towards bridging the inequality gaps in the super-region through cooperation and solidarity across countries.

## Data sharing

To download the data used in these analyses, please visit the Global Burden of Disease Results Tool (http://ghdx.healthdata.org/gbd-results-tool), made public by the Institute for Health Metrics and Evaluation.

## Declaration of interests

We declare no competing interests.
