# Supplementary material for: Effect of air pollution on disease burden, mortality, and life expectancy in North Africa and the Middle East: a systematic analysis for the Global Burden of Disease Study 2019
Source: Lancet Planet Health. 2023 May 8;7(5):e358–69. doi: 10.1016/S2542-5196(23)00053-0 (PMC10186179; doi:10.1016/S2542-5196(23)00053-0)
Supplement: Supplementary appendix [file mmc1.pdf]

THE LANCET
Planetary Health

Supplementary appendix

This appendix formed part of the original submission and has been peer reviewed. We post it as supplied by the authors.

Supplement to: Abbasi-Kangevari M, Malekpour M-R, Masinaei M, et al. Effect of air pollution on disease burden, mortality, and life expectancy in North Africa and the Middle East: a systematic analysis for the Global Burden of Disease Study 2019. *Lancet Planet Health* 2023; **7:** e358–69.

Supplementary appendix for "Air pollution impact on disease burden, mortality, and life expectancy in the North Africa and Middle East: A systematic analysis for the Global Burden of Disease Study 2019"

Supplementary Table 1. Data sources of GBD study 2019 for air pollution in North Africa and Middle East ……………………………………………..…………………………………………………. 3
Supplementary Table 2. Age-standardised death and DALYs rates per 100 000 attributable to air pollution among countries in North Africa and Middle East in 1990 and 2019 ………………………………..……… 4
Supplementary Table 3. All-ages deaths attributable to air pollution and its sub-types among countries in North Africa and Middle East in 2019 ……………………………………………………………………………… 5
Supplementary Table 4. Age-standardised death rates per 100 000 attributable to air pollution and its sub-types among countries in North Africa and Middle East in 2019 …………………………………………………..… 6
Supplementary Table 5. The proportion of deaths attributable to air pollution in both sexes by age-groups in North Africa and Middle East in 1990 and 2019 ……………………………………………..……………… 7
Supplementary Table 6. All-ages DALYs attributable to air pollution and its sub-types among countries in North Africa and Middle East in 2019 ……………………………………………………………..……………… 8
Supplementary Table 7. The proportion of DALYs attributable to air pollution in both sexes by age-groups in North Africa and Middle East in 1990 and 2019 ……………………………………………………………..…… 9
Supplementary Table 8. Age-standardised DALYs rates per 100 000 attributable to air pollution and its sub-types among countries in North Africa and Middle East in 2019 ……………………………………..…….. 10
Supplementary Table 9. Exposure to air pollution among countries in North Africa and Middle East in the 1990-2019 and 2010-2019 periods ………………………………………………………..…………….... 11
Supplementary Table 10. Death rates per 100 000 attributable to air pollution and its sub-types among age-groups in countries of North Africa and Middle East in 2019 ……………………….................................. 12
Supplementary Table 11. The number of deaths attributable to air pollution and its sub-types by age-groups among countries in North Africa and Middle East in 2019 ………………………..……………………….... 13
Supplementary Table 12. DALYs rates per 100 000 attributable to air pollution and its sub-types among age-groups in countries of North Africa and Middle East in 2019 ……………………..……………………….... 14
Supplementary Table 13. The number of DALYs attributable to air pollution and its sub-types by age-groups among countries in North Africa and Middle East in 2019 ……………………..………………………….... 15
Supplementary Figure 1. Age-standardised death and DALYs rates per 100 000 attributable to air pollution among women (a), men (b), and both sexes (c) in countries of North Africa and Middle East in 1990 and 2019 ……………………………………………………………………………………..…………………….. 16
Supplementary Figure 2. Changes in life expectancy by age in North Africa and Middle East in 2019 if air pollution had been lowered to the theoretical minimum risk exposure levels among women (a), men (b), and both sexes (c) ………………………………………………………………………………..……… 19

Supplementary Figure 3. Changes in life expectancy by age in North Africa and Middle East in 2019 if PM pollution had been lowered to the theoretical minimum risk exposure levels ……………………………..…... 22
Supplementary Figure 4. Changes in life expectancy by age in North Africa and Middle East in 2019 if household air pollution from solid fuels had been lowered to the theoretical minimum risk exposure levels ……………………………………………………………………………………..……………... 23
Supplementary Figure 5. Changes in life expectancy by age in North Africa and Middle East in 2019 if ambient PM pollution had been lowered to the theoretical minimum risk exposure levels …………………………….. 24

Supplementary Table 1. Data sources of GBD study 2019 for air pollution in North Africa and Middle East

N	Citation
1	National Office of Statistics (Algeria), League of Arab States. Algeria Maternal and Child Health Survey 1992
2	Algeria Multiple Indicator Cluster Survey 2006 as it appears in World Health Organization (WHO). WHO Household Energy Database 1974-2008. Geneva, Switzerland: World Health Organization (WHO), 2010
3	Ministry of Health and Population (Algeria), United Nations Children's Fund (UNICEF). Algeria Multiple Indicator Cluster Survey 2012-2013. New York, United States of America: United Nations Children's Fund (UNICEF), 2018
4	Shaddick G, Thomas ML. Particulate Matter 2.5 and 10 Surface Monitor Station Expanded Database 2008-2017. [Unpublished]
5	Bahrain - Manama AirNow Air Quality Data 2017 as it appears in Shaddick G, Thomas ML. Particulate Matter 2.5 and 10 Surface Monitor Station Expanded Database 2008-2017. [Unpublished]
6	Egypt Annual Air Quality Report 2013 as it appears in Shaddick G, Thomas ML. Particulate Matter 2.5 and 10 Surface Monitor Station Expanded Database 2008-2017. [Unpublished]
7	Central Agency for Public Mobilization and Statistics (CAPMAS) (Egypt), League of Arab States. Egypt Maternal and Child Health Survey 1991
8	Macro International, Inc, Population Council (Egypt). Egypt Demographic and Health Survey 2000. Fairfax, United States of America: ICF International
9	El-Zanaty and Associates, Macro International, Inc, Ministry of Health and Population (Egypt), Population Council (Egypt). Egypt Demographic and Health Survey 2005. Fairfax, United States of America: ICF International
10	El-Zanaty and Associates, Macro International, Inc, Ministry of Health and Population (Egypt), Population Council (Egypt). Egypt Interim Demographic and Health Survey 2003. Fairfax, United States of America: ICF International
11	Central Agency for Public Mobilization and Statistics (CAPMAS) (Egypt), Minnesota Population Center, University of Minnesota. Egypt Population, Housing, and Establishment Census 1996 - IPUMS. Minneapolis, United States of America: University of Minnesota
12	Central Agency for Public Mobilization and Statistics (CAPMAS) (Egypt), Minnesota Population Center, University of Minnesota. Egypt General Census for Population, Housing, and Establishments 2006 - IPUMS. Minneapolis, United States of America: University of Minnesota, 2011
13	Gholampour A, Nabizadeh R, Naseri S, Yunesian M, Taghipour H, Rastkari N, Nazmara S, Faridi S, Mahvi Amir H. Exposure and health impacts of outdoor particulate matter in two urban and industrialized area of Tabriz, Iran. J Environ Health Sci Eng. 2014; 12: 27 as it appears in Shaddick G, Thomas ML. Particulate Matter 2.5 and 10 Surface Monitor Station Expanded Database 2008-2017. [Unpublished]
14	Mohammadyan M, Shabankhani B. Indoor PM 1, PM 2.5, PM 10, and outdoor PM 2.5 concentrations in primary schools in Sari, Iran. Arh Hig Rada Toksikol. 2013; 64(3): 371-7 as it appears in Shaddick G, Thomas ML. Particulate Matter 2.5 and 10 Surface Monitor Station Expanded Database 2008-2017. [Unpublished]
15	The Battle Against Solid UFP-emissions from Internal Combustion Engines Without Waiting for Euro VI as it appears in Shaddick G, Thomas ML. Particulate Matter 2.5 and 10 Surface Monitor Station Expanded Database 2008-2017. [Unpublished]
16	Iran - Tehran Air Quality Control Company Particulate Matter Data 2013 as it appears in Shaddick G, Thomas ML. Particulate Matter 2.5 and 10 Surface Monitor Station Expanded Database 2008-2017. [Unpublished]
17	Iran - Tehran Air Quality Control Company Particulate Matter Data 2010 as it appears in Shaddick G, Thomas ML. Particulate Matter 2.5 and 10 Surface Monitor Station Expanded Database 2008-2017. [Unpublished]
18	Iran - Tehran Air Quality Control Company Particulate Matter Data 2012 as it appears in Shaddick G, Thomas ML. Particulate Matter 2.5 and 10 Surface Monitor Station Expanded Database 2008-2017. [Unpublished]
19	Iran - Tehran Air Quality Control Company Particulate Matter Data 2014 as it appears in Shaddick G, Thomas ML. Particulate Matter 2.5 and 10 Surface Monitor Station Expanded Database 2008-2017. [Unpublished]
20	Iran - Tehran Air Quality Control Company Particulate Matter Data 2015 as it appears in Shaddick G, Thomas ML. Particulate Matter 2.5 and 10 Surface Monitor Station Expanded Database 2008-2017. [Unpublished]
21	Iran - Tehran Air Quality Control Company Particulate Matter Data 2016 as it appears in Shaddick G, Thomas ML. Particulate Matter 2.5 and 10 Surface Monitor Station Expanded Database 2008-2017. [Unpublished]
22	Iran Air Quality Monitoring System Particulate Matter Data 2010 as it appears in Shaddick G, Thomas ML. Particulate Matter 2.5 and 10 Surface Monitor Station Expanded Database 2008-2017. [Unpublished]
23	Iran Air Quality Monitoring System Particulate Matter Data 2012 as it appears in Shaddick G, Thomas ML. Particulate Matter 2.5 and 10 Surface Monitor Station Expanded Database 2008-2017. [Unpublished]
24	Iran Air Quality Monitoring System Particulate Matter Data 2013 as it appears in Shaddick G, Thomas ML. Particulate Matter 2.5 and 10 Surface Monitor Station Expanded Database 2008-2017. [Unpublished]
25	Iran Air Quality Monitoring System Particulate Matter Data 2014 as it appears in Shaddick G, Thomas ML. Particulate Matter 2.5 and 10 Surface Monitor Station Expanded Database 2008-2017. [Unpublished]
26	Statistical Centre of Iran, Minnesota Population Center. Iran General Census of Population and Housing 2006 from the Integrated Public Use Microdata Series, International: Version 6.1 [Machine-readable database]. Minneapolis: University of Minnesota, 2011
27	Hassan MK, Al-Sadoon I. Risk factors for severe pneumonia in children in Basrah. Trop Doct. 2001; 139-41
28	Iraq PM2.5 and PM10 Data 2015 as it appears in Shaddick G, Thomas ML. Particulate Matter 2.5 and 10 Surface Monitor Station Expanded Database 2008-2017. [Unpublished]
29	Iraq PM2.5 and PM10 Data 2016 as it appears in Shaddick G, Thomas ML. Particulate Matter 2.5 and 10 Surface Monitor Station Expanded Database 2008-2017. [Unpublished]
30	United Nations Children's Fund (UNICEF), Central Organization for Statistics and Information Technology (Iraq), Kurdistan Regional Statistics Office. Iraq Multiple Indicator Cluster Survey 2006. New York, United States: United Nations Children's Fund (UNICEF)
31	Central Organization for Statistics and Information Technology (Iraq), The Fafo Research Foundation. Iraq Multiple Indicator Rapid Assessment 2004
32	Central Organization for Statistics and Information Technology (Iraq), Kurdistan Regional Statistics Office, World Bank. Iraq Household Socioeconomic Survey 2006-2007. Washington DC, United States of America: World Bank
33	Central Organization for Statistics and Information Technology (Iraq), Kurdistan Regional Statistics Office, Ministry of Health (Iraq), United Nations Children's Fund (UNICEF). Iraq Multiple Indicator Cluster Survey 2011. New York, United States of America: United Nations Children's Fund (UNICEF), 2013
34	Central Organization for Statistics and Information Technology (Iraq), Kurdistan Regional Statistics Office, World Bank. Iraq Household Socioeconomic Survey 2012-2013. Washington DC, United States of America: World Bank
35	Jordan Ground Station Particulate Matter Data 2015 as it appears in Shaddick G, Thomas ML. Particulate Matter 2.5 and 10 Surface Monitor Station Expanded Database 2008-2017. [Unpublished]
36	Jordan Ground Station Particulate Matter Data 2016 as it appears in Shaddick G, Thomas ML. Particulate Matter 2.5 and 10 Surface Monitor Station Expanded Database 2008-2017. [Unpublished]
37	Jordan Ground Station Particulate Matter Data 2017 as it appears in Shaddick G, Thomas ML. Particulate Matter 2.5 and 10 Surface Monitor Station Expanded Database 2008-2017. [Unpublished]
38	Department of Statistics (Jordan), Macro International, Inc. Jordan Demographic and Health Survey 2002. Fairfax, United States of America: ICF International
39	Department of Statistics (Jordan), Macro International, Inc. Jordan Demographic and Health Survey 2007. Fairfax, United States of America: ICF International
40	Department of Statistics (Jordan), ICF Macro. Jordan Interim Demographic and Health Survey 2009. Fairfax, United States of America: ICF International, 2010
41	Department of Statistics (Jordan), ICF International. Jordan Demographic and Health Survey 2012. Fairfax, United States of America: ICF International
42	Kuwait Ground Station Monitor Particulate Matter Data 2014 as it appears in Shaddick G, Thomas ML. Particulate Matter 2.5 and 10 Surface Monitor Station Expanded Database 2008-2017. [Unpublished]
43	Kuwait Environmental Monitoring Information System Air Quality Data 2015 as it appears in Shaddick G, Thomas ML. Particulate Matter 2.5 and 10 Surface Monitor Station Expanded Database 2008-2017. [Unpublished]
44	Kuwait Environmental Monitoring Information System Air Quality Data 2016 as it appears in Shaddick G, Thomas ML. Particulate Matter 2.5 and 10 Surface Monitor Station Expanded Database 2008-2017. [Unpublished]
45	Kuwait - Kuwait City AirNow Air Quality Data 2017 as it appears in Shaddick G, Thomas ML. Particulate Matter 2.5 and 10 Surface Monitor Station Expanded Database 2008-2017. [Unpublished]
46	Lebanon National Air Quality Monitoring Network PM2.5 Data 2014 as it appears in Shaddick G, Thomas ML. Particulate Matter 2.5 and 10 Surface Monitor Station Expanded Database 2008-2017. [Unpublished]
47	Ministry of Public Health (Lebanon), League of Arab States. Lebanon Maternal and Child Health Survey 1996
48	Lebanon National Survey of Household Living Conditions 2004
49	Committee for Health and Social Insurance (Libya), League of Arab States. Libya Maternal and Child Health Survey 1995
50	Sasco AJ, Merrill RM, Dari I, Benhaïm-Luzon V, Carriot F, Cann CI, Bartal M. A case-control study of lung cancer in Casablanca, Morocco. Cancer Causes Control. 2002; 13(7): 609–16
51	Bouh HA, Benyaich F, Bounakhla M, Noack Y, Tahri M, Zahry F. Seasonal variations of the atmospheric particles and its chemical components in Meknes City - Morocco. J Mater Environ. 2013; 4(1): 49-62 as it appears in Shaddick G, Thomas ML. Particulate Matter 2.5 and 10 Surface Monitor Station Expanded Database 2008-2017. [Unpublished]
52	Morocco Ground Station Particulate Matter Data 2013 as it appears in Shaddick G, Thomas ML. Particulate Matter 2.5 and 10 Surface Monitor Station Expanded Database 2008-2017. [Unpublished]
53	Morocco Ground Station Particulate Matter Data 2011 as it appears in Shaddick G, Thomas ML. Particulate Matter 2.5 and 10 Surface Monitor Station Expanded Database 2008-2017. [Unpublished]
54	Morocco Ground Station Particulate Matter Data 2012 as it appears in Shaddick G, Thomas ML. Particulate Matter 2.5 and 10 Surface Monitor Station Expanded Database 2008-2017. [Unpublished]
55	Morocco PM2.5 and PM10 Data 2016 as it appears in Shaddick G, Thomas ML. Particulate Matter 2.5 and 10 Surface Monitor Station Expanded Database 2008-2017. [Unpublished]
56	Ministry of Health (Morocco), League of Arab States. Morocco Maternal and Child Health Survey 1996-1997
57	League of Arab States, Macro International, Inc, Ministry of Health (Morocco). Morocco Demographic and Health Survey 2003-2004. Fairfax, United States of America: ICF International
58	World Health Organization (WHO). Morocco World Health Survey 2003. Geneva, Switzerland: World Health Organization (WHO), 2005
59	High Commission for Planning (Morocco), World Bank. Morocco Household and Youth Survey 2009-2010
60	Palestinian Central Bureau of Statistics, Minnesota Population Center. Palestine Population, Housing, and Establishment Census 1997 from the Integrated Public Use Microdata Series, International: [Machine-readable database]. Minneapolis: University of Minnesota
61	Palestinian Central Bureau of Statistics, Minnesota Population Center. Palestine Population, Housing, and Establishment Census 2007 from the Integrated Public Use Microdata Series, International: [Machine-readable database]. Minneapolis: University of Minnesota, 2011
62	Ministry of Health (Palestine), Palestinian Central Bureau of Statistics, United Nations Children's Fund (UNICEF), United Nations Population Fund (UNFPA). Palestine Multiple Indicator Cluster Survey 2010. New York, United States of America: United Nations Children's Fund (UNICEF), 2014
63	Palestinian Central Bureau of Statistics. Palestine Household Energy Survey, January 2005
64	Ministry of Health (Palestine), Palestinian Central Bureau of Statistics, United Nations Children's Fund (UNICEF). Palestine Multiple Indicator Cluster Survey 2014. New York, United States of America: United Nations Children's Fund (UNICEF), 2015
65	Oman Ground Station Monitor Particulate Matter Data 2009 as it appears in Shaddick G, Thomas ML. Particulate Matter 2.5 and 10 Surface Monitor Station Expanded Database 2008-2017. [Unpublished]
66	Review of Environmental Exposures to Carcinogens in Qatar as it appears in Shaddick G, Thomas ML. Particulate Matter 2.5 and 10 Surface Monitor Station Expanded Database 2008-2017. [Unpublished]
67	Qatar Ground Monitor Station Particulate Matter Data 2012 as it appears in Shaddick G, Thomas ML. Particulate Matter 2.5 and 10 Surface Monitor Station Expanded Database 2008-2017. [Unpublished]
68	Qatar Statistics Authority. Qatar Population and Housing Census 2010
69	General Secretariat for Development Planning (Qatar), Qatar Statistics Authority. Qatar Population Status Report 2012. 2012
70	Saudi Arabia PM2.5 and PM10 Data 2014 as it appears in Shaddick G, Thomas ML. Particulate Matter 2.5 and 10 Surface Monitor Station Expanded Database 2008-2017. [Unpublished]
71	Saudi Arabia PM2.5 and PM10 Data 2015 as it appears in Shaddick G, Thomas ML. Particulate Matter 2.5 and 10 Surface Monitor Station Expanded Database 2008-2017. [Unpublished]
72	Saudi Arabia PM2.5 and PM10 Data 2016 as it appears in Shaddick G, Thomas ML. Particulate Matter 2.5 and 10 Surface Monitor Station Expanded Database 2008-2017. [Unpublished]
73	Saudi Arabia PM2.5 and PM10 Data 2017 as it appears in Shaddick G, Thomas ML. Particulate Matter 2.5 and 10 Surface Monitor Station Expanded Database 2008-2017. [Unpublished]
74	Central Bureau of Statistics (Syria), League of Arab States. Syria Maternal and Child Health Survey 1993
75	United Nations Children's Fund (UNICEF), Central Bureau of Statistics (Syria), Ministry of Health (Syria), Pan Arab Project for Family Health (PAPFAM). Syria Multiple Indicator Cluster Survey 2006. New York, United States: United Nations Children's Fund (UNICEF)
76	Bouchlaghem K, Nsom B. Effect of atmospheric pollutants on the air quality in Tunisia. Sci World J. 2012; 2012: 1-8 as it appears in Shaddick G, Thomas ML. Particulate Matter 2.5 and 10 Surface Monitor Station Expanded Database 2008-2017. [Unpublished]
77	World Health Organization (WHO). Tunisia World Health Survey 2003. Geneva, Switzerland: World Health Organization (WHO), 2005
78	Tunisia Multiple Indicator Cluster Survey 2006 as it appears in World Health Organization (WHO). WHO Household Energy Database 1974-2008. Geneva, Switzerland: World Health Organization (WHO), 2010
79	Ministry of Regional Development and Planning (Tunisia), National Institute of Statistics (Tunisia), United Nations Children's Fund (UNICEF). Tunisia Multiple Indicator Cluster Survey 2011-2012. New York, United States of America: United Nations Children's Fund (UNICEF), 2014
80	Sezer H, Akkurt I, Guler N, Marakoğlu K, Berk S. A case-control study on the effect of exposure to different substances on the development of COPD. Ann Epidemiol. 2006; 16(1): 59–62
81	Airbase European Air Quality Database Version 8, 2014 as it appears in Shaddick G, Thomas ML. Particulate Matter 2.5 and 10 Surface Monitor Station Expanded Database 2008-2017. [Unpublished]
82	EEA Air Quality e-Reporting Database 2017 as it appears in Shaddick G, Thomas ML. Particulate Matter 2.5 and 10 Surface Monitor Station Expanded Database 2008-2017. [Unpublished]
83	EEA Air Quality e-Reporting Database 2018 as it appears in Shaddick G, Thomas ML. Particulate Matter 2.5 and 10 Surface Monitor Station Expanded Database 2008-2017. [Unpublished]
84	United Arab Emirates - Abu Dhabi Air Quality Monitoring System 2011 as it appears in Shaddick G, Thomas ML. Particulate Matter 2.5 and 10 Surface Monitor Station Expanded Database 2008-2017. [Unpublished]
85	United Arab Emirates - Abu Dhabi Air Quality Monitoring System 2012 as it appears in Shaddick G, Thomas ML. Particulate Matter 2.5 and 10 Surface Monitor Station Expanded Database 2008-2017. [Unpublished]
86	United Arab Emirates - Abu Dhabi Air Quality Monitoring System 2013 as it appears in Shaddick G, Thomas ML. Particulate Matter 2.5 and 10 Surface Monitor Station Expanded Database 2008-2017. [Unpublished]
87	United Arab Emirates - Abu Dhabi Air Quality Monitoring System 2014 as it appears in Shaddick G, Thomas ML. Particulate Matter 2.5 and 10 Surface Monitor Station Expanded Database 2008-2017. [Unpublished]
88	United Arab Emirates - Abu Dhabi Air Quality Monitoring System 2015 as it appears in Shaddick G, Thomas ML. Particulate Matter 2.5 and 10 Surface Monitor Station Expanded Database 2008-2017. [Unpublished]
89	World Health Organization (WHO). United Arab Emirates World Health Survey 2003. Geneva, Switzerland: World Health Organization (WHO), 2005
90	Al-Sonboli N, Hart CA, Al-Aghbari N, Al-Ansi A, Ashoor O, Cuevas LE. Human metapneumovirus and respiratory syncytial virus disease in children, Yemen. Emerg Infect Dis. 2006; 12(9): 1437–9
91	Ministry of Health (Yemen) and United Nations Children's Fund (UNICEF). Yemen Multiple Indicator Cluster Survey 2006. New York, United States: United Nations Children's Fund (UNICEF)
92	Central Statistical Organization (Yemen), League of Arab States, Macro International, Inc. Yemen Demographic and Health Survey 1991-1992. Fairfax, United States of America: ICF International
93	Yemen Household Budget Survey 1998 as it appears in World Health Organization (WHO). WHO Household Energy Database 1974-2008. Geneva, Switzerland: World Health Organization (WHO), 2010
94	Central Statistical Organization (Yemen), ICF International, Ministry of Public Health and Population (Yemen). Yemen Demographic and Health Survey 2013. Fairfax, United States of America: ICF International
95	Magnusson R, Hägglund L, Wingfors H. Broad exposure screening of air pollutants in the occupational environment of Swedish soldiers deployed in Afghanistan. Mil Med. 2012; 177(3): 318-25 as it appears in Shaddick G, Thomas ML. Particulate Matter 2.5 and 10 Surface Monitor Station Expanded Database 2008-2017. [Unpublished]
96	Central Statistics Organization (Afghanistan), European Union (EU), Ministry of Rural Rehabilitation and Development (Afghanistan). Afghanistan National Risk and Vulnerability Assessment 2005
97	Indian Institute of Health Management Research (IIHMR), Johns Hopkins University, Ministry of Public Health (Afghanistan). Afghanistan Health Survey 2006
98	Central Statistics Organization (Afghanistan), ICF Macro, Indian Institute of Health Management Research (IIHMR), Ministry of Public Health (Afghanistan), World Health Organization Regional Office for the Eastern Mediterranean (EMRO-WHO). Afghanistan Special Demographic and Health Survey 2010. Fairfax, United States of America: ICF International
99	Central Statistics Organization (Afghanistan), United Nations Children's Fund (UNICEF). Afghanistan Multiple Indicator Cluster Survey 2010-2011. New York, United States of America: United Nations Children's Fund (UNICEF), 2013
100	Central Statistics Organization (Afghanistan), ICF International, Ministry of Public Health (Afghanistan). Afghanistan Demographic and Health Survey 2015-2016. Fairfax, United States of America: ICF International, 2017
101	Central Statistics Organization (Afghanistan), ICON-INSTITUTE Consulting Group. Afghanistan Living Conditions Survey 2013-2014
102	Central Bureau of Statistics (Sudan), Federal Ministry of Health (Sudan), United Nations Children's Fund (UNICEF). Sudan Multiple Indicator Cluster Survey 2000. New York, United States of America: United Nations Children's Fund (UNICEF)
103	Federal Ministry of Health (Sudan), League of Arab States. Sudan Maternal and Child Health Survey 1992-1993
104	Ministry of Health (Southern Sudan), Federal Ministry of Health (Sudan), Southern Sudan Centre for Census, Statistics and Evaluation (SSCCSE), Central Bureau of Statistics (Sudan). Sudan Family Health Survey 2006
105	National Population Census Council (Sudan), Central Bureau of Statistics (Sudan), Southern Sudan Centre for Census, Statistics and Evaluation (SSCCSE), Minnesota Population Center. Sudan Population and Housing Census 2008 from the Integrated Public Use Microdata Series, International: [Machine-readable database]. Minneapolis: University of Minnesota, 2011
106	Federal Ministry of Health and Central Bureau of Statistics, Sudan Household and Health Survey - 2, 2012, National report. Khartoum, Republic of Sudan: Federal Ministry of Health and Central Bureau of Statistics
107	Central Bureau of Statistics (Sudan), Ministry of Health (South Sudan). Sudan - North Multiple Indicator Cluster Survey 2010. New York, United States of America: United Nations Children's Fund (UNICEF), 2015
108	Central Bureau of Statistics (Sudan), Federal Ministry of Health (Sudan), United Nations Children's Fund (UNICEF). Sudan Multiple Indicator Cluster Survey 2014. New York, United States of America: United Nations Children's Fund (UNICEF), 2016

Supplementary Table 2. Age-standardised death and DALYs rates per 100 000 attributable to air pollution among countries in North Africa and Middle East in 1990 and 2019

	Deaths		DALYs	
	1990	2019	1990	2019
NAME	158.3 (139.1-177.8)	98.8 (84.7-114.7)	4,884.2 (4,381.5-5,555.4)	2,710.4 (2,317.3-3,125.6)
Afghanistan	402.2 (324.1-504.7)	238.3 (189.8-290.1)	14,310.9 (11,460.9-17,782.8)	6,992.3 (5,627.7-8,482.7)
Algeria	133.9 (100.2-172.7)	78.8 (56.2-103.6)	3,242.4 (2,403.1-4,157.2)	1,873.1 (1,360.6-2,452.1)
Bahrain	218.5 (185.3-252.7)	107.4 (86.6-132.7)	4,891.5 (4,166.2-5,611.1)	2,267.4 (1,840.5-2,768.8)
Egypt	200.5 (172.3-233.7)	159.7 (119.2-203.0)	6,289.6 (5,349.3-7,314.4)	4,034.8 (3,027.7-5,138.6)
Iran	116.4 (102.5-131.3)	65.5 (56.5-74.0)	3,198.7 (2,826.7-3,625.6)	1,603.0 (1,404.7-1,813.8)
Iraq	172.9 (140.1-207.8)	123.0 (95.5-150.6)	5,043.6 (4,205.0-5,964.9)	3,088.2 (2,399.6-3,837.4)
Jordan	101.2 (82.6-122.5)	56.3 (45.0-69.7)	2,559.9 (2,075.2-3,070.1)	1,447.3 (1,154.2-1,758.5)
Kuwait	93.0 (81.1-105.0)	65.0 (53.0-79.4)	2,481.6 (2,193.9-2,821.6)	1,709.5 (1,411.1-2,058.8)
Lebanon	95.6 (74.6-119.4)	65.5 (50.5-80.9)	2,490.4 (1,947.2-3,062.8)	1,674.6 (1,282.0-2,049.6)
Libya	90.8 (69.6-114.6)	71.9 (51.2-97.7)	2,665.1 (2,117.5-3,297.4)	1,962.9 (1,425.0-2,584.8)
Morocco	127.2 (100.9-153.7)	105.5 (80.8-129.8)	3,825.8 (3,110.4-4,601.2)	2,617.0 (2,024.3-3,246.4)
Oman	214.0 (162.4-266.8)	129.4 (102.4-157.3)	5,145.6 (3,953.9-6,493.2)	2,667.0 (2,118.3-3,232.2)
Palestine	139.0 (105.0-176.3)	92.2 (69.9-113.8)	3,401.6 (2,622.2-4,301.2)	2,119.4 (1,627.0-2,587.6)
Qatar	195.9 (162.5-232.6)	131.2 (105.4-163.0)	4,383.0 (3,689.1-5,189.6)	2,540.2 (2,039.5-3,125.5)
Saudi Arabia	156.3 (123.2-190.2)	112.0 (91.1-134.6)	4,003.5 (3,204.4-4,870.8)	2,831.1 (2,310.6-3,421.2)
Sudan	293.9 (241.7-365.0)	149.3 (117.9-189.6)	9,410.3 (7,782.7-11,582.0)	4,022.9 (3,219.5-5,027.8)
Syria	133.4 (103.5-167.3)	101.4 (75.0-135.0)	3,710.2 (2,895.6-4,591.0)	2,434.6 (1,792.4-3,233.0)
UAE	172.1 (137.4-213.8)	103.8 (77.7-134.3)	3,826.5 (3,073.0-4,772.5)	2,491.6 (1,889.3-3,211.0)
Tunisia	89.9 (69.4-112.5)	65.2 (44.9-88.9)	2,434.3 (1,881.6-3,044.3)	1,608.3 (1,166.6-2,133.4)
Turkey	102.4 (86.5-120.7)	53.3 (40.8-68.5)	3,156.3 (2,587.5-3,803.4)	1,329.2 (1,033.7-1,654.7)
Yemen	283.0 (218.4-368.8)	154.0 (116.3-202.0)	8,823.6 (7,018.5-11,116.2)	4,212.4 (3,241.3-5,418.1)

Supplementary Table 3. All-ages deaths attributable to air pollution and its sub-types among countries in North Africa and Middle East in 2019

	Air pollution	Particulate matter pollution	Ambient particulate matter pollution	Household air pollution from solid fuels	Ambient ozone pollution
NAME	398,559 (339,434-463,723)	391,042 (332,909-454,990)	339,456 (284,221-396,047)	51,586 (35,572-70,524)	10,315 (4,691-16,290)
Afghanistan	37,033 (29,774-45,042)	36,847 (29,560-44,803)	8,679 (4,029-16,057)	28,168 (19,479-37,650)	475 (214-786)
Algeria	22,162 (15,819-29,631)	21,680 (15,341-28,991)	21,613 (15,285-28,926)	66 (25-138)	616 (283-1,037)
Bahrain	638 (508-800)	625 (495-785)	624 (494-783)	2 (0-4)	19 (9-32)
Egypt	91,663 (67,756-118,198)	90,632 (66,870-116,905)	90,559 (66,819-116,869)	73 (26-170)	1,610 (681-2,741)
Iran	43,203 (37,478-48,717)	41,839 (36,244-47,155)	41,742 (36,156-47,037)	97 (38-217)	1,794 (842-2,766)
Iraq	25,626 (19,579-31,849)	25,442 (19,417-31,662)	25,378 (19,358-31,598)	64 (22-136)	259 (122-412)
Jordan	3,131 (2,499-3,881)	3,075 (2,455-3,822)	3,074 (2,453-3,820)	2 (0-4)	71 (34-114)
Kuwait	1,543 (1,275-1,897)	1,527 (1,262-1,868)	1,526 (1,260-1,868)	1 (0-2)	25 (11-41)
Lebanon	3,382 (2,614-4,203)	3,312 (2,528-4,128)	3,303 (2,517-4,107)	10 (3-23)	86 (35-150)
Libya	3,445 (2,441-4,701)	3,375 (2,390-4,620)	3,368 (2,381-4,613)	8 (2-19)	92 (41-157)
Morocco	29,249 (22,074-36,163)	28,743 (21,626-35,760)	27,063 (20,245-34,050)	1,679 (733-3,317)	665 (293-1,105)
Oman	1,576 (1,241-1,913)	1,559 (1,225-1,899)	1,553 (1,216-1,894)	6 (2-16)	23 (10-37)
Palestine	1,848 (1,401-2,278)	1,820 (1,383-2,251)	1,792 (1,362-2,219)	28 (13-57)	35 (16-57)
Qatar	545 (416-717)	539 (411-709)	539 (411-709)	0 (0-0)	9 (5-16)
Saudi Arabia	18,036 (14,220-22,351)	17,832 (14,055-22,170)	17,795 (13,993-22,106)	37 (9-100)	310 (140-491)
Sudan	28,163 (22,325-35,706)	27,944 (22,149-35,432)	16,634 (10,204-24,378)	11,310 (6,569-17,281)	372 (153-667)
Syria	10,671 (7,723-14,480)	10,491 (7,606-14,188)	10,474 (7,596-14,159)	18 (6-41)	227 (99-393)
UAE	3,361 (2,360-4,656)	3,252 (2,259-4,490)	3,252 (2,259-4,490)	0 (0-0)	149 (59-264)
Tunisia	7,530 (5,194-10,288)	7,358 (5,088-10,135)	7,337 (5,059-10,121)	21 (7-50)	216 (94-361)
Turkey	44,166 (33,650-56,769)	41,760 (31,571-53,456)	41,524 (31,395-53,238)	236 (73-595)	2,933 (1,229-5,046)
Yemen	21,184 (16,008-27,529)	20,991 (15,846-27,214)	11,282 (5,681-17,408)	9,709 (5,596-14,628)	316 (138-547)

Supplementary Table 4. Age-standardised death rates per 100 000 attributable to air pollution and its sub-types among countries in North Africa and Middle East in 20192019

	Air pollution	Particulate matter pollution	Ambient particulate matter pollution	Household air pollution from solid fuels	Ambient ozone pollution
NAME	98.8 (84.7-114.7)	96.6 (82.6-111.9)	85.2 (72.0-98.8)	11.5 (7.8-15.7)	3.0 (1.4-4.7)
Afghanistan	238.3 (189.8-290.1)	236.4 (187.9-288.4)	56.9 (26.3-103.1)	179.5 (123.3-242.1)	5.0 (2.2-8.4)
Algeria	78.8 (56.2-103.6)	76.8 (54.5-101.8)	76.5 (54.2-101.6)	0.2 (0.1-0.5)	2.6 (1.2-4.3)
Bahrain	107.4 (86.6-132.7)	104.0 (83.6-128.5)	103.7 (83.4-128.2)	0.3 (0.1-0.7)	5.0 (2.3-8.5)
Egypt	159.7 (119.2-203.0)	157.6 (117.6-200.2)	157.5 (117.5-200.1)	0.1 (0.0-0.3)	3.3 (1.4-5.5)
Iran	65.5 (56.5-74.0)	63.3 (54.7-71.5)	63.1 (54.5-71.4)	0.1 (0.1-0.3)	2.9 (1.4-4.5)
Iraq	123.0 (95.5-150.6)	121.9 (94.6-149.5)	121.6 (94.4-149.3)	0.3 (0.1-0.7)	1.5 (0.7-2.4)
Jordan	56.3 (45.0-69.7)	55.0 (44.0-68.3)	55.0 (43.9-68.3)	0.0 (0.0-0.1)	1.6 (0.7-2.5)
Kuwait	65.0 (53.0-79.4)	64.1 (52.5-78.4)	64.1 (52.4-78.4)	0.0 (0.0-0.1)	1.4 (0.6-2.3)
Lebanon	65.5 (50.5-80.9)	64.1 (49.1-79.7)	63.9 (48.9-79.1)	0.2 (0.1-0.4)	1.7 (0.7-3.0)
Libya	71.9 (51.2-97.7)	70.3 (50.0-96.0)	70.1 (49.9-95.8)	0.2 (0.0-0.4)	2.1 (1.0-3.6)
Morocco	105.5 (80.8-129.8)	103.5 (79.0-126.9)	97.4 (73.4-121.5)	6.1 (2.7-12.0)	2.7 (1.2-4.4)
Oman	129.4 (102.4-157.3)	127.5 (100.4-155.7)	127.0 (100.2-154.6)	0.5 (0.1-1.3)	2.6 (1.1-4.3)
Palestine	92.2 (69.9-113.8)	90.6 (68.7-112.5)	89.1 (67.0-110.3)	1.4 (0.6-2.8)	2.0 (1.0-3.3)
Qatar	131.2 (105.4-163.0)	128.9 (103.1-159.5)	128.9 (103.1-159.5)	0.0 (0.0-0.0)	3.9 (2.0-6.3)
Saudi Arabia	112.0 (91.1-134.6)	110.1 (89.5-132.7)	109.8 (89.3-132.5)	0.2 (0.1-0.6)	2.9 (1.3-4.6)
Sudan	149.3 (117.9-189.6)	147.9 (116.0-188.0)	88.7 (54.4-130.9)	59.1 (34.8-90.7)	2.5 (1.0-4.5)
Syria	101.4 (75.0-135.0)	99.4 (73.5-131.9)	99.3 (73.3-131.8)	0.2 (0.1-0.4)	2.5 (1.1-4.3)
UAE	103.8 (77.7-134.3)	100.7 (74.2-130.6)	100.7 (74.2-130.6)	0.0 (0.0-0.0)	4.3 (2.0-7.0)
Tunisia	65.2 (44.9-88.9)	63.6 (44.0-87.4)	63.4 (43.8-87.2)	0.2 (0.1-0.4)	2.0 (0.9-3.3)
Turkey	53.3 (40.8-68.5)	50.4 (38.2-64.1)	50.1 (38.0-63.9)	0.3 (0.1-0.7)	3.6 (1.5-6.2)
Yemen	154.0 (116.3-202.0)	152.1 (114.6-199.3)	82.5 (42.3-128.2)	69.6 (39.4-106.3)	3.1 (1.3-5.3)

Supplementary Table 5. The proportion of deaths attributable to air pollution in both sexes by age-groups in North Africa and Middle East in 1990 and 2019

	1990			2019		
	Both	Female	Male	Both	Female	Male
Under 5	9.9% (8.1-12.4)	9.8% (7.8-12.5)	10.0% (8.0-12.8)	9.4% (8.1-10.8)	9.5% (7.9-11.3)	9.4% (7.8-11.0)
5 to 9	2.5% (1.5-3.4)	2.9% (1.5-4.1)	2.1% (1.3-2.9)	2.5% (1.7-3.5)	2.8% (1.6-4.0)	2.2% (1.4-3.2)
10 to 14	1.7% (1.2-2.3)	2.1% (1.2-2.8)	1.5% (1.0-2.0)	1.8% (1.3-2.5)	2.0% (1.3-2.8)	1.7% (1.1-2.4)
15 to 19	0.7% (0.5-1.0)	1.0% (0.7-1.4)	0.6% (0.4-0.8)	0.8% (0.6-1.1)	0.9% (0.6-1.3)	0.7% (0.5-1.0)
20 to 24	0.6% (0.5-0.8)	0.9% (0.6-1.2)	0.4% (0.3-0.6)	0.6% (0.4-0.8)	0.9% (0.6-1.2)	0.5% (0.3-0.7)
25 to 29	6.3% (5.3-7.4)	6.9% (5.9-8.1)	5.8% (4.7-7.0)	6.4% (5.4-7.6)	7.4% (6.2-8.6)	5.9% (4.7-7.3)
30 to 34	8.5% (7.2-9.9)	9.0% (7.6-10.5)	8.1% (6.8-9.9)	8.3% (6.8-9.7)	9.3% (7.7-10.9)	7.7% (6.2-9.6)
35 to 39	11.0% (9.4-12.9)	10.9% (9.4-12.6)	11.1% (9.2-13.6)	10.3% (8.8-12.3)	10.7% (9.0-12.4)	10.1% (8.4-12.7)
40 to 44	13.7% (11.6-16.0)	13.2% (11.3-15.3)	13.9% (11.6-16.6)	12.8% (10.9-15.0)	12.5% (10.4-14.3)	13.0% (10.8-15.6)
45 to 49	16.9% (14.5-19.6)	16.6% (14.2-19.4)	17.1% (14.3-20.1)	15.7% (13.4-18.3)	14.9% (12.7-17.2)	16.3% (13.6-19.4)
50 to 54	17.9% (15.1-20.8)	17.5% (14.8-20.4)	18.2% (15.2-21.4)	16.7% (14.3-19.2)	15.7% (13.5-18.1)	17.3% (14.6-20.2)
55 to 59	18.2% (15.3-21.4)	17.7% (14.9-21.1)	18.5% (15.5-21.9)	16.9% (14.3-19.6)	16.1% (13.7-18.6)	17.5% (14.6-20.6)
60 to 64	18.2% (15.5-21.3)	18.3% (15.4-21.3)	18.2% (15.4-21.2)	16.8% (14.4-19.4)	16.5% (14.1-19.1)	17.0% (14.4-19.8)
65 to 69	17.8% (15.1-21.0)	17.8% (15.0-21.1)	17.8% (15.2-21.0)	16.3% (13.8-18.7)	16.1% (13.7-18.6)	16.4% (13.9-18.9)
70 to 74	17.1% (14.3-20.2)	17.4% (14.4-20.7)	16.9% (14.1-20.1)	15.1% (12.9-17.5)	15.1% (12.8-17.5)	15.1% (12.8-17.5)
75 to 79	16.0% (13.2-19.1)	16.0% (12.9-19.4)	16.0% (13.3-18.9)	14.0% (11.8-16.2)	14.0% (11.6-16.3)	14.1% (11.8-16.3)
80 to 84	14.3% (11.8-17.5)	14.3% (11.6-17.7)	14.3% (11.8-17.2)	12.3% (10.3-14.5)	12.3% (10.1-14.4)	12.4% (10.4-14.5)
85 to 89	12.3% (9.9-14.8)	12.2% (9.6-14.9)	12.4% (10.1-14.8)	10.6% (8.6-12.6)	10.5% (8.5-12.6)	10.8% (8.7-12.7)
90 to 94	10.2% (7.8-12.5)	10.1% (7.6-12.6)	10.2% (7.9-12.5)	8.9% (6.9-10.9)	8.7% (6.7-10.6)	9.1% (7.2-11.1)
95 plus	8.1% (5.9-10.2)	8.0% (5.7-10.2)	8.2% (6.2-10.2)	7.2% (5.4-8.7)	7.0% (5.1-8.5)	7.4% (5.7-8.9)
All Ages	12.3% (11.2-13.8)	12.2% (11.0-13.7)	12.4% (11.2-14.0)	12.8% (11.6-14.2)	12.6% (11.3-14.0)	13.0% (11.7-14.5)
Age-standardised	13.8% (12.3-15.4)	13.7% (12.1-15.4)	13.9% (12.5-15.5)	12.8% (11.4-14.1)	12.5% (11.0-13.9)	13.0% (11.6-14.4)

Supplementary Table 6. All-ages DALYs attributable to air pollution and its sub-types among countries in North Africa and Middle East in 2019

	Air pollution	Particulate matter pollution	Ambient particulate matter pollution	Household air pollution from solid fuels	Ambient ozone pollution
NAME	13,056,864 (11,145,813-15,128,048)	12,913,831 (11,041,330-14,981,099)	10,569,826 (8,866,135-12,416,213)	2,344,005 (1,617,635-3,125,700)	199,665 (91,412-316,069)
Afghanistan	1,849,170 (1,485,560-2,250,664)	1,844,663 (1,482,722-2,247,858)	421,330 (194,229-771,057)	1,423,333 (989,316-1,896,253)	11,529 (5,064-19,295)
Algeria	654,753 (474,698-855,730)	646,191 (465,841-847,016)	644,038 (460,511-844,371)	2,153 (855-4,494)	10,933 (5,004-18,307)
Bahrain	22,129 (17,716-27,370)	21,883 (17,437-27,108)	21,817 (17,401-27,007)	66 (22-152)	369 (168-617)
Egypt	2,852,145 (2,127,564-3,667,673)	2,829,087 (2,110,966-3,643,778)	2,826,672 (2,109,892-3,642,261)	2,415 (961-5,462)	36,026 (15,053-61,660)
Iran	1,190,330 (1,042,443-1,349,000)	1,166,154 (1,018,765-1,322,534)	1,162,989 (1,017,542-1,318,067)	3,165 (1,313-6,900)	31,852 (15,156-48,998)
Iraq	812,427 (633,004-1,019,981)	808,672 (630,365-1,016,097)	806,497 (629,066-1,014,743)	2,175 (789-4,611)	5,287 (2,473-8,387)
Jordan	107,365 (85,574-131,797)	106,253 (84,622-130,424)	106,197 (84,582-130,378)	55 (19-125)	1,402 (666-2,251)
Kuwait	55,184 (45,487-67,036)	54,914 (45,248-66,653)	54,886 (45,212-66,619)	28 (9-71)	409 (180-694)
Lebanon	87,694 (67,136-107,256)	86,562 (66,041-106,271)	86,279 (65,847-105,967)	283 (95-650)	1,409 (582-2,496)
Libya	108,846 (78,263-144,897)	107,493 (77,197-143,228)	107,207 (76,991-142,992)	286 (94-671)	1,794 (793-3,078)
Morocco	826,112 (633,361-1,040,719)	816,389 (627,015-1,029,923)	766,926 (582,464-971,836)	49,463 (22,059-97,060)	12,782 (5,627-21,253)
Oman	50,843 (40,109-62,250)	50,516 (39,770-61,952)	50,276 (39,536-61,513)	241 (79-552)	450 (202-721)
Palestine	55,374 (43,554-67,487)	54,830 (43,129-66,991)	53,927 (42,041-66,027)	903 (417-1,776)	690 (316-1,110)
Qatar	25,039 (19,801-31,509)	24,894 (19,677-31,250)	24,892 (19,674-31,248)	2 (1-5)	237 (116-402)
Saudi Arabia	659,239 (519,196-823,068)	654,330 (515,275-818,298)	652,668 (511,795-816,660)	1,662 (549-4,068)	7,441 (3,410-11,960)
Sudan	1,058,647 (816,291-1,332,226)	1,054,163 (811,316-1,326,078)	612,394 (364,525-895,741)	441,769 (250,451-670,568)	7,639 (3,107-14,158)
Syria	305,020 (221,150-410,574)	301,341 (219,268-405,364)	300,810 (218,805-404,543)	531 (196-1,213)	4,644 (2,030-7,977)
UAE	147,250 (107,970-198,734)	143,110 (103,881-193,166)	143,103 (103,876-193,162)	7 (2-19)	5,680 (2,093-10,186)
Tunisia	197,299 (141,925-265,118)	194,357 (139,430-261,703)	193,749 (138,935-261,100)	608 (223-1,389)	3,697 (1,601-6,268)
Turkey	1,121,784 (872,449-1,412,431)	1,082,245 (838,547-1,356,307)	1,075,598 (833,084-1,352,670)	6,647 (2,275-16,111)	48,187 (21,045-81,553)
Yemen	856,948 (657,364-1,110,810)	852,665 (654,532-1,104,671)	446,833 (227,070-680,407)	405,832 (233,140-612,315)	7,005 (3,009-12,205)

Supplementary Table 7. The proportion of DALYs attributable to air pollution in both sexes by age-groups in North Africa and Middle East in 1990 and 2019

	1990			2019		
	Both	Female	Male	Both	Female	Male
Under 5	9.7% (7.9-12.1)	9.6% (7.6-12.2)	9.8% (7.8-12.5)	8.7% (7.5-9.9)	8.7% (7.2-10.3)	8.7% (7.2-10.2)
5 to 9	1.8% (1.0-2.4)	2.0% (1.0-2.8)	1.6% (1.0-2.2)	1.2% (0.8-1.7)	1.3% (0.7-1.9)	1.1% (0.7-1.7)
10 to 14	1.0% (0.7-1.3)	1.0% (0.6-1.5)	0.9% (0.6-1.3)	0.7% (0.4-1.0)	0.7% (0.4-1.0)	0.7% (0.4-1.0)
15 to 19	0.4% (0.3-0.6)	0.5% (0.3-0.7)	0.4% (0.3-0.5)	0.3% (0.2-0.5)	0.3% (0.2-0.5)	0.4% (0.2-0.5)
20 to 24	0.3% (0.2-0.5)	0.4% (0.3-0.6)	0.3% (0.2-0.4)	0.3% (0.2-0.4)	0.3% (0.2-0.4)	0.3% (0.2-0.4)
25 to 29	3.6% (2.9-4.4)	3.4% (2.7-4.2)	3.8% (3.1-4.7)	3.1% (2.4-3.9)	2.7% (2.1-3.4)	3.5% (2.7-4.4)
30 to 34	4.7% (3.9-5.8)	4.3% (3.4-5.2)	5.2% (4.2-6.4)	3.9% (3.1-4.9)	3.4% (2.6-4.3)	4.4% (3.4-5.5)
35 to 39	6.4% (5.3-7.7)	5.5% (4.5-6.7)	7.3% (6.0-8.9)	5.0% (3.9-6.1)	4.2% (3.2-5.2)	5.8% (4.6-7.3)
40 to 44	8.4% (6.9-10.1)	7.0% (5.7-8.5)	9.6% (7.9-11.7)	6.8% (5.5-8.3)	5.5% (4.4-6.7)	8.0% (6.5-9.9)
45 to 49	11.4% (9.4-13.5)	9.9% (8.1-11.8)	12.7% (10.4-15.1)	9.5% (7.7-11.4)	7.8% (6.3-9.5)	10.9% (8.9-13.2)
50 to 54	13.2% (11.2-15.7)	11.7% (9.8-14.2)	14.4% (12.1-17.1)	11.3% (9.4-13.5)	9.5% (7.8-11.4)	12.8% (10.7-15.1)
55 to 59	14.3% (12.0-16.9)	12.8% (10.7-15.4)	15.3% (12.9-18.1)	12.4% (10.4-14.7)	10.8% (9.1-12.8)	13.7% (11.5-16.2)
60 to 64	15.0% (12.8-17.5)	14.3% (12.1-16.9)	15.6% (13.1-18.1)	13.2% (11.2-15.4)	12.2% (10.3-14.3)	13.9% (11.7-16.3)
65 to 69	15.1% (12.8-17.8)	14.5% (12.2-17.2)	15.5% (13.1-18.3)	13.2% (11.2-15.5)	12.6% (10.6-14.7)	13.8% (11.6-16.0)
70 to 74	14.8% (12.3-17.6)	14.7% (12.2-17.6)	14.9% (12.4-17.7)	12.7% (10.7-14.7)	12.3% (10.4-14.4)	13.0% (10.9-15.1)
75 to 79	14.1% (11.6-16.8)	13.9% (11.3-16.9)	14.3% (11.8-16.9)	12.1% (10.2-14.0)	11.8% (9.9-13.8)	12.3% (10.5-14.3)
80 to 84	12.7% (10.5-15.4)	12.6% (10.3-15.4)	12.8% (10.6-15.3)	10.7% (9.1-12.5)	10.6% (8.9-12.4)	10.9% (9.3-12.6)
85 to 89	10.9% (9.0-13.1)	10.8% (8.7-13.2)	11.1% (9.2-13.2)	9.4% (7.8-11.2)	9.3% (7.7-11.0)	9.6% (7.9-11.3)
90 to 94	9.1% (7.1-11.1)	9.0% (6.9-11.1)	9.2% (7.3-11.1)	8.0% (6.3-9.6)	7.7% (6.0-9.3)	8.2% (6.6-9.9)
95 plus	7.3% (5.5-9.1)	7.3% (5.4-9.1)	7.4% (5.7-9.1)	6.5% (5.0-7.8)	6.3% (4.7-7.6)	6.7% (5.2-8.1)
All Ages	8.8% (7.7-10.3)	8.5% (7.2-10.1)	9.1% (7.9-10.7)	8.0% (7.0-9.1)	7.3% (6.2-8.4)	8.6% (7.5-9.8)
Age-standardised	9.9% (8.8-11.2)	9.4% (8.3-10.8)	10.4% (9.3-11.7)	8.7% (7.6-9.9)	8.0% (6.9-9.1)	9.3% (8.3-10.5)

Supplementary Table 8. Age-standardised DALYs rates per 100 000 attributable to air pollution and its sub-types among countries in North Africa and Middle East in 2019

	Air pollution	Particulate matter pollution	Ambient particulate matter pollution	Household air pollution from solid fuels	Ambient ozone pollution
NAME	2,710.4 (2,317.3-3,125.6)	2,674.5 (2,290.5-3,087.7)	2,233.6 (1,874.6-2,613.0)	440.9 (305.1-591.1)	49.6 (22.6-78.7)
Afghanistan	6,992.3 (5,627.7-8,482.7)	6,955.5 (5,597.2-8,458.2)	1,627.2 (743.8-2,980.8)	5,328.4 (3,722.0-7,108.4)	94.0 (41.5-155.1)
Algeria	1,873.1 (1,360.6-2,452.1)	1,844.1 (1,334.3-2,411.5)	1,837.9 (1,330.3-2,405.8)	6.2 (2.5-12.6)	37.0 (17.0-62.5)
Bahrain	2,267.4 (1,840.5-2,768.8)	2,223.6 (1,796.5-2,717.6)	2,216.4 (1,791.3-2,702.8)	7.1 (2.5-16.4)	65.8 (30.4-110.1)
Egypt	4,034.8 (3,027.7-5,138.6)	3,996.7 (3,007.2-5,109.1)	3,993.2 (3,004.7-5,103.7)	3.5 (1.4-7.8)	59.6 (25.2-101.6)
Iran	1,603.0 (1,404.7-1,813.8)	1,568.1 (1,369.9-1,772.3)	1,563.8 (1,364.6-1,767.5)	4.3 (1.8-9.3)	46.0 (21.9-70.8)
Iraq	3,088.2 (2,399.6-3,837.4)	3,070.4 (2,384.4-3,820.8)	3,062.1 (2,378.8-3,811.0)	8.3 (3.1-17.4)	25.1 (11.9-39.9)
Jordan	1,447.3 (1,154.2-1,758.5)	1,427.7 (1,140.3-1,734.6)	1,427.0 (1,139.9-1,733.9)	0.8 (0.3-1.7)	24.7 (11.7-39.7)
Kuwait	1,709.5 (1,411.1-2,058.8)	1,696.3 (1,399.6-2,048.1)	1,695.4 (1,399.2-2,047.4)	1.0 (0.3-2.3)	20.0 (8.8-33.2)
Lebanon	1,674.6 (1,282.0-2,049.6)	1,652.9 (1,262.5-2,029.7)	1,647.5 (1,255.6-2,026.4)	5.4 (1.8-12.4)	27.1 (11.1-47.9)
Libya	1,962.9 (1,425.0-2,584.8)	1,935.3 (1,408.5-2,553.4)	1,929.9 (1,402.4-2,546.0)	5.3 (1.8-12.3)	36.6 (16.2-62.6)
Morocco	2,617.0 (2,024.3-3,246.4)	2,583.1 (2,001.0-3,211.7)	2,425.7 (1,865.8-3,037.8)	157.5 (70.7-308.3)	44.5 (19.4-74.1)
Oman	2,667.0 (2,118.3-3,232.2)	2,639.2 (2,083.9-3,199.4)	2,626.0 (2,077.9-3,186.2)	13.2 (4.5-29.9)	38.2 (16.6-61.2)
Palestine	2,119.4 (1,627.0-2,587.6)	2,093.1 (1,609.8-2,552.7)	2,058.4 (1,566.3-2,521.4)	34.8 (16.4-68.1)	33.3 (15.4-53.9)
Qatar	2,540.2 (2,039.5-3,125.5)	2,510.4 (2,015.5-3,082.6)	2,510.2 (2,015.2-3,082.3)	0.2 (0.1-0.6)	48.6 (24.2-81.1)
Saudi Arabia	2,831.1 (2,310.6-3,421.2)	2,800.0 (2,284.8-3,390.3)	2,792.0 (2,278.4-3,383.9)	8.0 (2.8-18.3)	47.2 (21.4-75.3)
Sudan	4,022.9 (3,219.5-5,027.8)	3,997.6 (3,198.1-4,995.5)	2,354.2 (1,440.2-3,438.2)	1,643.5 (968.3-2,465.3)	43.0 (17.7-77.9)
Syria	2,434.6 (1,792.4-3,233.0)	2,401.5 (1,768.8-3,194.9)	2,397.2 (1,766.9-3,192.3)	4.3 (1.6-9.7)	41.7 (18.0-72.0)
UAE	2,491.6 (1,889.3-3,211.0)	2,430.2 (1,835.7-3,130.9)	2,430.1 (1,835.6-3,130.9)	0.1 (0.0-0.4)	84.2 (37.8-141.9)
Tunisia	1,608.3 (1,166.6-2,133.4)	1,583.6 (1,142.0-2,104.7)	1,578.6 (1,139.4-2,099.6)	5.0 (1.8-11.4)	31.0 (13.4-52.2)
Turkey	1,329.2 (1,033.7-1,654.7)	1,282.6 (997.4-1,598.2)	1,274.6 (990.7-1,593.0)	8.0 (2.7-19.3)	56.9 (24.6-96.5)
Yemen	4,212.4 (3,241.3-5,418.1)	4,178.9 (3,212.2-5,367.7)	2,229.6 (1,140.1-3,425.1)	1,949.3 (1,126.6-2,891.7)	54.9 (23.7-94.9)

Supplementary Table 9. Exposure to air pollution among countries in North Africa and Middle East in the 1990-2019 and 2010-2019 periods

		Air pollution	Particulate matter pollution	Ambient particulate matter pollution	Household air pollution from solid fuels	Ambient ozone pollution
NAME	1990-2019	-10.9% (-17.7 to -5.8)	-10.9% (-18.0 to -5.7)	40.1% (25.2 to 63.7)	-70.6% (-77.0 to -64.1)	7.7% (0.7 to 14.3)
	2010-2019	-8.1% (-11.0 to -5.6)	-8.3% (-11.2 to -5.7)	-1.4% (-4.1 to 1.3)	-37.0% (-44.3 to -29.8)	8.2% (-0.8 to 14.9)
Afghanistan	1990-2019	-27.1% (-40.6 to -15.9)	-28.3% (-42.7 to -16.9)	178.5% (48.3 to 583.9)	-46.6% (-57.7 to -33.2)	-3.0% (-15.8 to 25.3)
	2010-2019	-19.8% (-28.4 to -12.4)	-20.4% (-30.3 to -12.5)	76.7% (33.7 to 152.5)	-34.9% (-43.4 to -26.8)	2.8% (-22.3 to 12.4)
Algeria	1990-2019	-2.2% (-12.6 to 5.0)	-2.4% (-12.8 to 4.9)	30.9% (15.2 to 63.7)	-98.7% (-99.5 to -96.9)	10.2% (-22.0 to 32.2)
	2010-2019	2.3% (0.6 to 3.9)	2.2% (0.5 to 3.8)	3.4% (1.6 to 5.1)	-74.3% (-83.3 to -62.6)	4.3% (-22.5 to 17.3)
Bahrain	1990-2019	-5.2% (-16.7 to 7.0)	-5.7% (-17.1 to 6.5)	6.1% (-9.2 to 24.7)	-97.0% (-98.8 to -92.9)	10.9% (-21.1 to 117.7)
	2010-2019	-4.6% (-8.2 to -1.2)	-5.0% (-8.6 to -1.9)	-4.9% (-8.6 to -1.4)	-60.3% (-74.8 to -38.9)	9.7% (-20.6 to 102.1)
Egypt	1990-2019	-3.5% (-8.3 to -0.1)	-3.3% (-8.1 to 0.1)	19.6% (7.7 to 42.8)	-99.5% (-99.8 to -98.7)	-5.3% (-19.4 to 27.4)
	2010-2019	-8.1% (-11.6 to -5.0)	-8.0% (-11.5 to -4.9)	-8.8% (-12.6 to -5.3)	-75.3% (-84.3 to -62.3)	-3.5% (-14.7 to 29.5)
Iran	1990-2019	-7.0% (-12.9 to -2.7)	-7.2% (-13.2 to -2.9)	18.4% (7.9 to 36.8)	-98.8% (-99.5 to -97.0)	4.6% (-5.0 to 23.2)
	2010-2019	-6.6% (-9.3 to -3.8)	-7.0% (-9.8 to -4.2)	-6.6% (-9.3 to -3.6)	-70.3% (-81.2 to -55.7)	15.5% (1.0 to 36.1)
Iraq	1990-2019	-17.8% (-26.6 to -11.3)	-17.8% (-26.6 to -11.2)	28.4% (4.9 to 78.0)	-99.3% (-99.7 to -98.4)	0.1% (-20.1 to 9.8)
	2010-2019	-12.3% (-16.3 to -8.4)	-12.4% (-16.4 to -8.4)	-11.0% (-15.3 to -6.2)	-88.4% (-92.9 to -81.8)	-0.2% (-19.1 to 5.1)
Jordan	1990-2019	5.2% (-0.4 to 11.6)	5.3% (-0.4 to 11.9)	9.9% (3.5 to 17.5)	-98.5% (-99.4 to -96.2)	-1.3% (-20.1 to 26.1)
	2010-2019	-12.9% (-18.9 to -6.8)	-12.9% (-18.9 to -6.7)	-13.4% (-19.5 to -6.9)	-59.7% (-75.1 to -38.9)	-2.1% (-21.7 to 25.9)
Kuwait	1990-2019	0.2% (-3.5 to 4.5)	0.0% (-3.5 to 4.3)	2.8% (-1.9 to 8.4)	-97.3% (-99.0 to -94.0)	0.3% (-20.2 to 35.9)
	2010-2019	-5.3% (-7.5 to -3.2)	-5.5% (-7.7 to -3.4)	-5.7% (-8.1 to -3.3)	-59.5% (-75.3 to -37.4)	0.3% (-19.5 to 27.9)
Lebanon	1990-2019	-1.3% (-11.4 to 8.2)	-0.9% (-10.9 to 8.7)	30.7% (14.4 to 57.9)	-98.6% (-99.5 to -96.6)	-11.1% (-39.0 to 30.5)
	2010-2019	-10.2% (-15.8 to -5.1)	-10.0% (-15.7 to -5.0)	-9.4% (-15.2 to -4.0)	-74.4% (-84.8 to -62.0)	-11.1% (-41.4 to 30.6)
Libya	1990-2019	-15.0% (-28.7 to -5.3)	-15.1% (-29.0 to -5.4)	35.6% (15.5 to 77.4)	-99.4% (-99.8 to -98.5)	10.3% (-23.5 to 32.3)
	2010-2019	-15.2% (-22.1 to -7.8)	-15.6% (-22.5 to -8.2)	-16.2% (-23.1 to -8.4)	-60.2% (-74.6 to -35.9)	22.2% (-21.1 to 54.7)
Morocco	1990-2019	-10.6% (-26.2 to 4.6)	-10.7% (-26.6 to 4.2)	138.2% (66.2 to 312.0)	-92.5% (-96.4 to -85.6)	92.1% (11.1 to 164.8)
	2010-2019	-0.5% (-4.9 to 3.0)	-0.8% (-5.2 to 2.8)	19.0% (10.9 to 33.4)	-70.0% (-80.7 to -54.9)	30.7% (-20.4 to 67.8)
Oman	1990-2019	-22.8% (-37.9 to -10.7)	-22.7% (-37.6 to -10.8)	77.9% (33.3 to 188.1)	-99.5% (-99.8 to -98.5)	12.2% (-23.9 to 33.6)
	2010-2019	5.8% (0.3 to 11.8)	5.7% (0.1 to 11.7)	7.5% (1.6 to 13.8)	-72.2% (-83.5 to -57.2)	5.0% (-25.2 to 23.0)
Palestine	1990-2019	-18.8% (-32.9 to -7.3)	-18.3% (-32.2 to -6.9)	93.7% (33.8 to 273.6)	-97.9% (-99.1 to -95.0)	-3.5% (-21.9 to 25.2)
	2010-2019	-12.4% (-16.6 to -8.1)	-12.4% (-16.7 to -8.1)	-10.1% (-15.0 to -5.0)	-63.5% (-76.7 to -42.6)	-2.4% (-17.7 to 26.5)
Qatar	1990-2019	-1.2% (-8.0 to 6.2)	-1.7% (-8.3 to 5.6)	-1.9% (-9.3 to 6.8)	-98.2% (-99.4 to -95.4)	0.0% (-20.4 to 48.5)
	2010-2019	-1.7% (-3.1 to -0.4)	-2.1% (-3.5 to -1.0)	-2.4% (-3.8 to -1.2)	-62.7% (-78.4 to -44.1)	-0.7% (-20.2 to 38.2)
Saudi Arabia	1990-2019	-9.8% (-24.0 to 4.4)	-9.7% (-23.8 to 4.4)	118.4% (50.2 to 305.8)	-99.7% (-99.9 to -98.9)	7.9% (-20.2 to 39.6)
	2010-2019	-1.7% (-4.2 to 0.7)	-2.0% (-4.4 to 0.4)	-1.0% (-3.9 to 1.7)	-77.5% (-87.4 to -64.2)	3.8% (-19.2 to 24.0)
Sudan	1990-2019	-43.0% (-57.0 to -28.0)	-43.1% (-57.9 to -27.6)	354.6% (133.7 to 971.9)	-78.7% (-87.2 to -67.8)	74.6% (-0.7 to 107.7)
	2010-2019	-20.7% (-32.2 to -10.6)	-20.7% (-32.1 to -10.6)	61.1% (32.9 to 116.4)	-54.9% (-66.0 to -42.7)	75.0% (-0.3 to 109.0)
Syria	1990-2019	-9.2% (-18.1 to 0.4)	-9.0% (-18.0 to 0.6)	10.8% (-4.8 to 36.4)	-99.0% (-99.6 to -97.3)	-4.1% (-16.4 to 26.1)
	2010-2019	-11.7% (-16.8 to -6.9)	-11.6% (-16.7 to -6.8)	-11.6% (-16.8 to -6.4)	-63.6% (-76.8 to -46.1)	-3.8% (-16.2 to 26.5)
UAE	1990-2019	-0.3% (-5.9 to 4.5)	-0.2% (-5.9 to 4.5)	0.4% (-5.6 to 5.4)	-99.2% (-99.8 to -98.0)	-0.9% (-11.6 to 24.9)
	2010-2019	0.2% (-2.3 to 2.6)	0.3% (-2.2 to 2.7)	0.3% (-2.3 to 2.8)	-46.4% (-66.4 to -19.0)	-1.2% (-12.1 to 24.6)
Tunisia	1990-2019	-9.9% (-19.9 to -3.3)	-9.8% (-19.7 to -3.1)	25.8% (10.5 to 54.9)	-98.9% (-99.6 to -97.4)	-3.1% (-14.2 to 29.0)
	2010-2019	-9.6% (-14.1 to -5.3)	-9.8% (-14.5 to -5.6)	-9.5% (-14.1 to -5.1)	-68.7% (-79.6 to -54.1)	8.5% (-24.1 to 30.9)
Turkey	1990-2019	-14.3% (-25.4 to -5.6)	-14.3% (-25.3 to -5.5)	20.4% (5.6 to 47.8)	-98.1% (-99.3 to -95.2)	-4.3% (-11.7 to 37.5)
	2010-2019	-10.0% (-15.9 to -4.5)	-10.2% (-16.1 to -4.6)	-9.2% (-15.5 to -3.4)	-68.1% (-80.7 to -49.4)	2.8% (-3.4 to 10.7)
Yemen	1990-2019	-42.5% (-59.1 to -27.4)	-42.6% (-60.4 to -27.0)	296.8% (100.3 to 875.8)	-74.7% (-84.0 to -64.3)	72.4% (-0.8 to 117.4)
	2010-2019	-12.7% (-22.6 to -5.7)	-12.8% (-22.7 to -5.9)	28.5% (13.0 to 57.6)	-36.4% (-51.1 to -21.3)	55.4% (-9.7 to 91.5)

Supplementary Table 10. Death rates per 100 000 attributable to air pollution and its sub-types among age-groups in countries of North Africa and Middle East in 2019

	Air pollution	Particulate matter pollution	Ambient particulate matter pollution	Household air pollution from solid fuels	Ambient ozone pollution
Under 5	47.3 (38.3-58.0)	47.3 (38.3-58.0)	26.1 (19.4-33.9)	21.2 (14.0-29.0)	
5 to 9	1.1 (0.7-1.6)	1.1 (0.7-1.6)	0.7 (0.4-1.0)	0.4 (0.2-0.7)	
10 to 14	0.7 (0.5-1.0)	0.7 (0.5-1.0)	0.5 (0.3-0.7)	0.2 (0.1-0.4)	
15 to 19	0.6 (0.4-0.8)	0.6 (0.4-0.8)	0.4 (0.3-0.6)	0.2 (0.1-0.2)	
20 to 24	0.6 (0.4-0.9)	0.6 (0.4-0.9)	0.4 (0.3-0.7)	0.2 (0.1-0.3)	
25 to 29	6.9 (5.3-8.9)	6.8 (5.3-8.8)	5.6 (4.4-7.2)	1.2 (0.7-1.8)	0.1 (0.0-0.1)
30 to 34	10.3 (7.9-13.1)	10.2 (7.9-13.0)	8.6 (6.6-11.1)	1.6 (1.0-2.4)	0.1 (0.1-0.2)
35 to 39	16.6 (12.9-21.1)	16.4 (12.8-20.9)	14.0 (10.9-18.1)	2.4 (1.5-3.7)	0.2 (0.1-0.3)
40 to 44	29.9 (23.4-37.8)	29.6 (23.2-37.6)	25.1 (19.7-32.1)	4.5 (2.9-6.7)	0.4 (0.2-0.6)
45 to 49	58.8 (46.2-73.0)	58.3 (45.9-72.4)	48.3 (37.9-60.4)	10.0 (6.4-14.7)	0.7 (0.3-1.1)
50 to 54	100.0 (79.0-123.8)	99.0 (78.3-123.0)	84.9 (66.2-106.6)	14.1 (9.2-20.4)	1.4 (0.6-2.2)
55 to 59	156.3 (124.0-193.5)	154.5 (122.5-192.0)	138.7 (110.3-171.8)	15.7 (10.2-22.8)	2.6 (1.2-4.2)
60 to 64	249.0 (198.5-304.1)	245.3 (196.4-300.2)	224.5 (178.8-276.0)	20.8 (13.4-30.2)	5.3 (2.4-8.5)
65 to 69	372.8 (302.3-445.8)	366.1 (295.0-437.9)	332.4 (267.0-401.7)	33.7 (22.0-48.0)	9.4 (4.3-14.9)
70 to 74	544.5 (447.8-647.9)	532.4 (436.7-634.0)	477.6 (386.7-578.6)	54.7 (35.4-78.0)	16.7 (7.7-26.9)
75 to 79	818.2 (674.6-968.0)	797.0 (653.5-944.4)	716.0 (583.9-855.8)	81.1 (52.8-116.6)	28.9 (13.6-46.2)
80 to 84	1,120.0 (927.3-1,320.1)	1,085.0 (892.6-1,280.4)	984.1 (810.9-1,172.1)	101.0 (65.4-146.3)	47.0 (21.7-75.5)
85 to 89	1,572.6 (1,270.5-1,882.6)	1,512.9 (1,221.9-1,821.6)	1,398.1 (1,115.1-1,690.7)	114.8 (71.7-171.0)	79.0 (37.0-126.1)
90 to 94	1,863.0 (1,470.6-2,256.2)	1,780.3 (1,397.7-2,171.7)	1,647.2 (1,286.6-2,027.1)	133.0 (80.7-199.3)	108.9 (49.8-177.3)
95 plus	2,179.0 (1,621.6-2,670.9)	2,070.9 (1,518.4-2,552.5)	1,905.7 (1,395.5-2,339.3)	165.3 (96.0-248.6)	142.2 (63.4-231.0)
All Ages	65.5 (55.8-76.2)	64.2 (54.7-74.7)	55.8 (46.7-65.1)	8.5 (5.8-11.6)	1.7 (0.8-2.7)

Supplementary Table 11. The number of deaths attributable to air pollution and its sub-types by age-groups among countries in North Africa and Middle East in 2019

	Air pollution	Particulate matter pollution	Ambient particulate matter pollution	Household air pollution from solid fuels	Ambient ozone pollution
Under 5	28,238 (22,877-34,632)	28,238 (22,877-34,632)	15,575 (11,572-20,265)	12,664 (8,384-17,339)	
5 to 9	660 (438-930)	660 (438-930)	398 (255-596)	262 (137-421)	
10 to 14	414 (283-580)	414 (283-580)	281 (185-414)	133 (72-211)	
15 to 19	296 (201-436)	296 (201-436)	216 (138-326)	80 (43-132)	
20 to 24	315 (209-458)	315 (209-458)	232 (151-337)	83 (42-137)	
25 to 29	3,585 (2,786-4,619)	3,557 (2,767-4,587)	2,937 (2,267-3,775)	621 (383-955)	43 (19-69)
30 to 34	5,432 (4,197-6,944)	5,385 (4,153-6,870)	4,554 (3,488-5,874)	831 (511-1,279)	70 (32-112)
35 to 39	8,111 (6,335-10,323)	8,043 (6,282-10,260)	6,854 (5,339-8,846)	1,189 (758-1,795)	99 (45-157)
40 to 44	12,287 (9,627-15,558)	12,187 (9,552-15,477)	10,321 (8,082-13,201)	1,866 (1,208-2,740)	145 (66-235)
45 to 49	19,956 (15,669-24,771)	19,797 (15,565-24,560)	16,394 (12,850-20,486)	3,403 (2,164-4,975)	236 (104-378)
50 to 54	27,851 (22,008-34,482)	27,588 (21,803-34,272)	23,663 (18,430-29,697)	3,925 (2,562-5,692)	383 (172-619)
55 to 59	34,597 (27,448-42,824)	34,192 (27,121-42,502)	30,707 (24,403-38,018)	3,484 (2,256-5,052)	576 (265-929)
60 to 64	42,862 (34,156-52,331)	42,214 (33,797-51,671)	38,641 (30,775-47,495)	3,573 (2,305-5,192)	907 (418-1,457)
65 to 69	46,570 (37,755-55,681)	45,727 (36,845-54,702)	41,515 (33,344-50,178)	4,212 (2,746-5,997)	1,175 (532-1,859)
70 to 74	45,743 (37,614-54,424)	44,723 (36,682-53,257)	40,124 (32,489-48,609)	4,599 (2,972-6,553)	1,403 (649-2,261)
75 to 79	43,790 (36,106-51,808)	42,657 (34,977-50,542)	38,317 (31,248-45,803)	4,339 (2,828-6,242)	1,548 (726-2,473)
80 to 84	37,809 (31,303-44,564)	36,628 (30,132-43,225)	33,220 (27,373-39,568)	3,408 (2,209-4,938)	1,586 (734-2,548)
85 to 89	26,612 (21,500-31,859)	25,601 (20,678-30,826)	23,659 (18,870-28,611)	1,943 (1,214-2,894)	1,337 (626-2,135)
90 to 94	10,412 (8,219-12,610)	9,950 (7,812-12,138)	9,207 (7,191-11,330)	744 (451-1,114)	609 (278-991)
95 plus	3,019 (2,247-3,701)	2,870 (2,104-3,537)	2,641 (1,934-3,241)	229 (133-344)	197 (88-320)
All Ages	398,559 (339,434-463,723)	391,042 (332,909-454,990)	339,456 (284,221-396,047)	51,586 (35,572-70,524)	10,315 (4,691-16,290)

Supplementary Table 12. DALYs rates per 100 000 attributable to air pollution and its sub-types among age-groups in countries of North Africa and Middle East in 2019

	Air pollution	Particulate matter pollution	Ambient particulate matter pollution	Household air pollution from solid fuels	Ambient ozone pollution
Under 5	4,193.8 (3,399.3-5,138.5)	4,193.8 (3,399.3-5,138.5)	2,315.2 (1,721.3-3,011.7)	1,878.6 (1,244.3-2,572.9)	
5 to 9	92.9 (61.8-131.0)	92.9 (61.8-131.0)	56.3 (36.4-83.8)	36.6 (19.2-59.0)	
10 to 14	57.5 (39.3-80.6)	57.5 (39.3-80.6)	39.2 (26.0-57.2)	18.3 (10.0-29.1)	
15 to 19	41.3 (28.2-60.5)	41.3 (28.2-60.5)	30.2 (19.4-44.7)	11.1 (5.9-18.2)	
20 to 24	41.7 (28.0-60.3)	41.7 (28.0-60.3)	30.7 (20.0-44.5)	11.0 (5.7-18.0)	
25 to 29	506.5 (405.8-631.3)	503.2 (403.2-628.0)	418.0 (332.8-520.7)	85.2 (54.1-127.3)	5.0 (2.2-8.2)
30 to 34	696.8 (550.2-862.4)	691.7 (545.8-857.4)	589.7 (465.2-732.1)	102.0 (64.8-153.4)	7.5 (3.4-12.0)
35 to 39	1,014.1 (811.0-1,243.4)	1,007.0 (805.1-1,237.8)	865.6 (691.1-1,075.0)	141.3 (91.9-208.5)	10.5 (4.8-16.6)
40 to 44	1,623.9 (1,296.0-2,006.5)	1,612.6 (1,285.3-1,993.9)	1,378.0 (1,100.5-1,722.3)	234.6 (152.8-338.7)	16.5 (7.5-26.7)
45 to 49	2,823.8 (2,269.7-3,439.6)	2,804.1 (2,257.5-3,419.6)	2,342.2 (1,885.4-2,872.8)	462.0 (302.3-670.1)	29.1 (12.8-46.7)
50 to 54	4,249.5 (3,407.0-5,168.1)	4,214.6 (3,386.4-5,131.1)	3,635.9 (2,869.9-4,469.7)	578.7 (381.2-829.2)	51.1 (22.9-82.4)
55 to 59	5,818.7 (4,697.5-7,043.6)	5,759.4 (4,645.9-6,967.1)	5,189.3 (4,151.7-6,305.7)	570.1 (377.3-822.8)	84.4 (38.8-136.0)
60 to 64	7,912.6 (6,423.2-9,486.1)	7,807.8 (6,319.7-9,396.5)	7,159.8 (5,783.5-8,717.6)	648.0 (424.9-931.4)	146.7 (67.6-235.7)
65 to 69	9,939.4 (8,113.2-11,780.4)	9,781.6 (7,999.2-11,642.5)	8,889.8 (7,286.6-10,633.2)	891.8 (591.5-1,247.9)	219.9 (99.5-348.0)
70 to 74	11,825.5 (9,764.3-13,971.5)	11,593.5 (9,561.0-13,708.1)	10,405.2 (8,568.9-12,422.2)	1,188.4 (783.8-1,657.8)	319.0 (147.4-514.1)
75 to 79	13,937.9 (11,510.8-16,380.6)	13,617.9 (11,212.3-16,009.5)	12,218.6 (10,026.4-14,554.2)	1,399.2 (929.1-1,964.4)	437.5 (205.1-698.7)
80 to 84	14,598.8 (12,156.7-17,155.8)	14,192.8 (11,886.8-16,652.1)	12,847.9 (10,669.2-15,330.0)	1,344.9 (900.6-1,911.6)	545.1 (252.4-875.9)
85 to 89	15,472.2 (12,591.5-18,400.9)	14,943.5 (12,188.2-17,846.4)	13,768.0 (11,103.4-16,469.4)	1,175.5 (768.1-1,714.0)	699.4 (327.4-1,116.3)
90 to 94	14,283.0 (11,437.9-17,333.0)	13,715.4 (10,979.0-16,649.0)	12,645.3 (10,046.5-15,366.0)	1,070.1 (682.3-1,546.5)	747.7 (341.8-1,216.4)
95 plus	12,738.3 (9,596.9-15,379.4)	12,179.5 (9,161.2-14,763.8)	11,141.0 (8,352.7-13,570.6)	1,038.5 (642.8-1,505.5)	735.7 (327.4-1,195.2)
All Ages	2,145.0 (1,831.0-2,485.2)	2,121.5 (1,813.9-2,461.1)	1,736.4 (1,456.5-2,039.7)	385.1 (265.7-513.5)	32.8 (15.0-51.9)

Supplementary Table 13. The number of DALYs attributable to air pollution and its sub-types by age-groups among countries in North Africa and Middle East in 2019

	Air pollution	Particulate matter pollution	Ambient particulate matter pollution	Household air pollution from solid fuels	Ambient ozone pollution
Under 5	2,504,541 (2,030,057-3,068,725)	2,504,541 (2,030,057-3,068,725)	1,382,654 (1,027,958-1,798,598)	1,121,887 (743,103-1,536,522)	
5 to 9	55,286 (36,809-77,973)	55,286 (36,809-77,973)	33,519 (21,663-49,887)	21,767 (11,445-35,119)	
10 to 14	32,517 (22,230-45,624)	32,517 (22,230-45,624)	22,160 (14,717-32,354)	10,357 (5,663-16,499)	
15 to 19	21,856 (14,941-32,040)	21,856 (14,941-32,040)	15,966 (10,287-23,696)	5,890 (3,144-9,623)	
20 to 24	21,583 (14,466-31,217)	21,583 (14,466-31,217)	15,903 (10,328-23,007)	5,681 (2,946-9,326)	
25 to 29	263,868 (211,408-328,875)	262,163 (210,043-327,160)	217,788 (173,364-271,285)	44,375 (28,165-66,300)	2,629 (1,171-4,253)
30 to 34	368,361 (290,893-455,945)	365,669 (288,541-453,270)	311,770 (245,912-387,063)	53,899 (34,247-81,073)	3,980 (1,796-6,356)
35 to 39	496,684 (397,215-609,003)	493,186 (394,317-606,236)	423,968 (338,484-526,524)	69,218 (45,025-102,129)	5,127 (2,334-8,132)
40 to 44	667,581 (532,784-824,872)	662,930 (528,362-819,658)	566,477 (452,420-708,014)	96,454 (62,796-139,221)	6,772 (3,074-10,966)
45 to 49	958,126 (770,119-1,167,056)	951,459 (765,967-1,160,267)	794,701 (639,712-974,749)	156,758 (102,562-227,353)	9,885 (4,346-15,842)
50 to 54	1,183,864 (949,147-1,439,756)	1,174,123 (943,400-1,429,456)	1,012,915 (799,518-1,245,196)	161,208 (106,191-231,016)	14,231 (6,366-22,966)
55 to 59	1,287,837 (1,039,689-1,558,935)	1,274,706 (1,028,274-1,542,011)	1,148,536 (918,874-1,395,632)	126,169 (83,513-182,110)	18,673 (8,579-30,107)
60 to 64	1,361,821 (1,105,488-1,632,632)	1,343,788 (1,087,679-1,617,219)	1,232,261 (995,389-1,500,366)	111,527 (73,121-160,296)	25,247 (11,629-40,566)
65 to 69	1,241,469 (1,013,376-1,471,417)	1,221,761 (999,132-1,454,197)	1,110,375 (910,125-1,328,128)	111,386 (73,876-155,873)	27,470 (12,427-43,468)
70 to 74	993,395 (820,248-1,173,668)	973,911 (803,165-1,151,541)	874,084 (719,823-1,043,517)	99,827 (65,846-139,259)	26,797 (12,386-43,185)
75 to 79	745,938 (616,042-876,671)	728,814 (600,071-856,811)	653,928 (536,603-778,922)	74,886 (49,722-105,134)	23,415 (10,975-37,391)
80 to 84	492,828 (410,389-579,150)	479,123 (401,276-562,146)	433,720 (360,174-517,513)	45,402 (30,403-64,534)	18,403 (8,521-29,569)
85 to 89	261,827 (213,078-311,387)	252,880 (206,253-302,005)	232,987 (187,897-278,702)	19,893 (12,999-29,004)	11,835 (5,540-18,891)
90 to 94	79,829 (63,927-96,876)	76,657 (61,362-93,053)	70,676 (56,151-85,882)	5,981 (3,813-8,644)	4,179 (1,910-6,798)
95 plus	17,651 (13,298-21,311)	16,877 (12,695-20,458)	15,438 (11,574-18,805)	1,439 (891-2,086)	1,019 (454-1,656)
All Ages	13,056,864 (11,145,813-15,128,048)	12,913,831 (11,041,330-14,981,099)	10,569,826 (8,866,135-12,416,213)	2,344,005 (1,617,635-3,125,700)	199,665 (91,412-316,069)

Supplementary Figure 1.a. Age-standardised death and DALYs rates per 100 000 attributable to air pollution among women in countries of North Africa and Middle East in 1990 and 2019

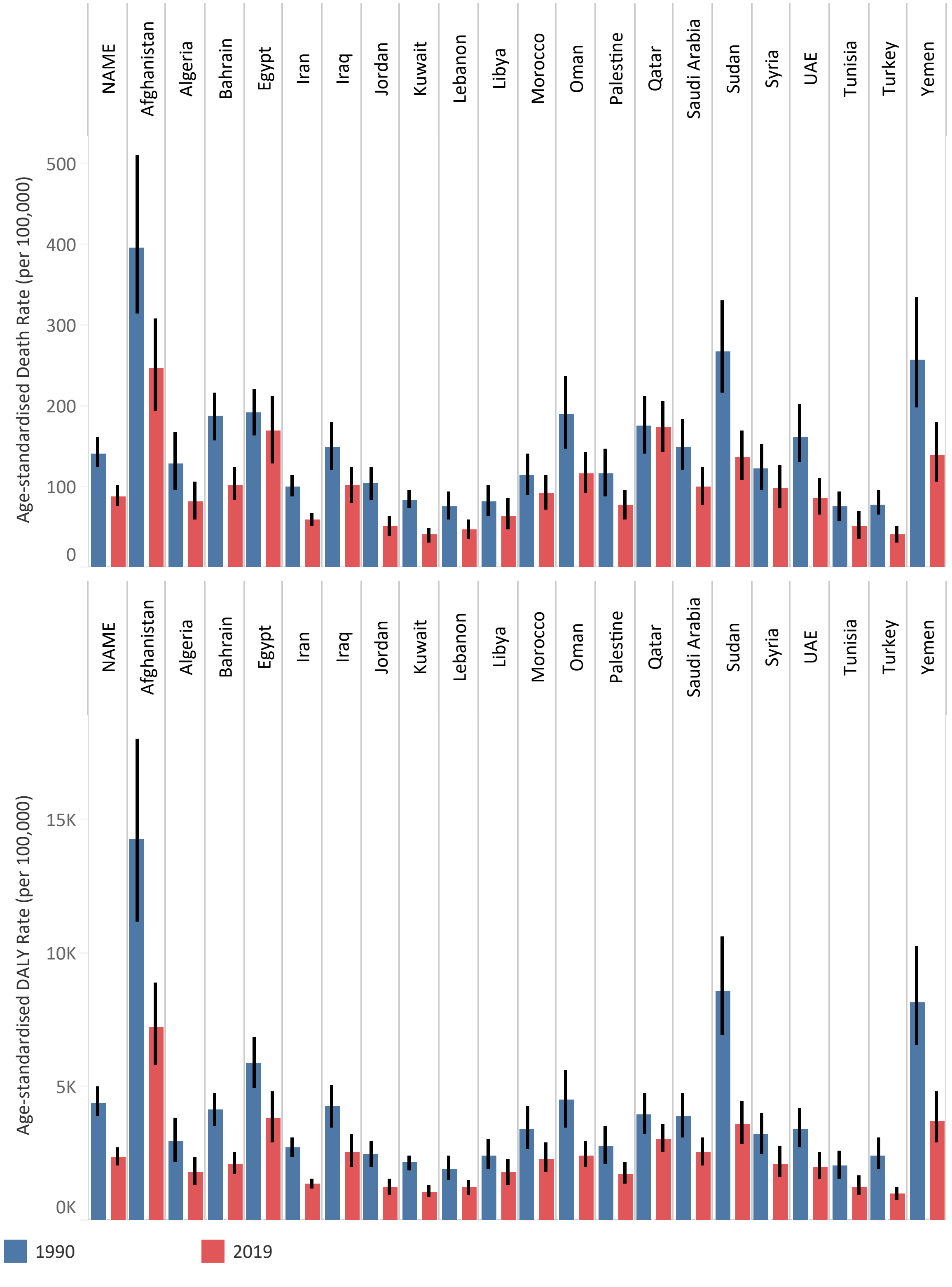

Supplementary Figure 1.b. Age-standardised death and DALYs rates per 100 000 attributable to air pollution among men in countries of North Africa and Middle East in 1990 and 2019

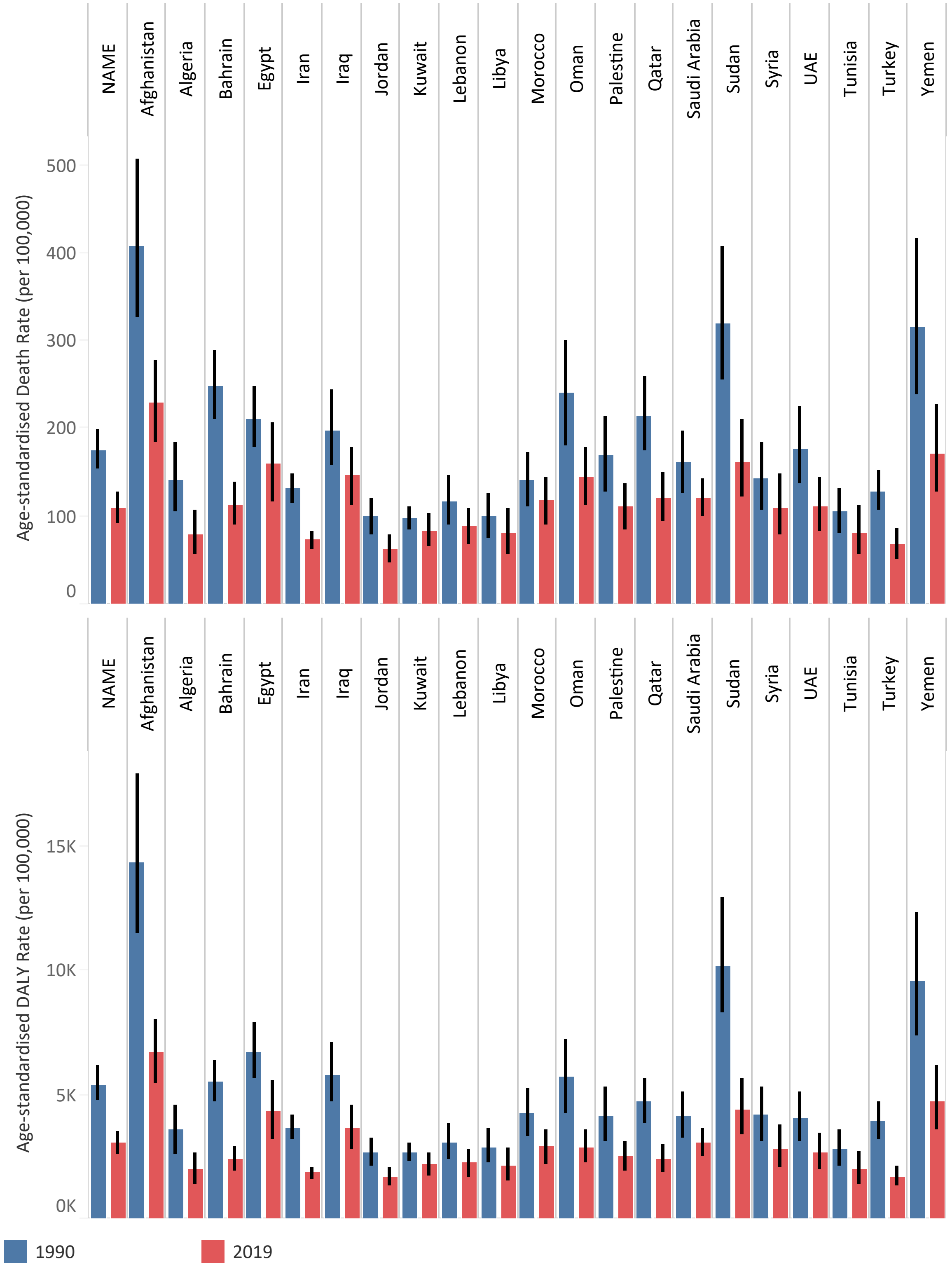

Supplementary Figure 1.c. Age-standardised death and DALYs rates per 100 000 attributable to air pollution among both sexes in countries of North Africa and Middle East in 1990 and 2019

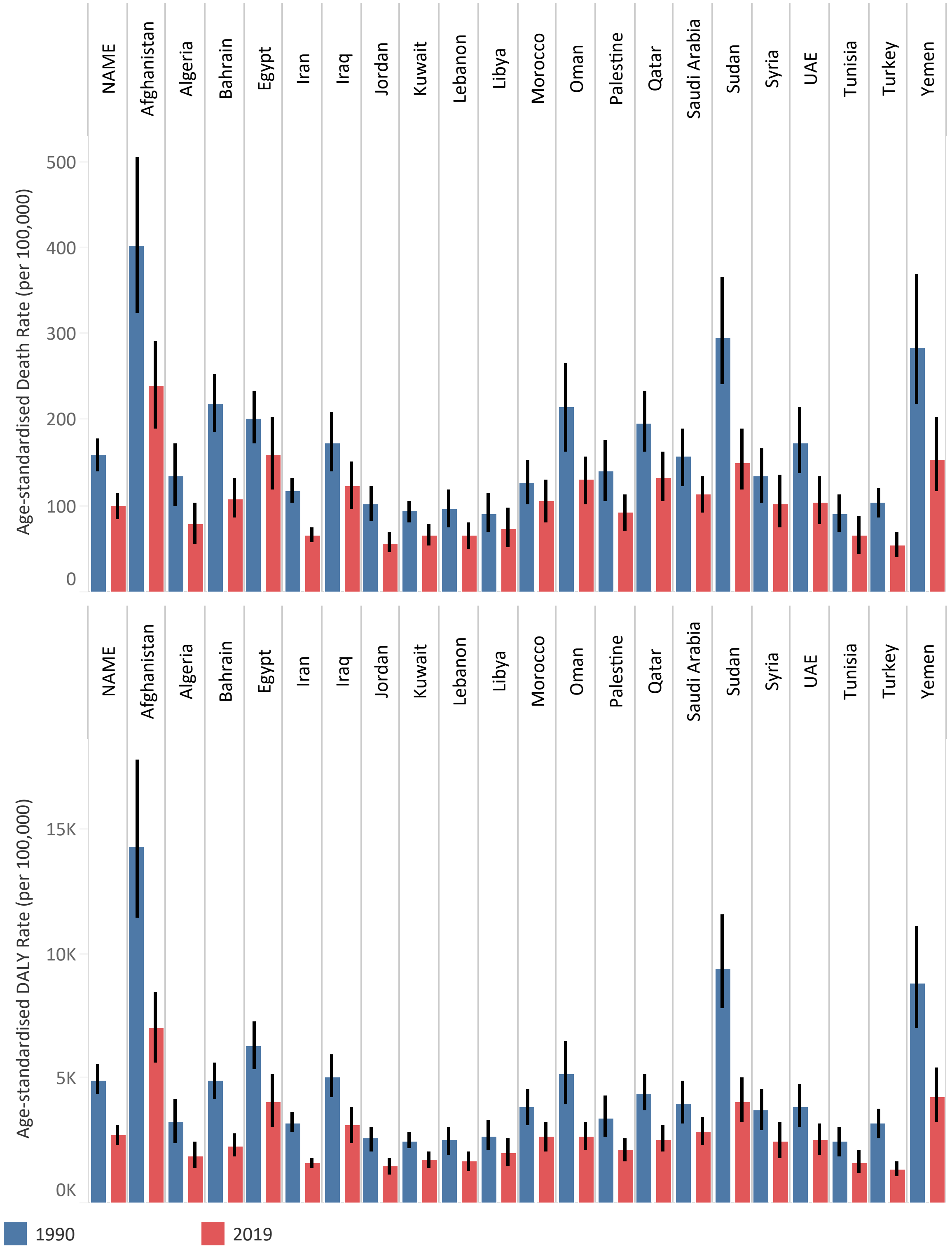

Supplementary Figure 2.a. Changes in life expectancy by age in North Africa and Middle East in 2019 if air pollution had been lowered to the theoretical minimum risk exposure levels among women

	All Ages	Under 1	1 to 4	5 to 9	10 to 14	15 to 19	20 to 24	25 to 29	30 to 34	35 to 39	40 to 44	45 to 49	50 to 54	55 to 59	60 to 64	65 to 69	70 to 74	75 to 79	80 to 84	85 plus
NAME	1.5 (3.0%)	1.6 (2.2%)	1.6 (2.2%)	1.6 (2.2%)	1.6 (2.3%)	1.6 (2.5%)	1.6 (2.7%)	1.6 (3.0%)	1.6 (3.2%)	1.5 (3.6%)	1.5 (4.0%)	1.5 (4.4%)	1.4 (4.9%)	1.4 (5.5%)	1.3 (6.1%)	1.2 (6.8%)	1.0 (7.5%)	0.8 (8.2%)	0.7 (8.8%)	0.5 (9.5%)
Afghanistan	2.3 (4.8%)	2.4 (3.8%)	2.4 (3.8%)	2.4 (3.9%)	2.4 (4.1%)	2.4 (4.5%)	2.4 (5.0%)	2.4 (5.6%)	2.4 (6.2%)	2.3 (6.9%)	2.3 (7.8%)	2.2 (8.7%)	2.1 (9.6%)	1.9 (10.5%)	1.7 (11.4%)	1.5 (12.2%)	1.3 (13.3%)	1.0 (14.1%)	0.8 (14.7%)	0.6 (15.3%)
Algeria	1.0 (2.0%)	1.0 (1.4%)	1.0 (1.4%)	1.0 (1.4%)	1.0 (1.5%)	1.0 (1.6%)	1.0 (1.8%)	1.0 (1.9%)	1.0 (2.1%)	1.0 (2.3%)	1.0 (2.6%)	1.0 (2.8%)	0.9 (3.1%)	0.9 (3.5%)	0.8 (3.9%)	0.7 (4.3%)	0.6 (4.8%)	0.5 (5.5%)	0.4 (6.2%)	0.3 (8.5%)
Bahrain	1.4 (2.9%)	1.4 (2.0%)	1.4 (2.0%)	1.4 (2.0%)	1.4 (2.1%)	1.4 (2.3%)	1.4 (2.5%)	1.4 (2.7%)	1.4 (3.0%)	1.4 (3.3%)	1.4 (3.7%)	1.4 (4.2%)	1.4 (4.8%)	1.4 (5.6%)	1.3 (6.5%)	1.2 (7.6%)	1.1 (8.8%)	0.9 (9.9%)	0.7 (10.9%)	0.5 (12.5%)
Egypt	1.7 (3.6%)	1.8 (2.7%)	1.8 (2.7%)	1.8 (2.7%)	1.8 (2.9%)	1.8 (3.1%)	1.8 (3.4%)	1.8 (3.8%)	1.8 (4.2%)	1.8 (4.6%)	1.8 (5.2%)	1.7 (5.9%)	1.7 (6.7%)	1.6 (7.6%)	1.4 (8.7%)	1.2 (9.5%)	1.0 (10.1%)	0.8 (10.5%)	0.6 (10.8%)	0.4 (11.2%)
Iran	1.2 (2.3%)	1.2 (1.6%)	1.2 (1.6%)	1.2 (1.6%)	1.2 (1.7%)	1.2 (1.9%)	1.2 (2.0%)	1.2 (2.2%)	1.2 (2.4%)	1.2 (2.6%)	1.2 (2.9%)	1.2 (3.3%)	1.2 (3.7%)	1.1 (4.2%)	1.1 (4.8%)	1.0 (5.5%)	0.9 (6.3%)	0.8 (7.3%)	0.7 (8.1%)	0.5 (9.1%)
Iraq	1.9 (3.5%)	1.9 (2.7%)	1.9 (2.7%)	1.9 (2.7%)	1.9 (2.9%)	1.9 (3.1%)	1.9 (3.4%)	1.9 (3.7%)	1.9 (4.0%)	1.9 (4.5%)	1.9 (5.0%)	1.9 (5.6%)	1.8 (6.2%)	1.7 (7.0%)	1.6 (7.8%)	1.5 (8.6%)	1.3 (9.4%)	1.1 (10.2%)	0.9 (10.7%)	0.6 (11.5%)
Jordan	1.0 (1.8%)	1.0 (1.3%)	1.0 (1.3%)	1.0 (1.3%)	1.0 (1.4%)	1.0 (1.5%)	1.0 (1.7%)	1.0 (1.8%)	1.0 (2.0%)	1.0 (2.2%)	1.0 (2.4%)	1.0 (2.7%)	1.0 (3.1%)	0.9 (3.5%)	0.9 (4.1%)	0.9 (4.7%)	0.8 (5.2%)	0.7 (5.8%)	0.5 (6.5%)	0.4 (7.4%)
Kuwait	1.6 (2.8%)	1.6 (2.0%)	1.6 (2.0%)	1.6 (2.0%)	1.6 (2.1%)	1.6 (2.2%)	1.6 (2.4%)	1.6 (2.6%)	1.6 (2.8%)	1.6 (3.1%)	1.6 (3.4%)	1.6 (3.8%)	1.6 (4.2%)	1.5 (4.8%)	1.5 (5.5%)	1.5 (6.3%)	1.4 (7.2%)	1.3 (8.3%)	1.2 (9.6%)	0.9 (11.0%)
Lebanon	1.0 (2.0%)	1.0 (1.4%)	1.0 (1.4%)	1.0 (1.4%)	1.0 (1.5%)	1.0 (1.6%)	1.0 (1.7%)	1.0 (1.9%)	1.0 (2.0%)	1.0 (2.2%)	1.0 (2.5%)	1.0 (2.8%)	1.0 (3.1%)	0.9 (3.5%)	0.9 (3.9%)	0.8 (4.4%)	0.7 (4.9%)	0.6 (5.4%)	0.5 (6.0%)	0.4 (6.7%)
Libya	1.5 (2.9%)	1.5 (2.0%)	1.5 (2.0%)	1.5 (2.1%)	1.5 (2.2%)	1.5 (2.4%)	1.5 (2.6%)	1.5 (2.8%)	1.5 (3.0%)	1.5 (3.3%)	1.5 (3.7%)	1.5 (4.1%)	1.4 (4.5%)	1.4 (5.1%)	1.3 (5.7%)	1.2 (6.2%)	1.0 (6.5%)	0.9 (7.1%)	0.7 (7.2%)	0.5 (8.1%)
Morocco	1.4 (3.0%)	1.5 (2.1%)	1.5 (2.1%)	1.5 (2.1%)	1.5 (2.2%)	1.5 (2.4%)	1.5 (2.6%)	1.5 (2.9%)	1.5 (3.2%)	1.5 (3.5%)	1.5 (3.9%)	1.4 (4.4%)	1.4 (4.9%)	1.3 (5.5%)	1.2 (6.2%)	1.1 (6.8%)	0.9 (7.4%)	0.8 (8.0%)	0.6 (8.4%)	0.4 (8.9%)
Oman	1.6 (3.0%)	1.6 (2.2%)	1.6 (2.2%)	1.6 (2.3%)	1.6 (2.4%)	1.6 (2.6%)	1.6 (2.8%)	1.6 (3.1%)	1.6 (3.4%)	1.6 (3.8%)	1.6 (4.3%)	1.6 (4.9%)	1.5 (5.6%)	1.5 (6.6%)	1.4 (7.6%)	1.3 (8.8%)	1.1 (10.0%)	0.9 (10.8%)	0.7 (11.1%)	0.5 (11.1%)
Palestine	1.2 (2.3%)	1.3 (1.7%)	1.3 (1.7%)	1.3 (1.8%)	1.3 (1.9%)	1.3 (2.0%)	1.3 (2.2%)	1.3 (2.4%)	1.3 (2.6%)	1.2 (2.9%)	1.2 (3.3%)	1.2 (3.7%)	1.2 (4.3%)	1.2 (4.9%)	1.1 (5.7%)	1.0 (6.6%)	0.9 (7.5%)	0.8 (8.2%)	0.6 (8.5%)	0.5 (8.9%)
Qatar	1.2 (2.4%)	1.2 (1.7%)	1.2 (1.7%)	1.2 (1.7%)	1.2 (1.8%)	1.2 (2.0%)	1.2 (2.1%)	1.2 (2.4%)	1.2 (2.6%)	1.2 (2.9%)	1.2 (3.3%)	1.2 (3.7%)	1.2 (4.3%)	1.1 (5.1%)	1.1 (6.2%)	1.0 (7.5%)	0.9 (9.1%)	0.7 (11.3%)	0.6 (15.4%)	0.4 (17.6%)
Saudi Arabia	1.9 (3.7%)	1.9 (2.7%)	1.9 (2.7%)	1.9 (2.7%)	1.9 (2.9%)	1.9 (3.1%)	1.9 (3.4%)	1.9 (3.7%)	1.9 (4.0%)	1.9 (4.5%)	1.9 (4.9%)	1.8 (5.5%)	1.8 (6.1%)	1.7 (6.7%)	1.5 (7.3%)	1.4 (7.9%)	1.2 (8.7%)	1.0 (9.6%)	0.9 (10.5%)	0.6 (11.8%)
Sudan	2.2 (4.1%)	2.2 (3.2%)	2.2 (3.2%)	2.2 (3.2%)	2.2 (3.4%)	2.2 (3.7%)	2.2 (4.0%)	2.2 (4.4%)	2.2 (4.8%)	2.2 (5.4%)	2.2 (6.0%)	2.2 (6.7%)	2.1 (7.5%)	2.0 (8.4%)	1.8 (9.4%)	1.7 (10.5%)	1.5 (11.7%)	1.2 (12.8%)	1.0 (13.7%)	0.7 (14.2%)
Syria	1.1 (2.3%)	1.2 (1.7%)	1.2 (1.7%)	1.2 (1.7%)	1.2 (1.8%)	1.2 (2.0%)	1.2 (2.1%)	1.2 (2.3%)	1.2 (2.5%)	1.2 (2.8%)	1.1 (3.1%)	1.1 (3.4%)	1.1 (3.8%)	1.0 (4.2%)	0.9 (4.8%)	0.8 (5.3%)	0.7 (5.7%)	0.5 (6.4%)	0.4 (6.9%)	0.2 (7.9%)
Tunisia	1.1 (2.3%)	1.2 (1.5%)	1.2 (1.5%)	1.2 (1.6%)	1.2 (1.6%)	1.2 (1.8%)	1.2 (1.9%)	1.2 (2.1%)	1.2 (2.3%)	1.2 (2.5%)	1.1 (2.7%)	1.1 (3.1%)	1.1 (3.4%)	1.1 (3.9%)	1.0 (4.4%)	1.0 (5.0%)	0.9 (5.6%)	0.8 (6.3%)	0.6 (7.0%)	0.5 (7.6%)
Turkey	0.9 (1.9%)	1.0 (1.3%)	1.0 (1.3%)	1.0 (1.3%)	1.0 (1.3%)	1.0 (1.4%)	1.0 (1.6%)	1.0 (1.7%)	1.0 (1.9%)	1.0 (2.0%)	1.0 (2.3%)	1.0 (2.5%)	0.9 (2.9%)	0.9 (3.3%)	0.9 (3.7%)	0.8 (4.3%)	0.8 (5.0%)	0.7 (5.8%)	0.6 (6.5%)	0.5 (7.2%)
UAE	1.4 (2.8%)	1.4 (1.9%)	1.4 (1.9%)	1.4 (1.9%)	1.4 (2.1%)	1.4 (2.2%)	1.4 (2.4%)	1.4 (2.6%)	1.4 (2.9%)	1.4 (3.2%)	1.4 (3.6%)	1.3 (4.0%)	1.3 (4.6%)	1.3 (5.3%)	1.2 (6.0%)	1.1 (6.9%)	1.0 (7.7%)	0.9 (8.8%)	0.8 (10.5%)	0.6 (11.4%)
Yemen	2.0 (3.8%)	2.0 (3.0%)	2.0 (3.0%)	2.0 (3.0%)	2.0 (3.2%)	2.0 (3.5%)	2.0 (3.8%)	2.1 (4.2%)	2.1 (4.6%)	2.1 (5.1%)	2.0 (5.7%)	2.0 (6.4%)	1.9 (7.1%)	1.8 (7.9%)	1.7 (8.8%)	1.5 (9.8%)	1.3 (10.7%)	1.1 (11.6%)	0.9 (12.5%)	0.6 (12.9%)

Supplementary Figure 2.b. Changes in life expectancy by age in North Africa and Middle East in 2019 if air pollution had been lowered to the theoretical minimum risk exposure levels among men

	All Ages	Under 1	1 to 4	5 to 9	10 to 14	15 to 19	20 to 24	25 to 29	30 to 34	35 to 39	40 to 44	45 to 49	50 to 54	55 to 59	60 to 64	65 to 69	70 to 74	75 to 79	80 to 84	85 plus
NAME	1.7 (3.6%)	1.8 (2.6%)	1.8 (2.6%)	1.8 (2.6%)	1.8 (2.8%)	1.8 (3.0%)	1.8 (3.3%)	1.8 (3.6%)	1.8 (3.9%)	1.8 (4.4%)	1.8 (4.9%)	1.7 (5.4%)	1.6 (6.1%)	1.5 (6.7%)	1.4 (7.3%)	1.2 (7.8%)	1.1 (8.3%)	0.9 (8.7%)	0.7 (8.9%)	0.5 (9.5%)
Afghanistan	2.3 (4.6%)	2.3 (3.7%)	2.3 (3.7%)	2.3 (3.8%)	2.3 (4.0%)	2.3 (4.4%)	2.3 (4.8%)	2.3 (5.3%)	2.3 (5.9%)	2.3 (6.7%)	2.3 (7.5%)	2.2 (8.4%)	2.1 (9.3%)	1.9 (10.1%)	1.7 (11.0%)	1.5 (11.7%)	1.2 (12.4%)	1.0 (12.9%)	0.8 (13.6%)	0.6 (14.7%)
Algeria	1.2 (2.4%)	1.3 (1.8%)	1.3 (1.8%)	1.3 (1.8%)	1.3 (1.9%)	1.3 (2.0%)	1.3 (2.2%)	1.3 (2.4%)	1.3 (2.6%)	1.3 (2.9%)	1.2 (3.2%)	1.2 (3.6%)	1.2 (3.9%)	1.1 (4.3%)	1.0 (4.8%)	0.9 (5.2%)	0.8 (5.7%)	0.6 (6.3%)	0.5 (7.0%)	0.4 (8.8%)
Bahrain	1.5 (3.5%)	1.5 (2.1%)	1.5 (2.1%)	1.5 (2.2%)	1.5 (2.3%)	1.5 (2.5%)	1.6 (2.7%)	1.6 (3.0%)	1.6 (3.2%)	1.5 (3.6%)	1.5 (4.0%)	1.5 (4.5%)	1.5 (5.1%)	1.4 (5.9%)	1.3 (6.8%)	1.2 (8.0%)	1.1 (9.5%)	0.9 (10.8%)	0.7 (11.6%)	0.6 (12.7%)
Egypt	2.2 (4.7%)	2.3 (3.5%)	2.3 (3.5%)	2.3 (3.5%)	2.3 (3.7%)	2.3 (4.1%)	2.3 (4.4%)	2.3 (4.9%)	2.3 (5.4%)	2.2 (6.0%)	2.2 (6.7%)	2.1 (7.6%)	2.0 (8.5%)	1.9 (9.3%)	1.7 (10.0%)	1.5 (10.4%)	1.2 (10.5%)	1.0 (10.8%)	0.8 (10.6%)	0.6 (10.8%)
Iran	1.4 (2.9%)	1.4 (2.0%)	1.4 (2.0%)	1.4 (2.0%)	1.4 (2.2%)	1.5 (2.3%)	1.5 (2.5%)	1.5 (2.8%)	1.5 (3.0%)	1.5 (3.3%)	1.4 (3.7%)	1.4 (4.1%)	1.4 (4.6%)	1.3 (5.1%)	1.2 (5.6%)	1.1 (6.1%)	1.0 (6.8%)	0.8 (7.5%)	0.7 (8.1%)	0.5 (9.1%)
Iraq	2.0 (4.1%)	2.1 (3.1%)	2.1 (3.1%)	2.1 (3.1%)	2.1 (3.3%)	2.1 (3.6%)	2.1 (3.9%)	2.1 (4.4%)	2.1 (4.8%)	2.1 (5.4%)	2.1 (6.1%)	2.0 (6.9%)	2.0 (7.7%)	1.8 (8.6%)	1.7 (9.4%)	1.5 (10.2%)	1.2 (10.7%)	1.0 (11.0%)	0.8 (11.0%)	0.5 (11.2%)
Jordan	1.3 (2.4%)	1.3 (1.8%)	1.3 (1.8%)	1.3 (1.8%)	1.3 (1.9%)	1.3 (2.1%)	1.3 (2.3%)	1.3 (2.5%)	1.3 (2.7%)	1.3 (3.0%)	1.3 (3.3%)	1.3 (3.7%)	1.3 (4.1%)	1.2 (4.6%)	1.1 (5.1%)	1.0 (5.6%)	0.9 (6.1%)	0.8 (6.6%)	0.6 (7.0%)	0.5 (7.3%)
Kuwait	2.1 (4.1%)	2.1 (2.8%)	2.1 (2.8%)	2.1 (2.9%)	2.1 (3.0%)	2.1 (3.3%)	2.1 (3.5%)	2.1 (3.8%)	2.1 (4.2%)	2.1 (4.6%)	2.1 (5.0%)	2.0 (5.5%)	2.0 (6.1%)	1.9 (6.7%)	1.7 (7.4%)	1.6 (8.2%)	1.4 (9.2%)	1.3 (10.2%)	1.1 (11.3%)	0.9 (13.0%)
Lebanon	1.3 (2.8%)	1.4 (2.0%)	1.4 (2.0%)	1.4 (2.0%)	1.4 (2.1%)	1.4 (2.3%)	1.4 (2.5%)	1.4 (2.8%)	1.4 (3.1%)	1.4 (3.4%)	1.4 (3.8%)	1.3 (4.2%)	1.3 (4.6%)	1.2 (5.1%)	1.1 (5.6%)	0.9 (6.0%)	0.8 (6.5%)	0.6 (6.9%)	0.5 (7.1%)	0.4 (7.5%)
Libya	1.5 (3.2%)	1.6 (2.3%)	1.6 (2.3%)	1.6 (2.3%)	1.6 (2.4%)	1.6 (2.6%)	1.6 (2.8%)	1.6 (3.1%)	1.6 (3.4%)	1.6 (3.7%)	1.6 (4.1%)	1.5 (4.6%)	1.5 (5.1%)	1.4 (5.7%)	1.3 (6.3%)	1.2 (6.8%)	1.0 (7.4%)	0.9 (7.8%)	0.7 (8.0%)	0.5 (8.7%)
Morocco	1.6 (3.4%)	1.6 (2.4%)	1.6 (2.4%)	1.6 (2.4%)	1.6 (2.6%)	1.6 (2.8%)	1.6 (3.0%)	1.6 (3.3%)	1.6 (3.7%)	1.6 (4.1%)	1.6 (4.6%)	1.6 (5.2%)	1.5 (5.9%)	1.4 (6.6%)	1.3 (7.3%)	1.1 (8.0%)	1.0 (8.5%)	0.8 (9.0%)	0.6 (9.2%)	0.4 (9.6%)
Oman	1.5 (3.1%)	1.5 (2.2%)	1.5 (2.2%)	1.5 (2.2%)	1.5 (2.3%)	1.5 (2.5%)	1.5 (2.7%)	1.5 (3.0%)	1.5 (3.3%)	1.5 (3.7%)	1.5 (4.2%)	1.5 (4.8%)	1.4 (5.5%)	1.4 (6.4%)	1.3 (7.5%)	1.2 (8.8%)	1.0 (9.7%)	0.8 (11.0%)	0.6 (12.6%)	0.4 (11.3%)
Palestine	1.4 (2.7%)	1.4 (2.1%)	1.4 (2.1%)	1.4 (2.1%)	1.4 (2.2%)	1.4 (2.4%)	1.4 (2.6%)	1.4 (2.9%)	1.4 (3.2%)	1.4 (3.6%)	1.4 (4.0%)	1.4 (4.5%)	1.3 (5.1%)	1.3 (5.8%)	1.1 (6.4%)	1.0 (6.9%)	0.8 (7.5%)	0.7 (8.0%)	0.5 (8.0%)	0.4 (8.5%)
Qatar	1.3 (2.8%)	1.3 (1.8%)	1.3 (1.8%)	1.3 (1.9%)	1.3 (2.0%)	1.3 (2.1%)	1.3 (2.3%)	1.3 (2.5%)	1.3 (2.8%)	1.3 (3.1%)	1.3 (3.5%)	1.3 (3.9%)	1.3 (4.4%)	1.2 (5.1%)	1.2 (5.9%)	1.1 (7.0%)	1.0 (8.5%)	0.8 (10.1%)	0.7 (12.2%)	0.5 (13.6%)
Saudi Arabia	2.0 (4.4%)	2.0 (3.0%)	2.0 (3.0%)	2.0 (3.0%)	2.0 (3.2%)	2.0 (3.5%)	2.0 (3.8%)	2.1 (4.2%)	2.1 (4.6%)	2.1 (5.1%)	2.0 (5.7%)	2.0 (6.4%)	1.9 (7.0%)	1.8 (7.7%)	1.6 (8.4%)	1.4 (8.9%)	1.2 (9.4%)	1.0 (10.0%)	0.8 (10.5%)	0.6 (11.4%)
Sudan	2.2 (4.3%)	2.2 (3.3%)	2.2 (3.3%)	2.2 (3.4%)	2.2 (3.6%)	2.3 (3.9%)	2.3 (4.3%)	2.3 (4.7%)	2.3 (5.2%)	2.2 (5.7%)	2.2 (6.4%)	2.1 (7.1%)	2.0 (7.9%)	1.9 (8.8%)	1.7 (9.7%)	1.5 (10.6%)	1.3 (11.5%)	1.1 (12.2%)	0.9 (12.9%)	0.6 (13.6%)
Syria	1.5 (3.3%)	1.6 (2.4%)	1.6 (2.4%)	1.6 (2.4%)	1.6 (2.6%)	1.6 (2.8%)	1.6 (3.0%)	1.6 (3.3%)	1.6 (3.6%)	1.6 (4.0%)	1.6 (4.5%)	1.5 (5.0%)	1.4 (5.5%)	1.3 (6.0%)	1.2 (6.5%)	1.0 (6.7%)	0.8 (6.9%)	0.7 (7.3%)	0.5 (7.6%)	0.4 (8.1%)
Tunisia	1.3 (2.8%)	1.4 (1.9%)	1.4 (1.9%)	1.4 (2.0%)	1.4 (2.1%)	1.4 (2.3%)	1.4 (2.5%)	1.4 (2.7%)	1.4 (3.0%)	1.4 (3.3%)	1.4 (3.6%)	1.3 (4.0%)	1.3 (4.5%)	1.2 (5.0%)	1.1 (5.5%)	1.0 (6.1%)	0.9 (6.7%)	0.7 (7.3%)	0.6 (8.0%)	0.4 (8.4%)
Turkey	1.3 (2.7%)	1.3 (1.8%)	1.3 (1.8%)	1.3 (1.9%)	1.3 (2.0%)	1.3 (2.1%)	1.3 (2.3%)	1.3 (2.5%)	1.3 (2.8%)	1.3 (3.1%)	1.3 (3.4%)	1.3 (3.8%)	1.3 (4.3%)	1.2 (4.8%)	1.1 (5.3%)	1.0 (5.8%)	0.9 (6.4%)	0.8 (6.8%)	0.6 (7.0%)	0.5 (7.7%)
UAE	1.5 (3.8%)	1.6 (2.3%)	1.6 (2.3%)	1.6 (2.3%)	1.6 (2.5%)	1.6 (2.7%)	1.6 (2.9%)	1.6 (3.2%)	1.6 (3.6%)	1.6 (4.0%)	1.6 (4.5%)	1.5 (5.0%)	1.5 (5.6%)	1.4 (6.3%)	1.3 (7.0%)	1.1 (7.7%)	1.0 (8.4%)	0.8 (9.5%)	0.7 (10.0%)	0.5 (10.9%)
Yemen	2.0 (4.1%)	2.0 (3.2%)	2.0 (3.2%)	2.0 (3.2%)	2.0 (3.4%)	2.0 (3.7%)	2.1 (4.1%)	2.1 (4.6%)	2.1 (5.1%)	2.1 (5.6%)	2.1 (6.3%)	2.0 (7.1%)	1.9 (7.9%)	1.8 (8.7%)	1.6 (9.5%)	1.4 (10.2%)	1.2 (10.9%)	1.0 (11.4%)	0.8 (12.0%)	0.6 (12.5%)

Supplementary Figure 2.c. Changes in life expectancy by age in North Africa and Middle East in 2019 if air pollution had been lowered to the theoretical minimum risk exposure levels among both sexes

	All Ages	Under 1	1 to 4	5 to 9	10 to 14	15 to 19	20 to 24	25 to 29	30 to 34	35 to 39	40 to 44	45 to 49	50 to 54	55 to 59	60 to 64	65 to 69	70 to 74	75 to 79	80 to 84	85 plus
NAME	1.6 (3.3%)	1.7 (2.4%)	1.7 (2.4%)	1.7 (2.4%)	1.7 (2.6%)	1.7 (2.8%)	1.7 (3.0%)	1.7 (3.3%)	1.7 (3.6%)	1.7 (4.0%)	1.7 (4.5%)	1.6 (5.0%)	1.6 (5.5%)	1.5 (6.1%)	1.3 (6.7%)	1.2 (7.3%)	1.0 (7.9%)	0.9 (8.4%)	0.7 (8.8%)	0.5 (9.5%)
Afghanistan	2.3 (4.7%)	2.3 (3.8%)	2.3 (3.8%)	2.3 (3.8%)	2.3 (4.0%)	2.3 (4.4%)	2.3 (4.9%)	2.3 (5.4%)	2.3 (6.1%)	2.3 (6.8%)	2.3 (7.6%)	2.2 (8.6%)	2.1 (9.4%)	1.9 (10.3%)	1.7 (11.2%)	1.5 (12.0%)	1.3 (12.9%)	1.0 (13.7%)	0.8 (14.2%)	0.6 (15.0%)
Algeria	1.1 (2.2%)	1.2 (1.6%)	1.2 (1.6%)	1.2 (1.6%)	1.2 (1.7%)	1.2 (1.9%)	1.2 (2.0%)	1.2 (2.2%)	1.2 (2.4%)	1.2 (2.6%)	1.1 (2.9%)	1.1 (3.2%)	1.1 (3.6%)	1.0 (4.0%)	0.9 (4.4%)	0.8 (4.8%)	0.7 (5.3%)	0.6 (5.9%)	0.5 (6.7%)	0.3 (9.1%)
Bahrain	1.5 (3.2%)	1.5 (2.1%)	1.5 (2.1%)	1.5 (2.1%)	1.5 (2.2%)	1.5 (2.4%)	1.5 (2.6%)	1.5 (2.9%)	1.5 (3.1%)	1.5 (3.5%)	1.5 (3.9%)	1.5 (4.4%)	1.4 (5.0%)	1.4 (5.7%)	1.3 (6.7%)	1.2 (7.8%)	1.1 (9.1%)	0.9 (10.3%)	0.7 (11.2%)	0.5 (12.6%)
Egypt	2.0 (4.2%)	2.1 (3.1%)	2.1 (3.1%)	2.1 (3.2%)	2.1 (3.4%)	2.1 (3.6%)	2.1 (4.0%)	2.1 (4.4%)	2.1 (4.8%)	2.0 (5.4%)	2.0 (6.0%)	2.0 (6.8%)	1.9 (7.7%)	1.8 (8.6%)	1.6 (9.5%)	1.4 (10.1%)	1.1 (10.6%)	0.9 (11.0%)	0.7 (11.3%)	0.5 (11.2%)
Iran	1.3 (2.6%)	1.3 (1.8%)	1.3 (1.8%)	1.3 (1.8%)	1.3 (2.0%)	1.3 (2.1%)	1.3 (2.3%)	1.4 (2.5%)	1.3 (2.7%)	1.3 (3.0%)	1.3 (3.3%)	1.3 (3.7%)	1.3 (4.1%)	1.2 (4.6%)	1.1 (5.2%)	1.1 (5.8%)	0.9 (6.5%)	0.8 (7.4%)	0.7 (8.1%)	0.5 (9.1%)
Iraq	2.0 (3.9%)	2.0 (2.9%)	2.0 (2.9%)	2.0 (3.0%)	2.0 (3.1%)	2.0 (3.4%)	2.0 (3.7%)	2.1 (4.1%)	2.1 (4.5%)	2.0 (5.0%)	2.0 (5.6%)	2.0 (6.3%)	1.9 (7.1%)	1.8 (7.9%)	1.7 (8.7%)	1.5 (9.4%)	1.3 (10.1%)	1.0 (10.6%)	0.8 (10.9%)	0.6 (11.4%)
Jordan	1.2 (2.1%)	1.2 (1.6%)	1.2 (1.6%)	1.2 (1.6%)	1.2 (1.7%)	1.2 (1.8%)	1.2 (2.0%)	1.2 (2.2%)	1.2 (2.4%)	1.2 (2.6%)	1.2 (2.9%)	1.2 (3.3%)	1.1 (3.7%)	1.1 (4.1%)	1.0 (4.6%)	0.9 (5.2%)	0.8 (5.7%)	0.7 (6.3%)	0.6 (6.7%)	0.4 (7.4%)
Kuwait	1.9 (3.6%)	2.0 (2.5%)	2.0 (2.5%)	2.0 (2.6%)	2.0 (2.7%)	2.0 (2.9%)	2.0 (3.1%)	2.0 (3.4%)	2.0 (3.7%)	1.9 (4.0%)	1.9 (4.4%)	1.9 (4.9%)	1.8 (5.5%)	1.8 (6.1%)	1.7 (6.8%)	1.6 (7.6%)	1.4 (8.6%)	1.3 (9.7%)	1.1 (10.9%)	0.9 (12.4%)
Lebanon	1.2 (2.4%)	1.2 (1.7%)	1.2 (1.7%)	1.2 (1.7%)	1.2 (1.8%)	1.2 (2.0%)	1.2 (2.1%)	1.2 (2.3%)	1.2 (2.5%)	1.2 (2.8%)	1.2 (3.1%)	1.2 (3.4%)	1.1 (3.8%)	1.1 (4.3%)	1.0 (4.7%)	0.9 (5.2%)	0.8 (5.7%)	0.6 (6.1%)	0.5 (6.6%)	0.4 (7.1%)
Libya	1.5 (3.1%)	1.6 (2.2%)	1.6 (2.2%)	1.6 (2.2%)	1.6 (2.3%)	1.6 (2.5%)	1.6 (2.7%)	1.6 (3.0%)	1.6 (3.3%)	1.6 (3.6%)	1.5 (4.0%)	1.5 (4.4%)	1.5 (4.9%)	1.4 (5.4%)	1.3 (6.0%)	1.2 (6.5%)	1.0 (7.0%)	0.9 (7.5%)	0.7 (7.6%)	0.5 (8.5%)
Morocco	1.5 (3.2%)	1.6 (2.2%)	1.6 (2.2%)	1.6 (2.3%)	1.6 (2.4%)	1.6 (2.6%)	1.6 (2.9%)	1.6 (3.1%)	1.6 (3.5%)	1.6 (3.8%)	1.6 (4.3%)	1.5 (4.9%)	1.5 (5.5%)	1.4 (6.1%)	1.3 (6.8%)	1.1 (7.4%)	1.0 (8.0%)	0.8 (8.5%)	0.6 (8.7%)	0.4 (9.1%)
Oman	1.5 (3.1%)	1.5 (2.2%)	1.5 (2.2%)	1.5 (2.2%)	1.5 (2.4%)	1.5 (2.6%)	1.6 (2.8%)	1.6 (3.1%)	1.6 (3.4%)	1.6 (3.8%)	1.5 (4.3%)	1.5 (4.9%)	1.5 (5.6%)	1.4 (6.5%)	1.4 (7.6%)	1.2 (8.9%)	1.1 (10.0%)	0.9 (11.1%)	0.7 (12.0%)	0.4 (11.1%)
Palestine	1.3 (2.5%)	1.4 (1.9%)	1.4 (1.9%)	1.4 (2.0%)	1.4 (2.1%)	1.4 (2.2%)	1.4 (2.4%)	1.4 (2.7%)	1.4 (2.9%)	1.4 (3.3%)	1.3 (3.7%)	1.3 (4.2%)	1.3 (4.7%)	1.2 (5.4%)	1.1 (6.1%)	1.0 (6.8%)	0.9 (7.6%)	0.7 (8.2%)	0.6 (8.4%)	0.4 (8.9%)
Qatar	1.3 (2.7%)	1.3 (1.8%)	1.3 (1.8%)	1.3 (1.8%)	1.3 (1.9%)	1.3 (2.1%)	1.3 (2.3%)	1.3 (2.5%)	1.3 (2.8%)	1.3 (3.1%)	1.3 (3.4%)	1.3 (3.9%)	1.3 (4.5%)	1.2 (5.2%)	1.2 (6.1%)	1.1 (7.3%)	1.0 (8.9%)	0.8 (10.8%)	0.7 (13.3%)	0.5 (14.4%)
Saudi Arabia	2.0 (4.1%)	2.0 (2.9%)	2.0 (2.9%)	2.0 (2.9%)	2.0 (3.1%)	2.0 (3.3%)	2.0 (3.6%)	2.0 (4.0%)	2.0 (4.4%)	2.0 (4.9%)	2.0 (5.4%)	1.9 (6.0%)	1.9 (6.7%)	1.8 (7.3%)	1.6 (8.0%)	1.4 (8.6%)	1.2 (9.2%)	1.0 (9.9%)	0.8 (10.6%)	0.6 (11.7%)
Sudan	2.2 (4.2%)	2.2 (3.3%)	2.2 (3.3%)	2.2 (3.3%)	2.2 (3.5%)	2.2 (3.8%)	2.2 (4.1%)	2.3 (4.6%)	2.2 (5.0%)	2.2 (5.6%)	2.2 (6.2%)	2.1 (6.9%)	2.1 (7.8%)	1.9 (8.6%)	1.8 (9.6%)	1.6 (10.6%)	1.4 (11.6%)	1.2 (12.5%)	0.9 (13.2%)	0.7 (13.8%)
Syria	1.4 (2.9%)	1.5 (2.1%)	1.5 (2.1%)	1.5 (2.1%)	1.5 (2.2%)	1.5 (2.4%)	1.5 (2.6%)	1.5 (2.9%)	1.4 (3.2%)	1.4 (3.5%)	1.4 (3.8%)	1.4 (4.3%)	1.3 (4.8%)	1.2 (5.3%)	1.1 (5.7%)	0.9 (6.1%)	0.8 (6.5%)	0.6 (6.9%)	0.5 (7.5%)	0.3 (8.4%)
Tunisia	1.2 (2.6%)	1.3 (1.8%)	1.3 (1.8%)	1.3 (1.8%)	1.3 (1.9%)	1.3 (2.1%)	1.3 (2.2%)	1.3 (2.4%)	1.3 (2.7%)	1.3 (2.9%)	1.3 (3.2%)	1.3 (3.6%)	1.2 (4.0%)	1.2 (4.5%)	1.1 (5.1%)	1.0 (5.7%)	0.9 (6.3%)	0.8 (6.9%)	0.6 (7.5%)	0.4 (7.9%)
Turkey	1.1 (2.3%)	1.2 (1.6%)	1.2 (1.6%)	1.2 (1.6%)	1.2 (1.7%)	1.2 (1.8%)	1.2 (1.9%)	1.2 (2.1%)	1.2 (2.3%)	1.2 (2.6%)	1.2 (2.8%)	1.1 (3.2%)	1.1 (3.6%)	1.1 (4.0%)	1.0 (4.5%)	0.9 (5.0%)	0.8 (5.6%)	0.7 (6.2%)	0.6 (6.8%)	0.5 (7.3%)
UAE	1.5 (3.5%)	1.5 (2.2%)	1.5 (2.2%)	1.5 (2.2%)	1.5 (2.4%)	1.5 (2.6%)	1.5 (2.8%)	1.5 (3.1%)	1.5 (3.4%)	1.5 (3.8%)	1.5 (4.2%)	1.5 (4.8%)	1.4 (5.3%)	1.4 (6.0%)	1.2 (6.7%)	1.1 (7.4%)	1.0 (8.0%)	0.8 (9.1%)	0.7 (10.1%)	0.5 (11.0%)
Yemen	2.0 (3.9%)	2.0 (3.1%)	2.0 (3.1%)	2.0 (3.1%)	2.0 (3.3%)	2.0 (3.6%)	2.1 (4.0%)	2.1 (4.4%)	2.1 (4.8%)	2.1 (5.4%)	2.1 (6.0%)	2.0 (6.8%)	1.9 (7.5%)	1.8 (8.3%)	1.6 (9.2%)	1.4 (10.0%)	1.2 (10.8%)	1.0 (11.5%)	0.8 (12.2%)	0.6 (12.6%)

Supplementary Figure 3. Changes in life expectancy by age in North Africa and Middle East in 2019 if PM pollution had been lowered to the theoretical minimum risk exposure levels

	All Ages	Under 1	1 to 4	5 to 9	10 to 14	15 to 19	20 to 24	25 to 29	30 to 34	35 to 39	40 to 44	45 to 49	50 to 54	55 to 59	60 to 64	65 to 69	70 to 74	75 to 79	80 to 84	85 plus
NAME	1.6 (3.2%)	1.7 (2.3%)	1.7 (2.3%)	1.7 (2.4%)	1.7 (2.5%)	1.7 (2.7%)	1.7 (3.0%)	1.7 (3.2%)	1.7 (3.6%)	1.6 (3.9%)	1.6 (4.4%)	1.6 (4.9%)	1.5 (5.4%)	1.4 (6.0%)	1.3 (6.5%)	1.2 (7.1%)	1.0 (7.6%)	0.8 (8.2%)	0.7 (8.5%)	0.5 (9.1%)
Afghanistan	2.3 (4.7%)	2.3 (3.7%)	2.3 (3.7%)	2.3 (3.8%)	2.3 (4.0%)	2.3 (4.4%)	2.3 (4.8%)	2.3 (5.4%)	2.3 (6.0%)	2.3 (6.7%)	2.3 (7.6%)	2.2 (8.5%)	2.1 (9.4%)	1.9 (10.3%)	1.7 (11.1%)	1.5 (11.9%)	1.2 (12.7%)	1.0 (13.4%)	0.8 (14.0%)	0.6 (14.8%)
Algeria	1.1 (2.2%)	1.1 (1.6%)	1.1 (1.6%)	1.1 (1.6%)	1.1 (1.7%)	1.1 (1.8%)	1.1 (2.0%)	1.1 (2.1%)	1.1 (2.4%)	1.1 (2.6%)	1.1 (2.8%)	1.1 (3.2%)	1.0 (3.5%)	1.0 (3.9%)	0.9 (4.3%)	0.8 (4.7%)	0.7 (5.1%)	0.6 (5.7%)	0.4 (6.5%)	0.3 (8.7%)
Bahrain	1.4 (3.2%)	1.5 (2.0%)	1.5 (2.0%)	1.5 (2.0%)	1.5 (2.2%)	1.5 (2.3%)	1.5 (2.5%)	1.5 (2.8%)	1.5 (3.0%)	1.5 (3.4%)	1.5 (3.8%)	1.4 (4.3%)	1.4 (4.8%)	1.4 (5.6%)	1.3 (6.5%)	1.2 (7.5%)	1.1 (8.8%)	0.9 (9.9%)	0.7 (10.7%)	0.5 (12.1%)
Egypt	2.0 (4.2%)	2.1 (3.1%)	2.1 (3.1%)	2.1 (3.1%)	2.1 (3.3%)	2.1 (3.6%)	2.1 (3.9%)	2.1 (4.3%)	2.0 (4.8%)	2.0 (5.3%)	2.0 (5.9%)	1.9 (6.7%)	1.9 (7.6%)	1.7 (8.5%)	1.6 (9.4%)	1.3 (9.9%)	1.1 (10.5%)	0.9 (10.9%)	0.7 (11.1%)	0.5 (11.0%)
Iran	1.3 (2.5%)	1.3 (1.8%)	1.3 (1.8%)	1.3 (1.8%)	1.3 (1.9%)	1.3 (2.0%)	1.3 (2.2%)	1.3 (2.4%)	1.3 (2.6%)	1.3 (2.9%)	1.3 (3.2%)	1.3 (3.6%)	1.2 (4.0%)	1.2 (4.4%)	1.1 (5.0%)	1.0 (5.6%)	0.9 (6.2%)	0.8 (7.1%)	0.7 (7.7%)	0.5 (8.6%)
Iraq	2.0 (3.9%)	2.0 (2.9%)	2.0 (2.9%)	2.0 (2.9%)	2.0 (3.1%)	2.0 (3.4%)	2.0 (3.7%)	2.0 (4.0%)	2.0 (4.5%)	2.0 (5.0%)	2.0 (5.6%)	2.0 (6.3%)	1.9 (7.0%)	1.8 (7.8%)	1.7 (8.6%)	1.5 (9.4%)	1.3 (10.0%)	1.0 (10.5%)	0.8 (10.8%)	0.6 (11.2%)
Jordan	1.1 (2.1%)	1.2 (1.6%)	1.2 (1.6%)	1.2 (1.6%)	1.2 (1.7%)	1.2 (1.8%)	1.2 (2.0%)	1.2 (2.1%)	1.2 (2.3%)	1.2 (2.6%)	1.2 (2.9%)	1.1 (3.2%)	1.1 (3.6%)	1.1 (4.0%)	1.0 (4.5%)	0.9 (5.0%)	0.8 (5.5%)	0.7 (6.1%)	0.6 (6.5%)	0.4 (7.2%)
Kuwait	1.9 (3.5%)	1.9 (2.5%)	1.9 (2.5%)	1.9 (2.5%)	1.9 (2.6%)	1.9 (2.8%)	1.9 (3.1%)	1.9 (3.3%)	1.9 (3.6%)	1.9 (4.0%)	1.9 (4.4%)	1.9 (4.8%)	1.8 (5.3%)	1.7 (5.9%)	1.6 (6.6%)	1.5 (7.4%)	1.4 (8.3%)	1.3 (9.4%)	1.1 (10.6%)	0.9 (12.1%)
Lebanon	1.1 (2.3%)	1.2 (1.7%)	1.2 (1.7%)	1.2 (1.7%)	1.2 (1.8%)	1.2 (1.9%)	1.2 (2.1%)	1.2 (2.3%)	1.2 (2.5%)	1.2 (2.8%)	1.2 (3.0%)	1.1 (3.4%)	1.1 (3.8%)	1.0 (4.2%)	1.0 (4.6%)	0.9 (5.1%)	0.8 (5.5%)	0.6 (5.9%)	0.5 (6.3%)	0.4 (6.8%)
Libya	1.5 (3.0%)	1.5 (2.1%)	1.5 (2.1%)	1.5 (2.1%)	1.5 (2.3%)	1.5 (2.5%)	1.5 (2.7%)	1.5 (2.9%)	1.5 (3.2%)	1.5 (3.5%)	1.5 (3.9%)	1.5 (4.3%)	1.4 (4.7%)	1.3 (5.2%)	1.3 (5.8%)	1.1 (6.3%)	1.0 (6.7%)	0.9 (7.2%)	0.7 (7.3%)	0.5 (8.0%)
Morocco	1.5 (3.1%)	1.5 (2.2%)	1.5 (2.2%)	1.5 (2.2%)	1.5 (2.4%)	1.5 (2.6%)	1.5 (2.8%)	1.5 (3.1%)	1.5 (3.4%)	1.5 (3.8%)	1.5 (4.2%)	1.5 (4.8%)	1.4 (5.3%)	1.4 (6.0%)	1.2 (6.6%)	1.1 (7.2%)	0.9 (7.8%)	0.8 (8.2%)	0.6 (8.5%)	0.4 (8.8%)
Oman	1.5 (3.1%)	1.5 (2.2%)	1.5 (2.2%)	1.5 (2.2%)	1.5 (2.3%)	1.5 (2.5%)	1.5 (2.8%)	1.5 (3.0%)	1.5 (3.4%)	1.5 (3.7%)	1.5 (4.2%)	1.5 (4.8%)	1.5 (5.5%)	1.4 (6.4%)	1.3 (7.5%)	1.2 (8.7%)	1.0 (9.8%)	0.9 (10.9%)	0.7 (11.7%)	0.4 (10.8%)
Palestine	1.3 (2.5%)	1.3 (1.9%)	1.3 (1.9%)	1.3 (1.9%)	1.3 (2.0%)	1.3 (2.2%)	1.3 (2.4%)	1.3 (2.6%)	1.3 (2.9%)	1.3 (3.2%)	1.3 (3.6%)	1.3 (4.1%)	1.3 (4.7%)	1.2 (5.3%)	1.1 (6.0%)	1.0 (6.7%)	0.9 (7.4%)	0.7 (8.0%)	0.6 (8.1%)	0.4 (8.7%)
Qatar	1.3 (2.7%)	1.3 (1.8%)	1.3 (1.8%)	1.3 (1.8%)	1.3 (1.9%)	1.3 (2.1%)	1.3 (2.3%)	1.3 (2.5%)	1.3 (2.7%)	1.3 (3.0%)	1.3 (3.4%)	1.3 (3.9%)	1.2 (4.4%)	1.2 (5.1%)	1.1 (6.0%)	1.1 (7.2%)	1.0 (8.7%)	0.8 (10.6%)	0.6 (13.1%)	0.5 (14.0%)
Saudi Arabia	1.9 (4.1%)	2.0 (2.8%)	2.0 (2.8%)	2.0 (2.9%)	2.0 (3.0%)	2.0 (3.3%)	2.0 (3.6%)	2.0 (3.9%)	2.0 (4.3%)	2.0 (4.8%)	2.0 (5.3%)	1.9 (6.0%)	1.8 (6.6%)	1.7 (7.2%)	1.6 (7.9%)	1.4 (8.4%)	1.2 (9.0%)	1.0 (9.6%)	0.8 (10.3%)	0.6 (11.3%)
Sudan	2.2 (4.1%)	2.2 (3.2%)	2.2 (3.2%)	2.2 (3.3%)	2.2 (3.5%)	2.2 (3.8%)	2.2 (4.1%)	2.2 (4.5%)	2.2 (5.0%)	2.2 (5.5%)	2.2 (6.1%)	2.1 (6.9%)	2.0 (7.7%)	1.9 (8.6%)	1.8 (9.5%)	1.6 (10.5%)	1.4 (11.4%)	1.2 (12.4%)	0.9 (13.0%)	0.7 (13.7%)
Syria	1.3 (2.8%)	1.4 (2.0%)	1.4 (2.0%)	1.4 (2.1%)	1.4 (2.2%)	1.4 (2.4%)	1.4 (2.6%)	1.4 (2.8%)	1.4 (3.1%)	1.4 (3.4%)	1.4 (3.8%)	1.3 (4.2%)	1.3 (4.7%)	1.2 (5.1%)	1.0 (5.6%)	0.9 (6.0%)	0.7 (6.3%)	0.6 (6.8%)	0.5 (7.3%)	0.3 (8.1%)
Tunisia	1.2 (2.5%)	1.3 (1.7%)	1.3 (1.7%)	1.3 (1.7%)	1.3 (1.8%)	1.3 (2.0%)	1.3 (2.2%)	1.3 (2.4%)	1.3 (2.6%)	1.3 (2.8%)	1.3 (3.1%)	1.2 (3.5%)	1.2 (3.9%)	1.1 (4.4%)	1.1 (4.9%)	1.0 (5.5%)	0.9 (6.1%)	0.7 (6.6%)	0.6 (7.2%)	0.4 (7.7%)
Turkey	1.1 (2.2%)	1.1 (1.5%)	1.1 (1.5%)	1.1 (1.5%)	1.1 (1.6%)	1.1 (1.7%)	1.1 (1.9%)	1.1 (2.0%)	1.1 (2.2%)	1.1 (2.4%)	1.1 (2.7%)	1.1 (3.0%)	1.1 (3.4%)	1.0 (3.8%)	1.0 (4.2%)	0.9 (4.7%)	0.8 (5.3%)	0.7 (5.8%)	0.6 (6.3%)	0.4 (6.8%)
UAE	1.5 (3.4%)	1.5 (2.1%)	1.5 (2.1%)	1.5 (2.2%)	1.5 (2.3%)	1.5 (2.5%)	1.5 (2.7%)	1.5 (3.0%)	1.5 (3.3%)	1.5 (3.7%)	1.5 (4.1%)	1.4 (4.6%)	1.4 (5.2%)	1.3 (5.8%)	1.2 (6.5%)	1.1 (7.1%)	0.9 (7.7%)	0.8 (8.8%)	0.7 (9.7%)	0.5 (10.6%)
Yemen	2.0 (3.9%)	2.0 (3.0%)	2.0 (3.0%)	2.0 (3.1%)	2.0 (3.3%)	2.0 (3.6%)	2.0 (3.9%)	2.0 (4.3%)	2.0 (4.8%)	2.0 (5.3%)	2.0 (5.9%)	2.0 (6.6%)	1.9 (7.4%)	1.8 (8.1%)	1.6 (9.0%)	1.4 (9.8%)	1.2 (10.7%)	1.0 (11.3%)	0.8 (11.9%)	0.6 (12.3%)

Supplementary Figure 4. Changes in life expectancy by age in North Africa and Middle East in 2019 if household air pollution from solid fuels had been lowered to the theoretical minimum risk exposure levels

	All Ages	Under 1	1 to 4	5 to 9	10 to 14	15 to 19	20 to 24	25 to 29	30 to 34	35 to 39	40 to 44	45 to 49	50 to 54	55 to 59	60 to 64	65 to 69	70 to 74	75 to 79	80 to 84	85 plus
NAME	0.2 (0.3%)	0.2 (0.2%)	0.2 (0.2%)	0.2 (0.2%)	0.2 (0.3%)	0.2 (0.3%)	0.2 (0.3%)	0.2 (0.3%)	0.2 (0.4%)	0.2 (0.4%)	0.2 (0.4%)	0.2 (0.5%)	0.1 (0.5%)	0.1 (0.5%)	0.1 (0.6%)	0.1 (0.6%)	0.1 (0.7%)	0.1 (0.7%)	0.1 (0.7%)	(0.6%)
Afghanistan	1.7 (3.4%)	1.7 (2.8%)	1.7 (2.8%)	1.7 (2.8%)	1.7 (3.0%)	1.7 (3.2%)	1.7 (3.6%)	1.7 (4.0%)	1.7 (4.4%)	1.7 (5.0%)	1.7 (5.6%)	1.6 (6.3%)	1.5 (6.9%)	1.4 (7.6%)	1.2 (8.2%)	1.1 (8.7%)	0.9 (9.3%)	0.7 (9.9%)	0.6 (10.2%)	0.4 (10.8%)
Algeria	(0.0%)	(0.0%)	(0.0%)	(0.0%)	(0.0%)	(0.0%)	(0.0%)	(0.0%)	(0.0%)	(0.0%)	(0.0%)	(0.0%)	(0.0%)	(0.0%)	(0.0%)	(0.0%)	(0.0%)	(0.0%)	(0.0%)	(0.0%)
Bahrain	(0.0%)	(0.0%)	(0.0%)	(0.0%)	(0.0%)	(0.0%)	(0.0%)	(0.0%)	(0.0%)	(0.0%)	(0.0%)	(0.0%)	(0.0%)	(0.0%)	(0.0%)	(0.0%)	(0.1%)	(0.1%)	(0.1%)	(0.1%)
Egypt	(0.0%)	(0.0%)	(0.0%)	(0.0%)	(0.0%)	(0.0%)	(0.0%)	(0.0%)	(0.0%)	(0.0%)	(0.0%)	(0.0%)	(0.0%)	(0.0%)	(0.1%)	(0.0%)	(0.1%)	(0.0%)	(0.0%)	(0.0%)
Iran	(0.0%)	(0.0%)	(0.0%)	(0.0%)	(0.0%)	(0.0%)	(0.0%)	(0.0%)	(0.0%)	(0.0%)	(0.0%)	(0.0%)	(0.0%)	(0.0%)	(0.0%)	(0.0%)	(0.0%)	(0.0%)	(0.0%)	(0.0%)
Iraq	(0.0%)	(0.0%)	(0.0%)	(0.0%)	(0.0%)	(0.0%)	(0.0%)	(0.0%)	(0.0%)	(0.0%)	(0.0%)	(0.0%)	(0.0%)	(0.0%)	(0.0%)	(0.1%)	(0.0%)	(0.1%)	(0.0%)	(0.0%)
Jordan	(0.0%)	(0.0%)	(0.0%)	(0.0%)	(0.0%)	(0.0%)	(0.0%)	(0.0%)	(0.0%)	(0.0%)	(0.0%)	(0.0%)	(0.0%)	(0.0%)	(0.0%)	(0.0%)	(0.0%)	(0.0%)	(0.0%)	(0.0%)
Kuwait	(0.0%)	(0.0%)	(0.0%)	(0.0%)	(0.0%)	(0.0%)	(0.0%)	(0.0%)	(0.0%)	(0.0%)	(0.0%)	(0.0%)	(0.0%)	(0.0%)	(0.0%)	(0.0%)	(0.0%)	(0.1%)	(0.0%)	(0.1%)
Lebanon	(0.0%)	(0.0%)	(0.0%)	(0.0%)	(0.0%)	(0.0%)	(0.0%)	(0.0%)	(0.0%)	(0.0%)	(0.0%)	(0.0%)	(0.0%)	(0.0%)	(0.0%)	(0.0%)	(0.0%)	(0.0%)	(0.0%)	(0.0%)
Libya	(0.0%)	(0.0%)	(0.0%)	(0.0%)	(0.0%)	(0.0%)	(0.0%)	(0.0%)	(0.0%)	(0.0%)	(0.0%)	(0.0%)	(0.0%)	(0.0%)	(0.0%)	(0.0%)	(0.0%)	(0.0%)	(0.0%)	(0.0%)
Morocco	0.1 (0.2%)	0.1 (0.1%)	0.1 (0.1%)	0.1 (0.1%)	0.1 (0.1%)	0.1 (0.2%)	0.1 (0.2%)	0.1 (0.2%)	0.1 (0.2%)	0.1 (0.2%)	0.1 (0.3%)	0.1 (0.3%)	0.1 (0.3%)	0.1 (0.4%)	0.1 (0.4%)	0.1 (0.4%)	0.1 (0.5%)	(0.5%)	(0.5%)	(0.5%)
Oman	(0.0%)	(0.0%)	(0.0%)	(0.0%)	(0.0%)	(0.0%)	(0.0%)	(0.0%)	(0.0%)	(0.0%)	(0.0%)	(0.0%)	(0.0%)	(0.0%)	(0.0%)	(0.0%)	(0.0%)	(0.0%)	(0.0%)	(0.0%)
Palestine	(0.0%)	(0.0%)	(0.0%)	(0.0%)	(0.0%)	(0.0%)	(0.0%)	(0.0%)	(0.0%)	(0.0%)	(0.0%)	(0.0%)	(0.1%)	(0.1%)	(0.1%)	(0.1%)	(0.1%)	(0.1%)	(0.1%)	(0.1%)
Qatar	(0.0%)	(0.0%)	(0.0%)	(0.0%)	(0.0%)	(0.0%)	(0.0%)	(0.0%)	(0.0%)	(0.0%)	(0.0%)	(0.0%)	(0.0%)	(0.0%)	(0.0%)	(0.0%)	(0.0%)	(0.0%)	(0.0%)	(0.1%)
Saudi Arabia	(0.0%)	(0.0%)	(0.0%)	(0.0%)	(0.0%)	(0.0%)	(0.0%)	(0.0%)	(0.0%)	(0.0%)	(0.0%)	(0.0%)	(0.0%)	(0.0%)	(0.0%)	(0.0%)	(0.0%)	(0.0%)	(0.1%)	(0.1%)
Sudan	0.8 (1.5%)	0.8 (1.2%)	0.8 (1.2%)	0.8 (1.2%)	0.8 (1.3%)	0.8 (1.4%)	0.8 (1.5%)	0.8 (1.7%)	0.8 (1.9%)	0.8 (2.1%)	0.8 (2.3%)	0.8 (2.6%)	0.8 (2.9%)	0.7 (3.2%)	0.7 (3.5%)	0.6 (3.9%)	0.5 (4.2%)	0.4 (4.6%)	0.3 (4.8%)	0.2 (5.0%)
Syria	(0.0%)	(0.0%)	(0.0%)	(0.0%)	(0.0%)	(0.0%)	(0.0%)	(0.0%)	(0.0%)	(0.0%)	(0.0%)	(0.0%)	(0.0%)	(0.0%)	(0.0%)	(0.0%)	(0.1%)	(0.0%)	(0.0%)	(0.0%)
Tunisia	(0.0%)	(0.0%)	(0.0%)	(0.0%)	(0.0%)	(0.0%)	(0.0%)	(0.0%)	(0.0%)	(0.0%)	(0.0%)	(0.0%)	(0.0%)	(0.0%)	(0.0%)	(0.0%)	(0.1%)	(0.0%)	(0.0%)	(0.0%)
Turkey	(0.0%)	(0.0%)	(0.0%)	(0.0%)	(0.0%)	(0.0%)	(0.0%)	(0.0%)	(0.0%)	(0.0%)	(0.0%)	(0.0%)	(0.0%)	(0.0%)	(0.0%)	(0.0%)	(0.0%)	(0.1%)	(0.1%)	(0.0%)
UAE	(0.0%)	(0.0%)	(0.0%)	(0.0%)	(0.0%)	(0.0%)	(0.0%)	(0.0%)	(0.0%)	(0.0%)	(0.0%)	(0.0%)	(0.0%)	(0.0%)	(0.0%)	(0.0%)	(0.0%)	(0.0%)	(0.0%)	(0.0%)
Yemen	0.8 (1.7%)	0.9 (1.3%)	0.9 (1.3%)	0.9 (1.3%)	0.9 (1.4%)	0.9 (1.5%)	0.9 (1.7%)	0.9 (1.8%)	0.9 (2.0%)	0.9 (2.3%)	0.9 (2.6%)	0.8 (2.8%)	0.8 (3.2%)	0.7 (3.5%)	0.7 (3.9%)	0.6 (4.2%)	0.5 (4.6%)	0.4 (4.8%)	0.3 (5.1%)	0.3 (5.2%)

Supplementary Figure 5. Changes in life expectancy by age in North Africa and Middle East in 2019 if ambient PM pollution had been lowered to the theoretical minimum risk exposure levels

	All Ages	Under 1	1 to 4	5 to 9	10 to 14	15 to 19	20 to 24	25 to 29	30 to 34	35 to 39	40 to 44	45 to 49	50 to 54	55 to 59	60 to 64	65 to 69	70 to 74	75 to 79	80 to 84	85 plus
NAME	1.4 (2.8%)	1.5 (2.1%)	1.5 (2.1%)	1.5 (2.1%)	1.5 (2.2%)	1.5 (2.4%)	1.5 (2.6%)	1.5 (2.9%)	1.5 (3.1%)	1.5 (3.5%)	1.4 (3.9%)	1.4 (4.3%)	1.4 (4.8%)	1.3 (5.3%)	1.2 (5.9%)	1.0 (6.4%)	0.9 (6.8%)	0.7 (7.3%)	0.6 (7.7%)	0.4 (8.3%)
Afghanistan	0.5 (1.0%)	0.5 (0.8%)	0.5 (0.8%)	0.5 (0.8%)	0.5 (0.9%)	0.5 (1.0%)	0.5 (1.0%)	0.5 (1.2%)	0.5 (1.3%)	0.5 (1.5%)	0.5 (1.7%)	0.5 (1.8%)	0.4 (2.0%)	0.4 (2.2%)	0.4 (2.4%)	0.3 (2.5%)	0.3 (2.7%)	0.2 (2.9%)	0.2 (3.0%)	0.1 (3.3%)
Algeria	1.1 (2.1%)	1.1 (1.6%)	1.1 (1.6%)	1.1 (1.6%)	1.1 (1.7%)	1.1 (1.8%)	1.1 (2.0%)	1.1 (2.1%)	1.1 (2.3%)	1.1 (2.6%)	1.1 (2.8%)	1.1 (3.1%)	1.0 (3.5%)	1.0 (3.8%)	0.9 (4.2%)	0.8 (4.6%)	0.7 (5.1%)	0.6 (5.7%)	0.4 (6.4%)	0.3 (8.6%)
Bahrain	1.4 (3.1%)	1.5 (2.0%)	1.5 (2.0%)	1.5 (2.0%)	1.5 (2.2%)	1.5 (2.3%)	1.5 (2.5%)	1.5 (2.8%)	1.5 (3.0%)	1.5 (3.4%)	1.4 (3.8%)	1.4 (4.2%)	1.4 (4.8%)	1.3 (5.5%)	1.3 (6.4%)	1.2 (7.5%)	1.1 (8.8%)	0.9 (9.9%)	0.7 (10.7%)	0.5 (12.0%)
Egypt	2.0 (4.1%)	2.1 (3.1%)	2.1 (3.1%)	2.1 (3.1%)	2.1 (3.3%)	2.1 (3.6%)	2.1 (3.9%)	2.1 (4.3%)	2.0 (4.8%)	2.0 (5.3%)	2.0 (5.9%)	1.9 (6.7%)	1.9 (7.6%)	1.7 (8.5%)	1.6 (9.3%)	1.3 (9.9%)	1.1 (10.4%)	0.9 (10.9%)	0.7 (11.1%)	0.5 (11.0%)
Iran	1.2 (2.5%)	1.3 (1.8%)	1.3 (1.8%)	1.3 (1.8%)	1.3 (1.9%)	1.3 (2.0%)	1.3 (2.2%)	1.3 (2.4%)	1.3 (2.6%)	1.3 (2.9%)	1.3 (3.2%)	1.3 (3.5%)	1.2 (4.0%)	1.2 (4.4%)	1.1 (5.0%)	1.0 (5.5%)	0.9 (6.2%)	0.8 (7.1%)	0.7 (7.7%)	0.5 (8.6%)
Iraq	2.0 (3.8%)	2.0 (2.9%)	2.0 (2.9%)	2.0 (2.9%)	2.0 (3.1%)	2.0 (3.4%)	2.0 (3.7%)	2.0 (4.0%)	2.0 (4.5%)	2.0 (5.0%)	2.0 (5.5%)	2.0 (6.3%)	1.9 (7.0%)	1.8 (7.8%)	1.7 (8.6%)	1.5 (9.3%)	1.3 (9.9%)	1.0 (10.4%)	0.8 (10.8%)	0.6 (11.2%)
Jordan	1.1 (2.1%)	1.2 (1.6%)	1.2 (1.6%)	1.2 (1.6%)	1.2 (1.7%)	1.2 (1.8%)	1.2 (2.0%)	1.2 (2.1%)	1.2 (2.3%)	1.2 (2.6%)	1.2 (2.9%)	1.1 (3.2%)	1.1 (3.6%)	1.1 (4.0%)	1.0 (4.5%)	0.9 (5.0%)	0.8 (5.5%)	0.7 (6.1%)	0.6 (6.5%)	0.4 (7.1%)
Kuwait	1.9 (3.6%)	1.9 (2.5%)	1.9 (2.5%)	1.9 (2.5%)	1.9 (2.6%)	1.9 (2.8%)	1.9 (3.1%)	1.9 (3.3%)	1.9 (3.6%)	1.9 (4.0%)	1.9 (4.4%)	1.9 (4.8%)	1.8 (5.4%)	1.7 (6.0%)	1.6 (6.6%)	1.5 (7.4%)	1.4 (8.4%)	1.3 (9.4%)	1.1 (10.6%)	0.9 (12.1%)
Lebanon	1.1 (2.3%)	1.2 (1.7%)	1.2 (1.7%)	1.2 (1.7%)	1.2 (1.8%)	1.2 (1.9%)	1.2 (2.1%)	1.2 (2.3%)	1.2 (2.5%)	1.2 (2.7%)	1.2 (3.0%)	1.1 (3.4%)	1.1 (3.8%)	1.0 (4.2%)	1.0 (4.6%)	0.9 (5.1%)	0.7 (5.5%)	0.6 (5.9%)	0.5 (6.3%)	0.4 (6.8%)
Libya	1.5 (3.0%)	1.5 (2.1%)	1.5 (2.1%)	1.5 (2.2%)	1.5 (2.3%)	1.5 (2.5%)	1.5 (2.7%)	1.6 (2.9%)	1.5 (3.2%)	1.5 (3.5%)	1.5 (3.9%)	1.5 (4.3%)	1.4 (4.7%)	1.3 (5.2%)	1.3 (5.8%)	1.1 (6.3%)	1.0 (6.8%)	0.9 (7.2%)	0.7 (7.3%)	0.5 (8.1%)
Morocco	1.4 (3.0%)	1.4 (2.1%)	1.4 (2.1%)	1.4 (2.1%)	1.4 (2.2%)	1.5 (2.4%)	1.5 (2.6%)	1.5 (2.9%)	1.5 (3.2%)	1.4 (3.6%)	1.4 (4.0%)	1.4 (4.5%)	1.4 (5.0%)	1.3 (5.6%)	1.2 (6.2%)	1.0 (6.8%)	0.9 (7.3%)	0.7 (7.7%)	0.6 (8.0%)	0.4 (8.3%)
Oman	1.5 (3.1%)	1.5 (2.2%)	1.5 (2.2%)	1.5 (2.2%)	1.5 (2.3%)	1.5 (2.5%)	1.5 (2.8%)	1.5 (3.0%)	1.5 (3.3%)	1.5 (3.7%)	1.5 (4.2%)	1.5 (4.8%)	1.5 (5.5%)	1.4 (6.4%)	1.3 (7.4%)	1.2 (8.7%)	1.0 (9.7%)	0.9 (10.8%)	0.7 (11.7%)	0.4 (10.8%)
Palestine	1.3 (2.4%)	1.3 (1.9%)	1.3 (1.9%)	1.3 (1.9%)	1.3 (2.0%)	1.3 (2.2%)	1.3 (2.4%)	1.3 (2.6%)	1.3 (2.9%)	1.3 (3.2%)	1.3 (3.6%)	1.3 (4.0%)	1.3 (4.6%)	1.2 (5.2%)	1.1 (5.9%)	1.0 (6.6%)	0.9 (7.3%)	0.7 (7.8%)	0.6 (8.0%)	0.4 (8.5%)
Qatar	1.3 (2.6%)	1.3 (1.8%)	1.3 (1.8%)	1.3 (1.8%)	1.3 (1.9%)	1.3 (2.1%)	1.3 (2.2%)	1.3 (2.5%)	1.3 (2.7%)	1.3 (3.0%)	1.3 (3.4%)	1.3 (3.8%)	1.2 (4.4%)	1.2 (5.1%)	1.1 (6.0%)	1.1 (7.2%)	0.9 (8.7%)	0.8 (10.5%)	0.6 (13.0%)	0.5 (13.9%)
Saudi Arabia	1.9 (4.1%)	2.0 (2.8%)	2.0 (2.8%)	2.0 (2.8%)	2.0 (3.0%)	2.0 (3.3%)	2.0 (3.6%)	2.0 (3.9%)	2.0 (4.3%)	2.0 (4.8%)	2.0 (5.3%)	1.9 (5.9%)	1.8 (6.6%)	1.7 (7.2%)	1.6 (7.8%)	1.4 (8.4%)	1.2 (8.9%)	1.0 (9.6%)	0.8 (10.2%)	0.6 (11.3%)
Sudan	1.3 (2.4%)	1.3 (1.9%)	1.3 (1.9%)	1.3 (1.9%)	1.3 (2.0%)	1.3 (2.2%)	1.3 (2.4%)	1.3 (2.6%)	1.3 (2.9%)	1.3 (3.2%)	1.3 (3.6%)	1.2 (4.0%)	1.2 (4.5%)	1.1 (5.0%)	1.0 (5.5%)	0.9 (6.0%)	0.8 (6.5%)	0.7 (7.1%)	0.5 (7.4%)	0.4 (7.7%)
Syria	1.3 (2.8%)	1.4 (2.0%)	1.4 (2.0%)	1.4 (2.1%)	1.4 (2.2%)	1.4 (2.4%)	1.4 (2.6%)	1.4 (2.8%)	1.4 (3.1%)	1.4 (3.4%)	1.4 (3.8%)	1.3 (4.2%)	1.3 (4.6%)	1.2 (5.1%)	1.0 (5.6%)	0.9 (6.0%)	0.8 (6.3%)	0.6 (6.8%)	0.4 (7.2%)	0.3 (8.1%)
Tunisia	1.2 (2.5%)	1.3 (1.7%)	1.3 (1.7%)	1.3 (1.7%)	1.3 (1.8%)	1.3 (2.0%)	1.3 (2.2%)	1.3 (2.4%)	1.3 (2.6%)	1.3 (2.8%)	1.3 (3.1%)	1.2 (3.5%)	1.2 (3.9%)	1.1 (4.4%)	1.1 (4.9%)	1.0 (5.5%)	0.9 (6.1%)	0.7 (6.6%)	0.6 (7.2%)	0.4 (7.7%)
Turkey	1.0 (2.2%)	1.1 (1.5%)	1.1 (1.5%)	1.1 (1.5%)	1.1 (1.6%)	1.1 (1.7%)	1.1 (1.8%)	1.1 (2.0%)	1.1 (2.2%)	1.1 (2.4%)	1.1 (2.7%)	1.1 (3.0%)	1.0 (3.3%)	1.0 (3.7%)	0.9 (4.2%)	0.9 (4.7%)	0.8 (5.2%)	0.7 (5.7%)	0.6 (6.2%)	0.4 (6.7%)
UAE	1.4 (3.4%)	1.5 (2.1%)	1.5 (2.1%)	1.5 (2.2%)	1.5 (2.3%)	1.5 (2.5%)	1.5 (2.7%)	1.5 (3.0%)	1.5 (3.3%)	1.5 (3.6%)	1.5 (4.1%)	1.4 (4.6%)	1.4 (5.2%)	1.3 (5.8%)	1.2 (6.5%)	1.1 (7.2%)	0.9 (7.8%)	0.8 (8.8%)	0.7 (9.7%)	0.5 (10.6%)
Yemen	1.0 (2.0%)	1.1 (1.6%)	1.1 (1.6%)	1.1 (1.6%)	1.1 (1.7%)	1.1 (1.9%)	1.1 (2.1%)	1.1 (2.3%)	1.1 (2.5%)	1.1 (2.8%)	1.1 (3.1%)	1.0 (3.5%)	1.0 (3.9%)	0.9 (4.2%)	0.8 (4.6%)	0.7 (5.1%)	0.6 (5.5%)	0.5 (5.8%)	0.4 (6.0%)	0.3 (6.2%)
